# Supplementary material for: Immunogenicity of Tetravalent Protein Vaccine SCTV01E-2 against SARS-CoV-2 EG.5 Subvaraint: A Phase 2 Trial
Source: Vaccines (Basel). 2024 Feb 8;12(2):175. doi: 10.3390/vaccines12020175 (PMC10893468; doi:10.3390/vaccines12020175)
Supplement: Supplementary file 1 [file vaccines-12-00175-s001.zip › Supplementary Materials S2-SCTV01E-2-CHN-1 clinical trial protocol.pdf]

CLINICAL STUDY PROTOCOL

A Phase II Clinical Trial to Evaluate the Immunogenicity and Safety of Beta/ Omicron (BA.1/BQ.1.1/XBB.1) Variants S-Trimer COVID-19 Vaccine (SCTV01E-2) in SARS-CoV-2 Vaccinated Populations

**Protocol ID:**	SCTV01E-2-CHN-1
**Protocol Version No.:**	Version 3.1
**Version Date:**	31 Aug 2023
**Sponsor:**	SionCellTech Ltd.<br>No. 31 Kechuang 7th Street, BDA, Beijing, China<br>Website: www.sinocelltech.com

Confidentiality Statement

All information in this protocol constitutes proprietary property of Sinocelltech Ltd. Therefore, it is only provided to the investigators, co-investigators, ethics committee and regulatory authorities and other relevant medical institutions for review. Without the written approval of Sinocelltech Ltd., it is strictly prohibited to inform any information to a third party unrelated to the study, except for the necessary explanation to the subjects who may participate in the study, when signing the informed consent form.

PROTOCOL SIGNATURE PAGE

I, the undersigned, agree that:

- Follow this study in strict accordance with the protocol, Good Clinical Practice (GCP) and relevant laws and regulations.
- All data and information provided by Sinocelltech Ltd. shall be kept in accordance with confidentiality requirements. When these data and information are submitted to the Institutional Review Board or Independent Ethics Committee, it shall be indicated that these data are confidential.

I have read the full text of this protocol and agree with all information contained herein.

______________________	______________________	______________________
Head of Sponsor	Signature	Date

PROTOCOL SIGNATURE PAGE

I, the undersigned, agree that:

- Follow this study in strict accordance with the protocol, Good Clinical Practice (GCP) and relevant laws and regulations.
- All data and information provided by Sinocelltech Ltd. shall be kept in accordance with confidentiality requirements. When these data and information are submitted to the Institutional Review Board or Independent Ethics Committee, it shall be indicated that these data are confidential.

I have read the full text of this protocol and agree with all information contained herein.

______________________	______________________	______________________
Statistician	Signature	Date

PROTOCOL SIGNATURE PAGE

I, the undersigned, agree that:

- Follow this study in strict accordance with the protocol, Good Clinical Practice (GCP) and relevant laws and regulations.
- All data and information provided by Sinocelltech Ltd. shall be kept in accordance with confidentiality requirements. When these data and information are submitted to the Institutional Review Board or Independent Ethics Committee, it shall be indicated that these data are confidential.

I have read the full text of this protocol and agree with all information contained herein.

____________________	____________________	____________________
Principal Investigator	Signature	Date

PROTOCOL SYNOPSIS

**Protocol ID**	SCTV01E-2-CHN-1
**Title of Study**	A Phase II Clinical Trial to Evaluate the Immunogenicity and Safety of Beta/ Omicron (BA.1/BQ.1.1/XBB.1) Variants S-Trimer COVID-19 Vaccine (SCTV01E-2) for SARS-CoV-2 in SARS-CoV-2 Vaccinated Populations
**Version No.**	Version 3.1
**Version Date**	31 Aug 2023
**Sponsor**	Sinocelltech Ltd.
**Phase of Study**	Phase II
**Indications Investigated**	Prevention of disease caused by infection of SARS-CoV-2
**Study Population**	Subjects aged ≥ 3 years, who have been vaccinated with SARS-CoV-2 vaccine approved for use in China (conditional marketing or emergency authorization) with the recommended dose and immunization schedule, and who are healthy or have a stable underlying disease
**Objective of Study**	**Primary objective:**<br>• To evaluate the immunogenicity of SCTV01E-2 in subjects aged 18 years and above who have been previously vaccinated with SARS-CoV-2 vaccine;<br>• To evaluate the immunogenicity of SCTV01E-2 in subjects aged 3-17 years who have been previously vaccinated with SARS-CoV-2 vaccine;<br>**Secondary objectives:**<br>• To evaluate the safety of SCTV01E-2 in subjects aged 3 years and above who have been previously vaccinated with SARS-CoV-2 vaccine.<br>**Exploratory objectives:**<br>• To evaluate the protective efficacy of SCTV01E-2 against COVID-19 with any symptoms occurring 7 days after vaccination.
**Study Endpoints**	**Primary endpoint:**<br>• Geometric mean titer (GMT) of neutralizing antibody (authentic virus based neutralization assay) against the current predominant strains of SARS-CoV-2 at 14 days after vaccination (the current strains are based on the predominant epidemic types during the assay/serological detection);<br>• Seroresponse rate (SRR) of neutralizing antibodies (authentic virus based neutralization assay) against the current predominant strains of SARS-CoV-2 at 14 days after vaccination;<br><br>**Secondary endpoints:**<br>***Immunogenicity***

	• GMTs and SRRs of neutralizing antibodies against other SARS-CoV-2 variants at 14 days after vaccination (other variant types will be adjusted to reflect changes in epidemic strains);<br>• GMTs and SRRs of neutralizing antibodies against the current predominant strains of SARS-CoV-2 and/or other variants at 180 days after vaccination;<br>***Safety***<br>• Incidence and severity of solicited adverse events (AEs) on Days 0-7 after vaccination;<br>• Incidence and severity of unsolicited AEs on Days 0-28 after vaccination;<br>• Incidence and severity of serious adverse events (SAEs) and adverse events of special interest (AESIs) up to 365 days after vaccination.<br>**Exploratory endpoints:**<br>• Number of events with any symptomatic COVID-19 occuring for the first time 7 days (≥ 8 days) after vaccination.
**Background of the Study**	In response to the rapid mutation of SARS-CoV-2 variants and the decrease in neutralizing antibody titers and protection rate of first generation vaccines based on the original SARS-CoV-2 strain both in China and overseas, Sinocelltech Ltd. has developed a series of vaccines against SARS-CoV-2 variants, including SCTV01C, SCTV01E and SCTV01E-2, all of which are recombinant protein vaccines against SARS-CoV-2 variants independently developed by adopting the genetic engineering technology on the same CHO cell production platform. SCTV01C contains a 2-valent recombinant S trimer protein antigen based on Alpha and Beta variants, and SCTV01E is a 4-valent modified vaccine comprising Delta and Omicron BA.1 variant S trimer protein antigens in addition to SCTV01C's antigen compoents. SCTV01E-2 contains the S trimer protein antigens based on Beta and Omicron BA.1 variants, and adds the S trimer protein antigens based on BQ.1.1 and XBB.1 variants, constituting a new, **4-valent modified vaccine**. Both SCTV01C and SCTV01E have been approved for emergency use in China. SCTV01E-2 has the same manufacturing process, molecular design basis and clinical dosage as SCTV01E. Currently, SCTV01E has been studied in four clinical studies worldwide, and the accrued safety data from nearly 6,000 subjects vaccinated with SCTV01E show that SCTV01E has no safety risk. In a phase III immunogenicity study, SCTV01E showed superior immunogenicity compared with Pfizer mRNA SARS-CoV-2 vaccine. The above studies are supporting evidence for the design and initiation of SCTV01E-2 clinical evaluation.
**Study Design**	**Overall design:**<br>This is an immunogenicity bridging phase II clinical trial of SCTV01E-2 with its predecessor SCTV01E. We aimed to

	evaluate the immunogenicity and safety of SCTV01E-2 in subjects of different age groups, who have been vaccinated with approved SARS-CoV-2 vaccine. SCTV01E has completed a phase III protective efficacy study and has been approved for emergency use in China. SCTV01E-2 is an update modified version of SCTV01E.<br>A total of at least 600 subjects ≥ 3 years of age previously vaccinated with SARS-CoV-2 vaccine approved for domestic use (conditional marketing or emergency authorization for use) with the recommended dose and immunization schedule are planned to be included in this study. Subjects aged 18 years and above are included in Group A, and subjects aged 3-17 years are included in Group B. Subjects will be tested for baseline IgM before vaccination, and the number of subjects with positive baseline IgM should be supplemented to ensure a minimum of 400 subjects with negative baseline IgM in Group A and 200 subjects with negative baseline IgM in Group B. In addition, subjects may be appropriately enrolled based on the percentage of subjects infected with SARS-CoV-2 within 14 days of vaccination (prior to D14 immunogenicity sampling).<br>All subjects in Group A will be randomized 1:1 to receive one dose of SCTV01E-2 or SCTV01E after enrollment. Fourteen sentinel subjects aged 18-59 will be observed for 7 days following administration of the investigational vaccine for safety assessment by IDMC, and if the criteria for suspension/termination of the study are not met, non-sentinel subjects in Group A will continue to be enrolled and enrollment in Group B will then be initiated.<br>Randomization stratification factors for Group A are age (18- 59 years, ≥ 60 years), history of SARS-CoV-2 infection (yes, no), and interval of previous vaccination/infection (6-11 months, ≥ 12 months). The interval between previous vaccination/infection is defined as the interval between the time of the last dose of vaccination/last SARS-CoV-2 infection (whichever is later) and the time of ICF signing. In Group A, the proportion of subjects' ≥ 60 years of age shall be no less than 40%.<br>Group B is divided into the following age groups: 3-5 years, 6-11 years, 12-17 years, and all subjects are vaccinated with one dose of SCTV01E-2. Fourteen sentinel subjects per age group will be enrolled sequentially in order of decreasing age. Initially, 14 sentinel subjects aged 12-17 years will be enrolled and observed for 7 days after receiving the investigational vaccine. Following IDMC's safety assessment, if the criteria for suspension/termination of the study are not met, non-sentinel subjects aged 12-17 years and 14 sentinel subjects aged 6-11 years will be enrolled simultaneously. Furthermore, sentinel subjects aged 6-11 years will be observed for 7 days after being vaccinated with the investigational vaccine for safety assessment by IDMC, and if the criteria for

suspension/termination of the study are not met, non-sentinel subjects aged 6-11 years and 14 sentinel subjects aged 3-5 years will be enrolled simultaneously. Finally, sentinel subjects aged 3-5 years will be observed for 7 days after being vaccinated with the investigational vaccine for safety assessment by IDMC, and non-sentinel subjects aged 3-5 years will be enrolled if the criteria for suspension/termination of the study are not met.

The study design is provided in **Figure 1**.

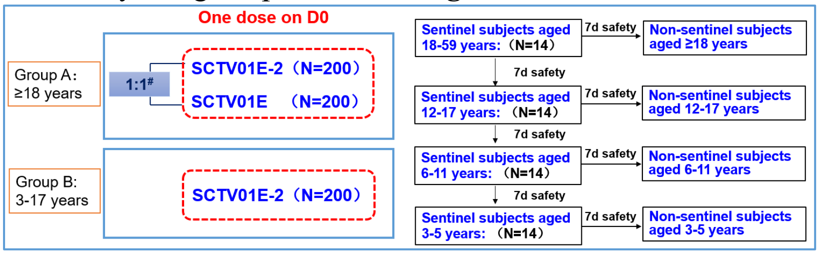

Figure 1 The Schema of Study Design

Screening period:

After the subjects sign ICF, a screening visit will be conducted within 7 days prior to study vaccination to review eligibility.

Randomization:

Subjects eligible for screening in Group A will be randomized prior to vaccination.

Safety follow-up:

Subjects will be observed at the study site for at least 30 minutes after study vaccination (which could be adjusted by the investigator according to the subjects’ condition), and the solicited and unsolicited AEs at the vaccination site (local) and non-vaccination site (systemic) will be reported during this period. A combination of active surveillance and spontaneous reporting is used to collect solicited AEs for up to 7 days and unsolicited AEs for up to 28 days after vaccination for all subjects, as well as SAEs and AESIs for up to 365 days after study vaccination. 28 days after vaccination, subjects will be followed up by regular telephone calls at a frequency of at least once a month, which may be adjusted as appropriate.

The enrollment of additional subjects in the corresponding age group will continue after the sentinel subject has completed the 7-day post-vaccination safety assessment. Meanwhile, the first 20 subjects in each age group (≥ 18 years, 12-17 years, 6-11 years, and 3-5 years) will be sampled for laboratory tests on Day 3 after vaccination.

Follow-up for immunogenicity:

Blood samples will be collected from subjects prior to study vaccination, on Day 14 and Day 180 post-vaccination for the detection of total IgG antibodies against SARS-CoV-2 (pre-vaccination only) and for the detection of neutralizing antibody titers against the current predominant strains and other variants of SARS-CoV-2 (the current predominant strains are based on the predominant epidemic types during the assay/serological detection, and the other variants are adjusted

	according to the changes in the epidemic strains).<br>**Follow-up for protective efficacy:**<br>Follow-up for protective efficacy will begin after vaccination. Study staff will periodically ask subjects for signs/symptoms related to COVID-19 by phone call, short message (sms), email, or during on-site visits. The frequency of follow-up for protective efficacy may be adjusted according to the progress of the trial. Meanwhile, subjects can spontaneously report any COVID-19-related symptoms at any time during the study. Subjects will undergo SARS-CoV-2 antigen or nucleic acid test if any of the relevant COVID-19 clinical symptoms is/are met.<br>If a subject receives or requires any other SARS-CoV-2 vaccine during the study, he/she should be withdrawn from the study.
**Number of Subjects**	A minimum of 600 subjects are planned to be enrolled, including at least 400 subjects ≥ 18 years and at least 200 subjects between 3 - 17 years. The proportion of subjects with IgM positive at baseline and/or other factors will influence the actual enrollment numbers.
**Study Duration**	The duration of study participation for each subject will be approximately 365 days.
**Inclusion Criteria**	**Only subjects who meet all of the following criteria will be included in the study:**<br>1) Aged ≥ 3 years at the time of signing the informed consent form;<br>2) Subjects who have been vaccinated with the SARS-CoV-2 vaccine approved for domestic use (conditional marketing or emergency authorization for use) with the recommended dose and immunization schedule, and the interval from the last dose of SARS-CoV-2 vaccine to signing the ICF is ≥ 6 months;<br>3) Subjects' and/or their legal guardians or delegates are able to sign the written informed consent form and voluntarily participate in the trial, and can fully understand the trial procedures, the risks of participating in the trial and other available interventions if not participating in the trial;<br>4) Subjects' and/or their legal guardians or delegates are able to read, understand, and complete the diary/contact card;<br>5) Healthy subjects or subjects with stable underlying diseases. Stable underlying disease is defined as a stable condition for at least 3 months prior to enrollment in this study, with no significant changes in treatment plan, and no hospital admission due to disease progression;<br>6) Males and females of childbearing potential who voluntarily agree to take effective contraceptive measures from date of signing theICF to 6 months after administration of investigational vaccine; Females of childbearing potential who have a negative pregnancy test

	at screening.
**Exclusion Criteria**	**A subject fulfilling any of the following criteria will be excluded from the study:**<br>1) Fever (for > 14 years old, axillary temperature ≥ 37.3℃; for ≤ 14 years old, axillary temperature ≥ 37.5℃) within 72 hours prior to administration of the study vaccine;<br>2) Positive SARS-CoV-2 nucleic acid or antigen test at screening;<br>3) Known SARS-CoV-2 prior infection (including asymptomatic and symptomatic infected patients) within 6 months prior to signing the ICF;<br>4) History of severe allergy, such as severe skin eczema, dyspnea, throat edema, angioneurotic edema, etc., or allergy to the investigational vaccine and its components;<br>5) Subjects' with history or family history of convulsion, epilepsy and psychosis;<br>6) Subjects' who are in an acute state of illness, such as acute onset of chronic heart failure, acute sore throat, hypertensive encephalopathy, acute pneumonia, acute renal insufficiency, acute cholecystitis;<br>7) Subjects' currently have contraindications to intramuscular injection or venous blood sampling, such as thrombocytopenia or other coagulation disorders;<br>8) Subjects' on antituberculosis therapy;<br>9) Subjects' who have been vaccinated with influenza vaccine within 14 days prior to study vaccination or with other vaccine within 28 days prior to study vaccination;<br>10) Subjects who have received investigational drug treatment of other clinical trial within 1 month before study vaccination;<br>11) Subjects who have received any immunoglobulin product or blood product within the previous 3 months, or plan to receive similar products during the study;<br>12) Subjects who donated blood or had blood loss (≥ 450 mL) within 3 months before the study vaccination or plan to donate blood during the study;<br>13) Pregnancy or nursing;<br>14) Subjects' who plan to donate ovum or sperms during the study period;<br>15) Subjects' who are unable to comply with trial procedures, or cooperate to complete the study due to planned relocation or long-term outing;<br>16) Subjects' deemed by the investigator as unfit to participate in the clinical trial because of abnormalities that are likely to distort the study results, or not in best interest of the subjects (inability to obtain maximum benefits);
**Withdrawal Criteria**	**Voluntary withdrawal by subject**<br>Subjects have the right to withdraw from the study prematurely

	at any stage. For subjects who withdraw prematurely, the investigator should make every effort to contact the subject, record the reason for withdrawal in the source document, and inform the sponsor's team.<br><br>**Withdrawal by investigator:**<br><br>This refers to an already enrolled subject who subsequently becomes unfit to continue in the course of the study. The said subject may withdraw from the study based on the decision of investigator. The reasons may include:<br><br>1) The subject has intolerable adverse events, and the continuation of the trial is harmful to the subjects' health as judged by the investigator;<br>2) The serious protocol deviations may affect the safety of subjects';<br>3) Other reasons judged by the investigator as unsuitable for subjects' continued participation in the study<br><br>**Handling of subject withdrawal:**<br><br>Investigators should make every effort to contact subjects who do not come to the center for regular follow-up visits. Subjects who withdraw from the study due to SAEs or AEs will be followed by the investigator until the adverse events resolve /subjects recover, being stable or having other outcome. If subjects withdraw from the study, nose/nasopharynx/oropharynx swab samples and blood samples collected prior to the date of withdrawal may still be available for study analysis, unless otherwise expressely requested by the subjects.<br><br>The investigator should record the relevant information of withdrawal in the eCRF, including whether the withdrawal from the study is at the discretion of the subject or the investigator, and provide detailed description of the specific situation:<br><br>1) Lost to follow-up;<br>2) In case of death, the investigator should record the cause of death;<br>3) If the subject withdraws voluntarily, the investigator should record the situation and reason for withdrawal:<br>• Withdrawal situation:<br>a) Subjects withdraw voluntarily and completely from the study, including the collection of biological samples and safety observation;<br>b) Subjects withdraw voluntarily from part of the study, e.g. only the collection of biological samples is not performed, other studies specified in the protocol should be continued;<br>• Withdrawal reasons:<br>a) The subject requests to withdraw from the study for study-related reasons, such as inability to

	tolerate adverse events, biological sample collection, etc.;<br>b) The subject requests to withdraw from the study for study-unrelated reasons, such as long-term outing, relocation, etc.<br>4) Withdrawal from the study as judged by the investigator;<br>5) Other reasons.<br>If a subject withdraws or is discontinued from the study after enrollment (including lost to follow-up), the subject should not be replaced.
**Suspension/Termination Criteria**	**Criteria for study suspension**<br>If subjects in each age group (≥ 18 years, 12-17 years, 6-11 years, 3-5 years) experience any of the following situations, the trial should be suspended and the investigator, sponsor, and IDMC should hold a meeting to decide whether to adjust the clinical trial:<br>• Grade 3 or higher AEs related to the investigational vaccine occurring in ≥ 15% of subjects who received the investigational vaccine;<br>• Any Grade 4 or higher AEs related to the investigational vaccine occurred during the study;<br>• Any SUSARs related to the investigational vaccine occurred during the study.<br>• For specified AEs, the investigator and the sponsor will decide whether to suspend or terminate on a case-by-case basis to ensure subject safety.<br>**Discontinuation criteria**<br>In case of any of the following circumstances, the trial should be terminated:<br>• The sponsor requests to completely terminate the trial with justification.<br>• The Ethics Committee requests to completely terminate the trial and gives reasons;<br>• The Drug Regulatory Authority requests to completely terminate the trial and gives reasons.
**Investigational Vaccine**	1. **Study vaccine: Recombinant S-Trimer Protein Subunit [Beta/Omicron (BA.1/BQ.1. 1/XBB.1) Variant] Vaccine for SARS-CoV-2 (SCTV01E-2)**<br>• Appearance: milky, white suspension liquid;<br>• Ingredients:<br>- Main active ingredients: TM23 protein, TM41 protein, TM41F protein, TM41H protein;<br>- Adjuvants: SCT-VA02B, which is an oil-in-water emulsion containing squalene;<br>- Excipients: citric acid, sodium citrate, sodium chloride, Polysorbate 80.<br>• Dosage form: injection;

	• Strength: 0.5 mL/vial (for 1 person);<br>• Route of vaccination: intramuscular injection into the lateral deltoid of the upper arm;<br>• Vaccination dose: 30 μg/0.5 mL;<br>• Immunization schedule: 1 dose on the day of vaccination;<br>• Storage conditions: Store and transport at 2 - 8°C, protected from light;<br>• Shelf life: 24 months;<br>• Manufacturer: Sinocelltech Ltd.<br>**2. Control vaccine: Recombinant S-Trimer Protein Subunit (Alpha/Beta/Delta/Omicron Variant) Vaccine for SARS-CoV-2 (SCTV01E)**<br>• Appearance: milky, white suspension liquid;<br>• Ingredients:<br>– Main active ingredients: TM22 protein, TM23 protein, TM28 protein, TM41 protein;<br>– Adjuvants: SCT-VA02B, which is an oil-in-water emulsion containing squalene;<br>– Excipients: citric acid, sodium citrate, sodium chloride, Polysorbate 80.<br>• Dosage form: injection;<br>• Strength: 0.5 mL/vial (for 1 person);<br>• Route of vaccination: intramuscular injection into the lateral deltoid of the upper arm;<br>• Vaccination dose: 30 μg/0.5 mL;<br>• Immunization schedule: 1 dose on the day of vaccination;<br>• Storage conditions: Store and transport at 2 - 8°C, protected from light;<br>• Shelf life: 24 months;<br>• Manufacturer: Sinocelltech Ltd.<br>The above investigational and control vaccine are provided by the sponsor.
**Analysis in Phases**	During the study, the analysis, summary and submission supporting may be performed by the independent unblinded team of the sponsor. The specific analysis time point will be adjusted according to the clinical study progress. The unblinded team members will not participate in the subsequent implementation of the project.
**Statistical Analysis**	**<u>Statistical hypotheses</u>**<br>The following two co-primary endpoints will be considered in the comparison of SCTV01E-2 versus SCTV01E in the part of Group A and the bridging of SCTV01E-2 between 3 - 17 years in Group B and SCTV01E-2 ≥ 18 years in Group A:<br>1. Geometric mean titer (GMTs) of neutralizing antibody of authentic virus after 14 days of vaccination;<br>2. Seroresponse rate (SRR) of neutralizing antibody of authentic virus after 14 days of vaccination. SRR is defined

as the proportion of subjects with a shift in antibody titer from below the lower limit of quantification (LLOQ) on the day of vaccination (pre-vaccination on Day 0) to ≥ LLOQ post-vaccination or from > LLOQ pre-vaccination to ≥ 4 times the baseline value post-vaccination.

For GMT endpoints:

- The geometric mean ratio of neutralizing antibody titers for SCTV01E-2 to SCTV01E in Group A will be recorded as GMR_A;
- The geometric mean ratio of neutralizing antibody titers for SCTV01E-2 in Group B (3-17 years) and SCTV01E-2 in Group A (≥ 18 years) will be recorded as GMR_B;

For SRR endpoints:

- The difference of SRR between SCTV01E-2 and SCTV01E in Group A will be recorded as Δ_A;
- The difference in SRR between SCTV01E-2 in Group B (3-17 years) and SCTV01E-2 in Group A (≥ 18 years) will be recorded as Δ_B.

For Group A, the following superiority hypotheses will be made for GMR_A versus Δ_A:

- For GMR_A, statistical hypothesis test for superiority will be conducted:

H0: $GMR_A \leq 1$, H1: $GMR_A > 1$ (1)

- For Δ_A, statistical hypothesis test for superiority will be conducted:

H0: $\triangle_A \leq 0\%$, H1: $\triangle_A > 0\%$ (2)

For Group B, the following non-inferiority hypotheses will be made for GMR_B versus Δ_B:

- For GMR_B, statistical hypothesis test will be conducted:

H0: $GMR_B \leq 0.67$, H1: $GMR_B > 0.67$ (3)

- For Δ_B, statistical hypothesis test will be conducted:

H0: $\triangle_B \leq -5\%$, H1: $\triangle_B > -5\%$ (4)

Statistical tests for (3) and (4) will be performed only if both tests (1) and (2) are statistical significant at a one-sided significance level of 0.025.

In addition, as secondary endpoints, the ratio of neutralizing antibodies against the current predominant strain of SARS-CoV-2 after 14 days of vaccination with SCTV01E-2 in Group A (≥ 18 years) and anti-BA.5 neutralizing antibodies after 14 days of vaccination with SCTV01E in the immune subgroup in the SCTV01E Phase III study (SCTV01E-MRCT-2) will be recorded as GMR_C, and the difference of SRRs will be recorded as Δ_C.

The following non-inferiority statistical hypotheses will be made for GMR_C and Δ_C:

- For GMR_C, statistical hypothesis testing will be conducted:

H0: $GMR_C \leq 0.67$, H1: $GMR_C > 0.67$ (5)

	• For Δ_C, statistical hypothesis testing will be conducted:<br>**H0: $\triangle_C \leq$ -10%, H1: $\triangle_C >$ -10% (6)**<br>**<u>Sample size</u>**<br>For the hypothesis test (1), based on the following hypothesis, approximately 288 subjects in the test group and control group, the trial has a power of 90% to reject H0 and achieve the superiority:<br>• Difference in Log_{10} transformed standard of variants in two groups: 0.45<br>• GMR_A in both groups: 1.5<br>• Significance level: one-sided 0.025<br>• Dropout rate: 5%<br>• Between-group rate: 1:1<br>For the hypothesis testing (2), based on the following hypothesis, approximately 388 subjects are included in the test group and the control group, and the trial has a power of 90% to reject H0 and achieve the superiority:<br>• The seroresponse rate for SCTV01E-2 is 80%<br>• The seroresponse rate for SCTV01E is 65%<br>• Significance level: one-sided 0.025<br>• Dropout rate: 5%<br>• Between-group rate: 1:1<br>For the hypothesis testing (3), based on the following hypothesis, approximately 300 subjects are included in the test group and the control group, and the trial has a power of 90% to reject H0 and achieve the non-inferiority:<br>• Difference in Log_{10} transformed common standard of variants in two groups: 0.45<br>• GMR_B in two groups: 1<br>• Non-inferiority margin: 0.67<br>• Significance level: one-sided 0.025<br>• Dropout rate: 5%<br>• Between-group rate: 1:1<br>For the hypothesis testing (4), based on the following hypothesis, approximately 392 subjects are included in the test group and the control group, and the trial has a power of 80% to reject H0 and achieve the non-inferiority:<br>• The seroresponse rate for SCTV01E-2 is 80% in Group A with ≥ 18 years of age<br>• The seroresponse rate for SCTV01E-2 is 86% in Group B with 3 - 17 years of age<br>• Non-inferiority margin: -5%<br>• Significance level: one-sided 0.025<br>• Dropout rate: 5%<br>• Between-group rate: 1:1<br>For the hypothesis testing (5), based on the following hypothesis, approximately 368 subjects (at least 184 subjects

	of ≥ 18 years of age in Group A are treated with SCTV01E-2) are included in the test group and the control group, and the trial has a power of 90% to reject H0 and achieve the non-inferiority:<br>• Difference in Log_{10} transformed common standard of variants in two groups: 0.5<br>• GMR_C in two groups: 1<br>• Non-inferiority margin: 0.67<br>• Significance level: one-sided 0.025<br>• Dropout rate: 5%<br>• Between-group rate: 1:1<br>For the hypothesis testing (6), based on the following hypothesis, approximately 386 subjects (at least 193 subjects with ≥ 18 years of age in Group A are treated with SCTV01E-2) are included in the test group and the control group, and the trial has a power of 80% to reject H0 and achieve the non-inferiority:<br>• The seroresponse rate for SCTV01E-2 is 80% in Group A with ≥ 18 years of age<br>• The seroresponse rate in phase III immune subgroup BA.5 is 78%<br>• Non-inferiority margin: -10%<br>• Significance level: one-sided 0.025<br>• Dropout rate: 5%<br>• Between-group rate: 1:1<br>Based on the above 6 hypothesis tests, a total of 600 subjects are planned to be enrolled in this trial, of which 400 subjects will be enrolled in Group A and randomized to SCTV01E or SCTV01E-2 groups at a ratio of 1:1; 200 subjects will be enrolled in Group B. The sample size may be adjusted based on the proportion of subjects who are IgM positive at baseline, infection within 14 days of vaccination, and information from outside the trial, to ensure an adequate evaluable population.<br>**<u>Definitions of analysis populations</u>**<br>Full analysis set (FAS): all vaccinated subjects. In the analysis, subjects will be grouped based on the group to which they are randomized.<br>Per-protocol set (PPS): subjects included in the full analysis set who do not have any major protocol violations affecting the key data of the trial. Major protocol deviations affecting critical data of the trial will be identified and archived prior to trial database lock.<br>Safety set (SS): All subjects who received the study vaccine. In the analysis, subjects will be grouped according to the vaccine actually vaccinated.<br>Immunogenicity full analysis set (I-FAS): subjects included in the full analysis set who have valid immunogenicity test data before and after immunization.

	Immunogenicity per-protocol set (I-PPS): subjects included in the PPS who have valid immunogenicity test data before and after immunization and are IgM negative at baseline.<br>SCTV01E phase III (SCTV01E-MRCT-2) immunogenicity per-protocol set (I-PPS): subjects included in the immunogenicity per-protocol set who have valid immunogenicity test data before and after immunization.<br>The post-infection immunogenicity data from subjects with definite evidence of infection prior to the targeted visit for analysis will be excluded from the analysis.<br>**Statistical methods**<br>**Immunogenicity analysis:**<br>Immunogenicity data will be analyzed separately based on the I-PPS set.<br>For neutralizing antibodies at each visit after vaccination, the GMT and the corresponding 95% CI will be calculated for both groups. Based on the antibody titer after Log_{10} transformation, the ANCOVA model is used to calculate the LS (least squares) GMT for each group, as well as the GMR and corresponding 95% CI for between-group comparisons. Covariates include random stratification factors and the baseline level after Log_{10} transformation.<br>For neutralizing antibodies at each visit after vaccination, SRR and corresponding 95% CI (Clopper-Pearson exact method) will be calculated for both groups. Meanwhile, the rate difference of SRR between the two groups will be calculated along with 95% CI (stratified Miettinen-Nurminen method).<br>**Safety analysis:**<br>Safety data will be analyzed based on the SS set. The medical coding of AEs is performed using the Medical Dictionary for Regulatory Activities (MedDRA). For AEs that occur after immunization or have increased severity compared to pre-immunization, the number and percentage of AEs will be statistically analyzed by two levels: system organ classification (SOC) and preferred term (PT).
**IDMC**	Sponsor will establish an IDMC to review safety data from the clinical trial. IDMC members include experts in the clinical research field of vaccine, biostatisticians and epidemiologists, etc. For the detailed information on the IDMC working documents, please refer to the "IDMC charter".

FLOW CHART

Table 1 Schedule of Visits

	Screening Period	Vaccination Period	Follow-up Period					
**Visit**	**V1**	**V2**	**V3※**	**V4**	**V5**	**V6**	**V7**	**V8▲**
**Planned visit day**	**D-7～D0**	**D0**	**D3**	**D7**	**D14**	**D28**	**D180**	**D365 (EOS)**
**Visit window period**	/	/	+1d	+3d	+3d	+7d	±20d	±20d
**Management and general procedures**								
Sign the informed consent form (ICF)	●							
Assign the screening number	●							
Review the inclusion/exclusion criteria[1]	●	●★						
Demographics [2]	●							
Record the medical history [3]	●							
Vital signs [4]	●	●★						
Physical examination [5]	●							
SARS-CoV-2 rapid antigen test or nucleic acid test for nasal/nasopharyngeal/oropharyngeal swabs [6]	●			●	●	Collected when the COVID-19 diagnosis process is triggered, refer to section 8.3 of the protocol		
Pregnancy test (WOCBP only) [7]	●	●★						
Anti-SARS-CoV-2 IgM test		●						
Randomization [8]		●						
Vaccination		●						
**Blood sampling for immunogenicity assessment**								
Detection of total anti-SARS-CoV-2 IgG antibodies [9]		●						
Detection of neutralizing antibodies against current predominant strains and other variants of SARS-CoV-2 [10]		●			●		●	
**Follow-up of protective efficacy**								
Follow-up of protective efficacy		Once a week by phone call, text message, email, or on-site visit.<br>The frequency of follow-up may be adjusted according to the progress of the study.						
**Safety follow-up**								
Laboratory parameters (urinalysis, hematology, and blood chemistry) [11]		●	●					
Observe for at least 30 minutes after vaccination (which may be adjusted by the investigator according to the individual conditions of the subject)		●						
Distribute thermometer and ruler		●						
Distribute VRCs/contact cards [12]		●		●				
Solicited and unsolicited AEs;		●	●	●	●	●		
SAEs and AESIs [13]		●	●	●	●	●	●	●
Review/recover VRCs/contact cards [14]			●	●	●	●		
Record the concomitant medications		●	●	●	●	●	●#	●#

Abbreviations: **AE**: adverse event; **AESI**: adverse event of special interest; **SAE**: serious adverse event; **VRC**: vaccination report card.

Notes:

※: V3 visit is only applicable to the first 20 subjects in each age group (≥ 18 years, 12 - 17 years, 6 - 11

years, 3 - 5 years).

▲: Visit V8 can be conducted remotely by telephone or on-site follow-up if necessary.

★: If screening and dosing occur on the same day, the items marked with "●★" do not need to be repeated before vaccination.

#: Only concomitant and prohibited medications for the treatment of vaccine-related SAEs and AESIs should be recorded after 28 days post immunisation.

1) Review of inclusion/exclusion criteria will be performed at screening and prior to dosing on the day of dosing.
2) Demographic data: including date of birth (age is calculated based on date of birth), gender, ethnicity, height, weight and BMI (calculated based on height and weight). Contact information such as current telephone numbers and/or e-mails should also be provided. If the contact information is changed at subsequent visits, it should be updated (if applicable).
3) Record the medical history: including SARS-CoV-2 vaccination history, SARS-CoV-2 infection history, other vaccinations and drug therapy history within 28 days before signing ICF, previous major surgical history, allergic history and important disease history.
4) Vital signs: including blood pressure (BP), respiratory rate, pulse and body temperature.
5) Physical examination: including general conditions, head and neck, lymph nodes, skin, chest, abdomen, musculoskeletal system, and other examinations necessary for the study deemed by the investigator.
6) SARS-CoV-2 rapid antigen test or nucleic acid test for nasal/nasopharyngeal/oropharyngeal swabs: All subjects should undergo rapid antigen or nucleic acid testing during screening at the study site. Subjects with positive results will be excluded from this study.
7) Pregnancy test: for WOCBP only (the definition is provided in Appendix I), a urine pregnancy test will be performed routinely, and a blood pregnancy test may be performed if deemed necessary by the investigator.
8) Randomization: applicable to subjects in Group A only.
9) Detection of total anti-SARS-CoV-2 IgG antibodies: The blood sample collection are conducted before vaccination on the day of vaccination.
10) Detection of neutralizing antibodies against current predominant strains and other variants of SARS-CoV-2: The blood samples are collected before vaccination on the day of vaccination, on day 14 post vaccination and on day 180 post vaccination. The authentic virus based neutralization assay is used (the pseudovirus based neutralization assay may be added for detection according to actual needs). The current predominant strains are the predominant epidemic types at the time of conduting the trial/serological testing. The types of other variants are adjusted according to the changes of epidemic strains.
11) Laboratory tests [only for the first 20 subjects in each age group (≥ 18 years, 12 - 17 years, 6 - 11 years, 3 - 5 years)]: Blood samples will be collected before vaccination on the day of vaccination and on day 3 after vaccination.
    - Urinalysis: including protein urine, glucose urine, red blood cells urine, white blood cells urine;
    - Hematology: Percentages and absolute values of white blood cell count (WBC), red blood cell count (RBC), hemoglobin (HGB), platelet (PLT), eosinophil (EOS), neutrophil (NEUT) and lymphocyte (LYM);
    - Blood chemistry: aspartate aminotransferase (AST), alanine aminotransferase (ALT), alkaline phosphatase (ALP), creatine kinase (CPK), total bilirubin (TBIL), total protein, albumin, urea nitrogen (BUN), creatinine (Cr), fasting blood glucose, sodium, potassium, calcium, magnesium and phosphorus;
12) Distribute vaccination report cards (VRCs)/contact cards: Distribute VRCs after vaccination and contact cards at the visit V4.
13) SAEs and AESIs: The safety follow-up for patients are conducted by telephone after 28 days post vaccination at a frequency of at least monthly.
14) Review/Recover VRCs/contact cards: On day 3 after study vaccination, review but not recover VRCs for the first 20 subjects in each age group; On day 7 after study vaccination, review and recover VRCs; On day 14 after study vaccination, review but not recover contact cards; On day 28 after study vaccination, review and recover contact cards.

Table of Contents

CLINICAL STUDY PROTOCOL I

PROTOCOL SIGNATURE PAGE II

PROTOCOL SIGNATURE PAGE III

PROTOCOL SIGNATURE PAGE IV

PROTOCOL SYNOPSIS 1

FLOW CHART 14

TABLE OF CONTENTS 16

LIST OF TABLES 18

LIST OF FIGURES 19

ABBREVIATIONS 20

1 STUDY BACKGROUND 22

1.1 BACKGROUND 22
1.2 ETIOLOGICAL CHARACTERISTICS 23
1.3 INTRODUCTION TO THE STUDY VACCINE 24
1.4 INTRODUCTION TO PRE-CLINICAL STUDIES 25
1.5 CLINICAL SAFETY AND IMMUNOGENICITY OF SCTV01E AND SCTV01C 31
1.6 SAFETY OF SCT-VA02B ADJUVANT 45
1.7 STUDY SIGNIFICANCE 45
1.8 BENEFIT-RISK ASSESSMENT 45

2 STUDY OBJECTIVES AND ENDPOINTS 48

2.1 STUDY OBJECTIVES 48
2.2 STUDY ENDPOINTS 48

3 STUDY DESIGN 50

3.1 STUDY DESIGN 50
3.2 RATIONALE FOR STUDY DESIGN 52
3.3 BLINDING AND UNBLINDING PROTOCOL 53
3.4 DEFINITION OF END OF STUDY 54

4 STUDY POPULATION 55

4.1 INCLUSION CRITERIA 55
4.2 EXCLUSION CRITERIA 55
4.3 DEFINITION OF SCREENING FAILURES 56
4.4 WITHDRAWAL CRITERIA 57
4.5 STUDY SUSPENSION/TERMINATION CRITERIA 58

5 DESCRIPTION OF STUDY PROCEDURES AND VISITS 59

5.1 V1 (SCREENING PERIOD, D-7 TO D0) 59
5.2 V2 (D0, VACCINATION PERIOD) 60
5.3 V3 (D3 + 1D) 61
5.4 V4 (D7+3D) 61
5.5 V5 (D14+3D) 62
5.6 V6 (D28+7D) 62
5.7 V7 (D180 ± 20D) 63
5.8 V8 (EOS, D365 ± 20D) 63
5.9 UNSCHEDULED CONTACT AND FOLLOW-UP 63

6 STUDY VACCINES 64

6.1 BASIC INFORMATION ON STUDY VACCINES 64
6.2 MANAGEMENT OF STUDY VACCINES 65
6.3 ROUTE OF VACCINATION AND DOSE 65

6.4 VACCINATION SCHEDULE 66
6.5 CONCOMITANT MEDICATION 66
6.6 COMPLIANCE OF SUBJECTS 67
7 COLLECTION, DISPOSAL, AND TEST OF BIOLOGICAL SAMPLES 68
7.1 COLLECTION AND DISPOSAL OF BLOOD SAMPLES 68
7.2 COLLECTION, DISPOSAL, AND TEST OF VIRAL TEST SAMPLES 69
7.3 OTHER LABORATORY TESTS 69
8 STUDY ASSESSMENT AND REPORT 71
8.1 SAFETY ASSESSMENT 71
8.2 IMMUNOGENICITY ASSESSMENT INDICATORS 79
8.3 DIAGNOSIS AND TREATMENT OF COVID-19 80
9 DATA MANAGEMENT 84
9.1 Completion and transfer of source data and eCRFs 84
9.2 Suggestion and design of database 84
9.3 Data entry 84
9.4 Data verification and query handling 85
9.5 Medical coding 85
9.6 External data consistency verification 86
9.7 Database locking and exporting 86
9.8 Retention of study records 86
10 STATISTICAL ANALYSIS 87
10.1 Statistical hypotheses 87
10.2 Sample Size 88
10.3 Definitions of analysis populations 90
10.4 Statistical Analysis Methods 90
10.5 Missing data 92
10.6 Subgroup analysis 92
10.7 Sensitivity analysis 92
10.8 Data analysis 92
11 CLINICAL TRIAL MANAGEMENT 94
11.1 Statement 94
11.2 Ethics 94
11.3 Informed consent 94
11.4 Amendment of clinical trial protocol 95
11.5 Protocol deviation 96
11.6 Monitoring 96
11.7 Quality assurance and audit 97
11.8 Intellectual property 97
11.9 Privacy of subjects 97
11.10 Independent monitoring committee 97
12 FINANCE AND INSURANCE 98
13 PUBLICATION AND DATA SHARING POLICY 98
14 APPENDIX 99
14.1 Appendix I: Women of Child-bearing Potential (WOCBP) 99
14.2 Appendix II: Guideline for Adverse Event Grading Criteria in Clinical Trial of Preventive Vaccines--NMPA Standard 100
15 REFERENCES 110

List of Tables

Table 1 Schedule of Visits ... 14
Table 2 Main Results of Challenge-Protection Studies in Mice or Hamsters Immunized with SCTV01C/E Vaccine ... 27
Table 3 Plan for Challenge-Protection Studies in Mice Immunized with SCTV01E-2 Vaccine ... 27
Table 4 Number of Subjects (%) with Adverse Reactions in Clinical Trial SCTV01C-02-1 ... 32
Table 5 Anti-SARS-CoV-2 IgG Antibodies in Study SCTV01C-02-1 ... 33
Table 6 Authentic Virus Neutralizing Antibody Titers of Study SCTV01C-02-1 ... 34
Table 7 Pseudovirus Neutralizing Antibody Titers of Study SCTV01C-02-1 ... 34
Table 8 Number of Subjects (%) with Adverse Reactions in Clinical Trial SCTV01C -01-1 ... 35
Table 9 Number of Subjects (%) with Adverse Reactions in Clinical Trial SCTV01C -01-2 ... 36
Table 10 Number of Subjects (%) with Adverse Reactions in Cohort 1 of Clinical Trial SCTV01C-E-01-UAE-1 ... 38
Table 11 Number of Subjects (%) with Adverse Reactions in Cohort 2 of Clinical Trial SCTV01C-E-01-UAE-1 ... 39
Table 12Authentic Virus Neutralizing Antibody Titers and Seroresponse Rates (%) in UAE Phase III Cohort 1 ... 40
Table 13 Authentic Virus Neutralizing Antibody Titers and Seroresponse Rates (%) in UAE Phase III Cohort 2 ... 40
Table 14 SCTV01E-MRCT-2 TEAE Summary ... 42
Table 15 Solicited Local AEs in SCTV01E-MRCT-2 ... 42
Table 16 Solicited Systemic AEs in SCTV01E-MRCT-2 ... 43
Table 17 Items for Blood Sample Collection and Sampling Volume ... 68
Table 18 List of Solicited AEs ... 72
Table 19 List of AESIs Collected after Vaccination ... 73
Table 20 General Rules for Severity Grading of Other AEs ... 74
Table 21 Severity of COVID-19Disease ... 81
Table 22 Grading of Injection Site (Local) Adverse Events ... 100
Table 23 Grading of Vital Signs ... 102
Table 24 Grading of Non-Injection-Site (Systematic) Adverse Events ... 104
Table 25 Grading of Blood Biochemistry Indicators ... 107
Table 26 Grading of Hematology Indicators ... 108
Table 27 Grading of Urinalysis Indicators ... 109
Table 28 General Rules for Severity Grading of Other Adverse Events ... 109

List of Figures

Figure 1 The Schema of Study Design 4
Figure 2 The Schema of Study Design 51
Figure 3 The Diagnostic Procedures of COVID-19 82

ABBREVIATIONS

Abbreviation	Full name
ACE-2	Angiotensin-converting enzyme 2
ADE	Antibody-dependent enhancement
AE	Adverse events
AESI	Adverse event of special interest
ALT	Alanine aminotransferase
ANCOVA	Analysis of covariance
AST	Aspartate aminotransferase
ADR	Adverse drug reaction
BMI	Body mass index
CDE	Center for Drug Evaluation
CDISC	Clinical Data Interchange Standards Consortium
CI	Confidence interval
COVID-19	Coronavirus Disease 2019
CRO	Contract Research Organization
DBL	Database lock
DM	Data manager
EAC	Endpoint Assessment/Adjudication Committee
eCRF	Electronic Case Report Form
ECD	Extracellular domain
EC	Ethics Committee
EDC	Electronic data capture
EOS	End of study
EUL	Emergency Use Listing
FAS	Full analysis set
GBS	Guillain-Barre syndrome
GCP	Good Clinical Practice
GLP	Good Laboratory Practice
GMC	Geometric mean concentration
GMR	Geometric mean ratio
GMT	Geometric mean titer
H_0	Null hypothesis
hACE2	Human angiotensin-converting enzyme 2
IB	Investigator's Brochure
ICF	Informed consent form
ICH	International Conference on Harmonization of Technical Requirements for Registration of Pharmaceuticals for Human Use
IFN-γ	Interferon-γ
I-FAS	Full analysis set for immunogenicity
IL	Interleukin
I-PPS	Per-protocol set for immunogenicity
IRB	Ethics Review Committee
IP	Investigational product
IWRS	Interactive Web Response System
MedDRA	Medical Dictionary for Regulatory Activities
MERS	Middle East respiratory syndrome
MTD	Maximum tolerated dose
mRNA	Messenger ribonucleic acid

NMPA	National Medical Products Administration
NOAEL	No observed adverse effect level
PCR	Polymerase chain reaction
PPS	Per-protocol set
PT	Preferred term
QC	Quality control
RBD	Receptor-binding domain
SAE	Serious adverse events
SAP	Statistical Analysis Plan
SARS	Acute respiratory syndrome
SAS	Statistical analysis system
SDV	Source data verification
SARS-CoV-2	Severe acute respiratory syndrome coronavirus 2
S-ECD	Extracellular domain of recombinant spike protein of SARS-CoV-2 variant
SOC	System Organ Class
SOP	Standard operating procedure
SRR	Seroresponse rate
SS	Safety set
SUSAR	Suspected unexpected serious adverse reaction
Th1	Helper T cell 1
Th2	Helper T cell 2
TNF-α	Tumor necrosis factor-α
RdRp	RNA-dependent RNA polymerase
RNA	Ribonucleic acid
VAED	Vaccine-associated enhanced disease
VARED	Vaccine-associated enhanced respiratory disease
VED	Vaccine-enhanced disease
VRC	Vaccination record card
VOC	Variant of concern
WHO	World Health Organization
WHO DD	World Health Organization Dictionary of Drugs
2019-nCoV	Severe acute respiratory syndrome coronavirus 2019

1 STUDY BACKGROUND

1.1 BACKGROUND

Coronavirus disease 2019 (COVID-19) is a new acute respiratory infectious disease caused by severe acute respiratory syndrome coronavirus 2 (SARS-CoV-2). COVID-19 was first identified in humans in December 2019 and spread rapidly to more than 210 countries and regions worldwide. The World Health Organization (WHO) declared COVID-19 a “public health emergency of international concern” on January 30, 2020, and announced its outbreak a global pandemic on March 11, 2020. As of June 28, 2023, WHO had published 767,518,723 confirmed cases, including 6,947,192 deaths[1]. The outbreak and epidemic of COVID-19 seriously threaten human health and survival. Vaccines have become the most effective means to prevent viral infection. On March 10, 2023, WHO announced that 183 SARS-CoV-2 vaccines had entered clinical studies worldwide, and 199 were at the pre-clinical research stage[2]. As of October 19, 2022, WHO had included 11 SARS-CoV-2 vaccines in the Emergency Use Listing (EUL)[3]. As of June 30, 2023, 16 SARS-CoV-2 vaccines had been approved for conditional marketing or emergency use in China, including SARS-CoV-2 vaccines with multiple technical routes such as inactivated vaccines, recombinant protein vaccines, adenovirus vector vaccines and mRNA vaccines.

SARS-CoV-2 is a single-stranded RNA virus prone to deletion mutations that mainly occur in the S protein's recurrent deletion regions (RDRs). Deletion or mutation may change the conformation of the S protein, resulting in decreased vaccine immunological efficacy and virus immune escape. With the pandemic of SARS-CoV-2, several high-risk mutant strains have emerged worldwide: Alpha (B.1.1.7), Beta (B.1.351), Gamma (P.1), Delta (B.1.617.2), and Omicron (B.1.1.529). They have been proven to increase viral transmission, exacerbate disease progression (increase hospitalization or mortality), reduce COVID-19 immunity from previous infections or immunizations, decrease the efficacy of treatment or vaccines, and invalidate diagnostic tests. The emerging Omicron (B.1.1.529) variant was first reported to WHO from South Africa on November 24, 2021 and designated as a variant of concern (VOC) on November 26, 2021. Omicron (B.1.1.529), which spreads much faster than other variants, has become the major SARS-CoV-2 variant worldwide. Up to now, 5 subtypes (BA.1, BA.2, BA.3, BA.4, BA.5) of Omicron have evolved into a series of 709 descendant subvariants, including 72 recombinant subvariants. New Omicron subvariants will continue to emerge as SARS-CoV-2 continues to spread worldwide. BA.5.2 had been the most prevalent Omicron variant in the world for several months. However, subvariants BF.7, BQ.1, and BQ.1.1 and recombinant variants (XBB), which are more capable of immune escape and transmission,

have replaced BA.5.2 as the dominant prevalent strains in some countries and regions since October 2022[4].

With the occurrence of SARS-CoV-2 mutations, the protection efficiency of the first-generation vaccines based on the original strain has decreased significantly, especially against Omicron variants[5]. The decreases in the protection efficiency of previous vaccinations have led to the prevalence of Omicron, and there is an urgent need for Omicron-specific vaccines to induce immune responses and protect humans from infection and morbidity.

SCTV01E-2 is a vaccine containing four antigen proteins, i.e., TM23 (Beta), TM41 (Omicorn BA.1), TM41F (Omicorn BQ.1.1), and TM41H (Omicron XBB.1) proteins. Multiple antigens from different variants can induce a broader neutralizing antibody spectrum than a single antigen from a particular variant. SCTV01E-2 is not only a novel vaccine specifically designed to prevent infection with SARS-CoV-2 and its variants but also an affordable, thermostable SARS-CoV-2 vaccine, which has broad immunoprotective efficacy against existing and emerging variants.

1.2 ETIOLOGICAL CHARACTERISTICS

SARS-CoV-2, belonging to the genus β-coronavirus, is an enveloped spherical or oval virus, with a diameter of 60–140 nm. It contains 5 essential genes targeting 4 structural proteins (spike (S), nucleocapsid (N), membrane (M), envelope (E)) and ribonucleic acid (RNA)-dependent RNA polymerase (RdRp). The N protein encapsulates the RNA genome to form a nucleocapsid surrounded by the viral envelope (E), which contains M and S proteins. The S protein enters a cell by binding angiotensin-converting enzyme 2 (ACE-2). When isolated and cultured in vitro, SARS-CoV-2 can be found in human respiratory epithelial cells within about 96 h, whereas it takes approximately 4 to 6 days to be detected in Vero E6 and Huh-7 cell lines[6].

Coronaviruses are sensitive to ultraviolet light and heat, which can be effectively inactivated by treatment at 56°C for 30 min or with lipid solvents such as ether, 75% ethanol, chlorine-containing disinfectants, peroxyacetic acid and chloroform, except chlorhexidine.

1.2.1 CLINICAL MANIFESTATIONS

The clinical manifestations of COVID-19 mainly include dry throat, sore throat, cough, fever (mostly moderate- to low-grade fever and occasionally hyperpyrexia, lasting no more than 3 days), etc. Some patients experience myalgia, loss of taste or smell, nasal obstruction, running nose, diarrhea, conjunctivitis, etc. In a few patients, the disease continues to progress, with persisting fever and occurrence of pneumonia-related manifestations. Critically ill patients

usually develop dyspnea and/or hypoxemia 5 to 7 days after the disease onset. In severe cases, the disease may progress rapidly to acute respiratory distress syndrome, septic shock, uncorrectable metabolic acidosis, bleeding/clotting disorders, and multiple organ failure. Very few patients have central nervous system involvement, etc[7]. As with other respiratory coronaviruses, SARS-CoV-2 mainly spreads via respiratory droplets and close contact and may also spread via aerosols and virus-contaminated articles, with an incubation period of up to 2–4 days[7].

1.2.2 SOURCE OF INFECTION AND ROUTE OF TRANSMISSION

SARS-CoV-2-infected and asymptomatic patients are the main sources of infection with SARS-CoV-2, which is infectious in the incubation period and highly contagious within 5 days after symptom onset. SARS-CoV-2 mainly spreads via respiratory droplets and close contact. Contact with virus-contaminated articles can also cause infection. There is potential for aerosol transmission in the case of prolonged exposure to high concentrations of aerosols in relatively closed environments. Since SARS-CoV-2 can be isolated from feces and urine, attention should be paid to its transmission by contact or aerosol due to environmental contamination[7].

1.3 INTRODUCTION TO THE STUDY VACCINE

SCTV01E-2 is a recombinant protein vaccine developed by Sinocelltech, Ltd. and manufactured using genetic engineering that enables CHO cells to express the virus antigen. It is an oil-in-water adjuvanted vaccine suspension specifically designed to prevent infection with SARS-CoV-2 and its variants, with a strength of 0.5 mL/vial, containing 5 μg of TM23 protein (Beta), 5 μg of TM41 protein (Omicron BA.1), 10 μg of TM41F protein (Omicron BQ.1.1), 10 μg of TM41H protein (Omicron XBB.1), SCT-VA02B adjuvant, and excipients such as sodium citrate and sodium chloride, without preservatives and antibiotics. The composition, formulation, process and significant quality controls (e.g. particle size) of SCT-VA02B are the same as those of the marketed MF59 adjuvant. MF59 is superior to aluminium adjuvant in inducing T helper (Th)1 immune response in humans, as aluminium adjuvant mainly induces Th2 responses. The SCTV01E-2 trimeric subunit protein consists of the ECD of the S protein (S-ECD, including S1 and S2) and T4-Foldon. The S-ECD-mimicked S protein has complete biological functions and natural structural properties, which ensures the high proportion of correct structural neutralizing epitopes and the induction of high-titer neutralizing antibodies. It has more T cell epitopes and induces stronger resistance to variant infection.

Sinocelltech, Ltd. has developed a series of SARS-CoV-2 variant vaccines, including SCTV01C, SCTV01E and SCTV01E-2, which all are recombinant protein vaccines against SARS-CoV-2 variants independently developed using genetic engineering on the same CHO

cell production platform. SCTV01C and SCTV01E have been approved for emergency use in China. Compared with SCTV01C and SCTV01E, SCTV01E-2 is likewise designed with trimeric proteins, produced using the same CHO cell platform and has highly similar quality (high purity, low impurity content). fourCurrently, four clinical studies of SCTV01E have been conducted at home and abroad. The safety data from approximately 6000 subjects show no safety signal for SCTV01E. In a phase III immunogenicity study, SCTV01E demonstrated superior immunogenicity to the Pfizer mRNA COVID-19 vaccine. These studies provide adequate safety and immunogenicity data to support the clinical use of SCTV01E-2.

1.4 INTRODUCTION TO PRE-CLINICAL STUDIES

1.4.1 NON-CLINICAL PHARMACOLOGY

1.4.1.1 In vitro pharmacodynamics

The S-trimer subunit protein of SCTV01E-2 consists of the ECD of the S protein (S-ECD, including S1 and S2) and T4-Foldon. The Furin cleavage site between S1 and S2 is removed from the trimeric S-ECD protein to prevent instability due to digestion. Trimeric proteins TM23, TM41, TM41F, and TM41H all bind ACE2 with high affinity. The affinity of TM23, TM41, and TM41F for ACE2 is 0.68, 1.5, and 3.0 nM, respectively, higher than the affinity of the S-trimer protein of the D614G strain for ACE2 (6.0 nM). The affinity of TM41H for ACE2 is similar to that of D614G. The higher receptor binding affinity may partially explain the increased transmission capacity of the Omicron variant. The trimeric S-ECD protein has a structural basis for protection against viral infection as it retains natural structural properties, has complete biological functions and correct conformational characteristics, and can induce effective neutralizing antibodies against multiple epitopes[8].

1.4.1.2 In vivo pharmacodynamics

Immunogenicity study

1. Comparative study of neutralizing activity between SCTV01E-2 and SCTV01E

(1) In mice immunized with three prime doses, i.e. D614G × 2 + BA.5 × 1 (to simulate the population with BA.5 breakthrough infections after prime immunizations with original strain vaccines), a single booster dose of SCTV01E-2 induced a high level of humoral immune response. SCTV01E-2 induced relatively broad-spectrum neutralizing activity against current predominant vaccine strains (BQ.1.1, XBB.1, XB.1, XBB.1.5, XB.16), early predominant variant BA.5, and new mutant non-epidemic variants (CH.1.1, CA.3.1, CM.8.1.1) and was superior to SCTV01E in enhancing neutralizing activity against multiple variants including current predominant vaccine strains (BA.5, BQ.1.1, XBB.1, XBB.1.5, XBB.1.16, CH.1.1, CA.3.1, CM.8.1.1).

Moreover, SCTV01E-2 was superior to SCTV01E in inducing neutralizing activity against authentic XBB.1 variant, and there was a significant positive correlation between the pseudovirus neutralizing titer (PNT_{50}) and authentic virus neutralizing titer (MNT_{50}).

(2) In mice primed with two doses of D614G (to simulate the population without breakthrough infections after prime immunizations with original strain vaccines), a single booster dose of SCTV01E-2 was superior to a single booster dose of SCTV01E in inducing neutralizing activity against current predominant vaccine strains (BQ.1.1, XBB.1); two booster doses of SCTV01E-2 could further elevate the pseudovirus neutralizing titers against various variants including present predominant vaccine strains (BA.4/5, BQ.1.1, XBB.1.5, CH.1.1, CA.3.1, CM.8.1.1), which was superior to booster immunizations with SCTV01E.

(3) In Naïve C57BL/6J mice and SD rats, two prime doses of SCTV01E-2 were superior to two prime doses of SCTV01E in inducing neutralizing activity against current predominant vaccine strains Omicron BQ.1.1, XBB.1, XBB.1.5, and XBB.1.16.

2. Comparability study of immunogenicity strength of SCTV01E-2 and SCTV01E

In Naïve C57BL/6J mice and SD rats, two prime doses of SCTV01E-2 and SCTV01E induced comparable antigen-specific IgG antibody titers and Th1/Th2 immune responses.

Challenge-protection efficacy

SCTV01E-2 is a vaccine modified from SCTV01E through antigen iteration, with TM23 and TM41 proteins designed based on Beta and BA.1 variant antigens retained and TM41F and TM41H proteins designed based on BQ.1.1 and XBB.1 antigens added. Protective efficacy studies of SCTV01C and SCTV01E have been conducted in different animal challenge models. The results showed that they had good protective efficacy against current prevalent new variants in challenge models, and no antibody-dependent enhancement (ADE) or vaccine-enhanced disease (VED) was observed (**Table 2**). Immunogenicity studies showed that compared to SCTV01E, SCTV01E-2 retained high neutralizing potency against Beta and BA.1 variants but had significantly increased neutralizing potency against BQ.1.1 and XBB.1. The S-ECDs of Beta and Omicron variants share > 94% amino acid homology, so SCTV01E-2 and SCTV01E will induce similar T-cell responses. Considering that SCTV01E-2 increases broad-spectrum neutralizing activity based on maintaining T-cell immune responses, theoretically, it can effectively resist pathological changes caused by infection with multiple variants and can effectively protect animals against viral hazards. Subsequent challenge-protection studies of SCTV01E-2 against BA.5.2 or BF.7 (depending on the strain from the challenge partner) will be conducted in a mouse model (**Table 3**).

The main results of challenge-protection studies in mice or hamsters immunized with

SCTV01C/E vaccine are as follows:

Table 2 Main Results of Challenge-Protection Studies in Mice or Hamsters Immunized with SCTV01C/E Vaccine

Species	Vaccine	Immunization dose	Immunization interval	Challenge time	Challenge variant	Decrease in lung viral load (gRNA)	Decrease in lung pathology score
hACE2-KI/NIFDC mice	Monovalent TM23 vaccine	10 μg	Two doses-0/21	14 days after the second dose	Original strain	2.85 log****	1.5 points**
					Beta strain	4.38 log****	1.2 points*
	SCTV01C	6 μg/dose	Two doses-0/21	14 days after the second dose	Beta strain	5.51 log****	2.0 points**
Hamsters	SCTV01C	6 μg/dose	Two doses-0/21	26 days after the second dose	Original strain	3.5 log*	2.0 points*
	SCTV01E	6 μg/dose	Two doses-0/21	15 days after the second dose	BA.2	2.31 log***	1.0 point[#]

Table 3 Plan for Challenge-Protection Studies in Mice Immunized with SCTV01E-2 Vaccine

Species	Immunogen	Dosage	Immunization regimen	Number of animals	Immunogenicity endpoints	Virus type	Post-challenge indicator testing
K18-hACE2 ♀	Blank control	/	D0, D21, 2 doses-intramuscular injection	6 animals	Testing on D28: immunogen-specific antibody titers; Beta, BA.1, BA.5, BQ.1.1, and XBB.1 pseudovirus neutralizing titers	BA.5.2 or BF.7 variant, 15 days after the second dose, 4 × 10^5 PFU Intranasal (3 animals/group)	Testing 4 dpi. (4 days post-infection, n = 3/group): lung viral gRNA load, lung histopathology
	SCTV01E-2 & SCTVA-02B	1 μg/dose & 2 mg (adjuvant)		6 animals			

1.4.2 TOXICOLOGY

SCTV01E-2 vaccine contains four active components of recombinant S extracellular domain (S-ECD) trimer, namely TM23 (Beta), TM41 (Omicron BA.1), TM41F (Omicron BQ.1.1), and TM41H (Omicron XBB.1) proteins, with only a few mutation site differences and high homology among the molecules. The previous generation, quadrivalent SCTV01E vaccine (Alpha, Beta, Delta, Omicron BA.1) has been authorized for national emergency use.

A complete set of toxicology studies and several clinical studies have shown good safety of this vaccine. SCTV01E-2 is a modified vaccine based on SCTV01E, with new epidemic antigenic subtypes adjusted but total antigen amount unchanged, which is manufactured using the same platform process and quality control. According to the *Guidelines for the Development and Evaluation of Prophylactic Vaccines against SARS-CoV-2 Variants* (Trial) issued by CDE[9], modified vaccines developed based on the first-generation vaccines (phase II studies have been completed) generally do not require other nonclinical safety studies other than irritation and allergy tests of the final product, so SCTV01E-2 has undergone irritation and allergy tests. Based on the favourable safety of SCTV01E, the similarity of the antigens, and the same clinical dose, no clinical safety risk is expected for SCTV01E-2.

The nonclinical toxicology studies of SCTV01E and SCTV-1E-2 were conducted in strict compliance with *Good Laboratory Practice* (GLP), including single-dose toxicity, 6-week repeat-dose toxicity (along with safety pharmacology, immunogenicity, immunotoxicity, and local tolerance), and reproductive and developmental toxicity studies of SCTV01E and local irritation and allergy tests of SCTV01E-2. The comprehensive safety evaluation could support product marketing and subsequent development of modified vaccines.

1.4.2.1 Single-dose toxicity study (SCTV01E)

No mortality or moribundity was observed in SD rats within 14 days after a single intramuscular injection of SCTV01E at 4 doses/animal. There were no abnormal clinical observations but only slow body weight gain and decreased food consumption possibly related to acute phase responses. No abnormal changes were observed at gross necropsy. The maximum tolerated dose (MTD) was ≥ 4 doses (120 μg)/animal in rats.

1.4.2.2 Repeat-dose toxicity study (SCTV01E)

SCTV01E was repeatedly administered to SD rats by intramuscular injection at 1 or 3 doses (30 or 90 μg)/animal, once every two weeks for six consecutive weeks (4 times in total), followed by a 2-week recovery period. Animals in the low- and/or high-dose SCTV01E groups showed increased Neut, Eos, FIB, and Glb, decreased Retic and Alb, as well as increased numbers of red pulp cells in the spleen and plasma cells in inguinal lymph nodes, which might be related to immune or acute phase responses. Glomerulonephritis that might be caused by high antibody titers was seen in 2/20 animals in the 3 doses/animal group. After the 2-week recovery period, all the above changes were recovered or showed a recovery trend, no obvious systemic toxicity was observed, and the no observed adverse effect level (NOAEL) was considered to be 1 dose/animal.

1.4.2.3 Immunogenicity study (SCTV01E)

Repeated intramuscular injection of SCTV01E at 1 dose (30 μg)/animal and 3 doses (90

μg)/animal for 6 weeks induced robust immune responses in SD rats. The titers of specific antibodies against the antigens of the four variants increased with the number of doses and tended to be saturated after D14; the peak GMT against each variant antigen in the low- and high-dose groups was distributed before the third dose or at 1 week of recovery. The neutralizing antibody detection results showed that the titers of neutralizing antibodies against the four variant pseudoviruses increased with the number of doses; GMT peaked at 1 week of recovery in the high-dose group and for all the other variants in the low-dose group, except for the antibody titer against Omicron in the low-dose group, which peaked before the third dose.

When SCTV01E was administered to SD rats at 1 dose (30 μg)/animal and 3 doses (90 μg)/animal, specific IgG antibodies and neutralizing antibodies against SARS-CoV-2 variants Alpha (B.1.1.7), Beta (B.1.351), Delta (B.1.617.2), and Omicron BA.1 (B.1.529) were detected in the serum of dams, fetuses, and F1 pups. Antibodies in dams could be transported in large amounts through milk to pups, but not in large amounts through the blood-fetal barrier to fetuses.

1.4.2.4 Immunotoxicity study (SCTV01E)

It was concomitantly found in the repeat-dose toxicity study that rats in the placebo group and/or low- and high-dose SCTV01E groups showed decreased Alb, increased Glb, Neut and Eos, increased spleen organ weight/organ-to-body weight ratio/organ-to-brain weight ratio, as well as histopathological changes including an increased count of red pulp cells in the spleen and increased plasma cells in inguinal lymph nodes, on Day 3 after the last dose (D46). The above changes were considered to be related to the immune response or acute phase response after administration. In addition, decreased CD3+ was observed in peripheral blood T-lymphocyte subsets in the low- and high-dose test article groups, and increased cytokines TNF-α, IFN-γ, IL-2, and IL-6 were observed in some of the animals, which were considered to be possibly related to the acute phase response, local irritation at the injection site, and/or immune response. The gross necropsy observation of lymphatic organs/tissues, the thymus organ weight, and the histological examination of organ tissues such as thymus and bone marrow showed no abnormal changes related to drug administration. At the end of the 2-week recovery period (D57), the above changes recovered or tended to recover. In summary, when SCTV01E was administered to SD rats by repeated intramuscular injections at doses of 1 dose/animal and 3 doses/animal for 6 weeks (4 times in total), reversible immune response-related changes were observed, with no immunotoxicity reaction.

1.4.2.5 Reproductive and developmental toxicity study (SCTV01E)

SCTV01E was repeatedly administered to SD rats by intramuscular injection at the dosage

of 1 dose (30 µg)/animal and 3 doses (90 µg)/animal. Male rats were dosed 4 times before mating, separately at Week 0 (D1), Week 2 (D15), Week 4 (D29), and Week 6 (D43), while female rats were dosed three times before mating, separately at Week 0 (D1), Week 2 (D15), and Week 4 (D29). Female rats and male rats were mated in the same cage on D50-male/D36-female. Female rats used for necropsy on gestation day 20 (GD20) were additionally dosed on GD6 (4 times in total). Female rats used for delivery were dosed once each on GD6 and lactation day 7 (LD7) (5 times in total). In addition to placebo-related changes at the administration site and transient slight decreases in body weight and food consumption, no obvious toxic response was observed in parental female and male rats, pregnant/lactating female rats, and embryonic and fetal development in each dose group. The no observed adverse effect level (NOAEL) was 3 doses/animal for fertility of parental female and male rats, the NOAEL for embryo-fetal development was 3 doses/animal (no fetal teratogenic toxicity was observed in each dose group), and the NOAEL for survival, growth and development of F1 generation animals was 3 doses/animal.

1.4.2.6 Allergy test (SCTV01E-2)

When SCTV01E-2 was intramuscularly injected at 0.1 dose (3 µg)/animal and 1 dose (30 µg)/animal for sensitization and intravenously injected at 0.2 dose (6 µg)/animal and 2 doses (60 µg)/animal for challenge, no allergic reactions were observed in animals sensitized at clinical doses after challenge.

1.4.2.7 Local tolerance test

The local irritation study of SCTV01E concomitantly performed in the repeat-dose toxicity study in SD rats showed that mild swelling at the administration site was observed 1–3 days after the last dose and then fully recovered in the placebo group and low- and high-dose SCTV01E groups; injection site swelling was observed at gross necropsy 3 days after the last dose (D46), with histopathology manifested as slight to moderate granulomatous inflammation. In addition, inflammatory cell infiltration around the sciatic nerve and subcutaneous granulomatous inflammation in the skin (around mammary glands) caused by the spread of inflammation at the injection site were considered irritation reactions caused by adjuvants, which showed recovery trends after 2 weeks of drug withdrawal.

The local irritation study of SCTV01E-2 in New Zealand rabbits showed that repeated intramuscular injection of SCTV01E-2 at 1 dose/animal once weekly for a total of 3 times resulted in no obvious abnormal injection site observations and gross necropsy observations. Histopathological examination revealed local irritation reactions (interstitial mixed inflammatory cell/neutrophil infiltration, fibrin exudation, and muscle fiber mineralization) at the injection site, which showed recovery trends after 2 weeks of drug withdrawal.

1.4.2.8 Other experiments

According to the International Conference on Harmonization (ICH) guidelines and the *General Principles for Technical Review of Preclinical Safety Evaluation of Preventive Biological Products* issued by the National Medical Products Administration (NMPA) in 2008[11], vaccines usually do not require genotoxicity studies, carcinogenicity studies, dependence studies and conventional pharmacokinetic studies. In general, recombinant protein vaccines proposed to be injected intramuscularly in clinical practice also do not require hemolysis studies. SCTV01E and SCTV01E-2 are SARS-CoV-2 recombinant protein vaccines. Thus, no other studies have been conducted according to the above regulatory requirements.

1.5 CLINICAL SAFETY AND IMMUNOGENICITY OF SCTV01E AND SCTV01C

SCTV01E-2 and SCTV01C/E have very similar molecular properties. SCT has initiated several clinical trials of SCTV01C and SCTV01E, of which 3 phase I/II clinical trials of SCTV01C and 1 phase III clinical trial of SCTV01C and SCTV01E have completed interim analysis, and preliminary safety and immunogenicity data have been obtained. One phase II clinical trial of SCTV01E and one phase III protective efficacy clinical trial of SCTV01E in China have also been initiated and are in the enrollment and follow-up phases. Since SCTV01E-2 does not have any clinical study data, this section will present the clinical study data from SCTV01C and SCTV01E to support the conduct of this clinical study of SCTV01E-2.

The first to be introduced is a randomized, double-blind, placebo-controlled phase I/II study in primed Chinese populations (SCTV01C-02-1), which preliminarily evaluated the safety and immunogenicity of SCTV01C in people aged 18 years and above who had not received a SARS-CoV-2 vaccine. The second and third are randomized, double-blind, placebo-controlled phase I/II booster studies in UAE (SCTV01C-01-1 and SCTV01C-01-2), which evaluated the safety and immunogenicity of SCTV01C in people aged 18 years and above who had previously received inactivated and mRNA vaccines, respectively. The fourth is a phase III clinical trial of SCTV01E and SCTV01C boosters (SCTV01C-E-01-UAE-1) conducted in UAE to evaluate the safety and immunogenicity of SCTV01E and SCTV01C in people aged 18 years and above.

1.5.1 DOMESTIC PHASE I/II CLINICAL TRIAL: PRIMED POPULATION STUDY (SCTV01C-02-1)

1.5.1.1 Safety results of study SCTV01C-02-1

This is a phase I/II clinical study in subjects' ≥ 18 years of age who had not previously received a SARS-CoV-2 vaccine. As of August 31, 2022, a total of 478 subjects were enrolled,

of whom 356 were vaccinated with SCTV01C (279 subjects aged 18–59 years, 77 subjects aged ≥ 60 years), 61 with adjuvant, and 61 with normal saline. The results showed favorable safety of SCTV01C (comparable in the 20 μg and 40 μg groups, better in the elderly group than in the adult group). No SAEs occurred in the test group, most of the adverse reactions were Grade 1–2, and seven Grade ≥3 adverse reactions (fever, fatigue, cold and heat intolerance, headache, systemic muscle pain, upper respiratory tract infection) occurred in 4 subjects (1.1%). The solicited adverse reactions (≥ 3%) were injection site pain (20.8%, 74/356), fever (7.6%, 27/356), injection site swelling (4.5%, 16/356), and fatigue (3.7%, 13/356); none of the unsolicited adverse reactions exceeded 3%. See Table 4 for details.

Table 4 Number of Subjects (%) with Adverse Reactions in Clinical Trial SCTV01C-02-1

			SCTV01C			
**Preferred term**	**Placebo (N = 61) n (%)**	**Adjuvant (N = 61) n (%)**	**18–59 years 20 μg (N = 139) n (%)**	**18–59 years 40 μg (N = 140) n (%)**	**≥ 60 years 20 μg (N = 77) n (%)**	**≥ 18 years Total (N = 356) n (%)**
Overall adverse reactions	9 (14.8)	17 (27.9)	54 (38.8)	49 (35.0)	17 (22.1)	120 (33.7)
**Solicited ARs**	6 (9.8)	12 (19.7)	46 (33.1)	41 (29.3)	7 (9.1)	94 (26.4)
Solicited local ARs	2 (3.3)	10 (16.4)	43 (30.9)	32 (22.9)	3 (3.9)	78 (21.9)
Injection site pain	2 (3.3)	10 (16.4)	41 (29.5)	31 (22.1)	2 (2.6)	74 (20.8)
Injection site swelling	0	3 (4.9)	6 (4.3)	10 (7.1)	0	16 (4.5)
Injection site erythema	0	2 (3.3)	7 (5.0)	1 (0.7)	0	8 (2.2)
Injection site induration	0	1 (1.6)	5 (3.6)	2 (1.4)	0	7 (2.0)
Injection site pruritus	0	1 (1.6)	3 (2.2)	2 (1.4)	1 (1.3)	6 (1.7)
Solicited systemic ARs	4 (6.6)	4 (6.6)	21 (15.1)	24 (17.1)	5 (6.5)	50 (14.0)
Fever	3 (4.9)	2 (3.3)	11 (7.9)	12 (8.6)	4 (5.2)	27 (7.6)
Fatigue	0	1 (1.6)	4 (2.9)	9 (6.4)	0	13 (3.7)
Drowsiness	0	2 (3.3)	6 (4.3)	3 (2.1)	0	9 (2.5)
Headache	0	1 (1.6)	3 (2.2)	4 (2.9)	0	7 (2.0)
Diarrhea	0	1 (1.6)	2 (1.4)	3 (2.1)	1 (1.3)	6 (1.7)
Nausea	0	0	2 (1.4)	3 (2.1)	0	5 (1.4)
Constipation	1 (1.6)	0	0	0	0	0
Myalgia	0	1 (1.6)	2 (1.4)	4 (2.9)	1 (1.3)	7 (2.0)
Joint pain	0	0	2 (1.4)	1 (0.7)	0	3 (0.8)
Arthritis	0	0	0	1 (0.7)	0	1 (0.3)
Pruritus	1 (1.6)	0	1 (0.7)	1 (0.7)	0	2 (0.6)
Anxiety	0	0	2 (1.4)	0	0	2 (0.6)
Insomnia	0	1 (1.6)	0	0	0	0
Cough	0	0	0	1 (0.7)	0	1 (0.3)
**Unsolicited ARs**	4 (6.6)	6 (9.8)	15 (10.8)	17 (12.1)	11 (14.3)	43 (12.1)

1.5.1.2 Immunogenicity results of study SCTV01C-02-1

Anti-SARS-CoV-2 IgG antibody

Two prime doses of SCTV01C (0- and 28-day immunization procedures) induced significantly high levels of total IgG antibodies against SARS-CoV-2 Alpha, Beta, and Delta variants (up to 4884-fold), which remained high (approximately 500-fold) 90 days after two

doses of vaccination, with p-values of < 0.0001 compared to the adjuvant group; there was no significant difference in the immunogenicity of SCTV01C between different age and dose groups.

Total anti-Alpha variant IgG antibodies: GMT increased 153-fold, 4884-fold, 2384-fold, and 506-fold from baseline on D28, D42 (phase I study only), D56, and D118 after SCTV01C inoculation. Total anti-Beta variant IgG antibodies: GMT increased 199-fold, 3304-fold, 2001-fold, and 507-fold from baseline on D28, D42 (phase I study only), D56, and D118 after SCTV01C inoculation. Total anti-Delta variant IgG antibodies: GMT increased 182-fold, 2538-fold, 1544-fold, and 499-fold from baseline on D28, D42 (phase I study only), D56, and D118 after SCTV01C inoculation. See **Table 5** for details.

Table 5 Anti-SARS-CoV-2 IgG Antibodies in Study SCTV01C-02-1

SCTV01C	Anti-Alpha IgG GMT (95% CI)	Anti-Beta IgG GMT (95% CI)	Anti-Delta IgG GMT (95% CI)
**Baseline**	31 (28, 34)	31 (28, 34)	30 (25, 36)
**D28**	4717 (4005, 5554)	5825 (4979, 6814)	5425 (3712, 7927)
**D42**	147501 (120913, 179936)	95012 (77683, 116206)	75326 (59999, 94569)
**D56**	71195 (61864, 81933)	58400 (52114, 65443)	46133 (37792, 56315)
**D118**	16746 (14919, 18797)	16805 (15231, 18543)	13362 (10077, 17718)

Authentic virus-based neutralization assay

Two prime doses of SCTV01C (0- and 28-day immunization procedures) induced significantly high levels of neutralizing antibodies against authentic Alpha, Beta, and Delta variants, with peak GMTs at 14 days after two doses, 100% seroconversion in each SCTV01 dose group, and high levels of neutralizing antibodies after 90 days. The titers of neutralizing antibodies induced by SCTV01C against the Omicron (BA.2) variant were slightly lower than those against other variants, but the neutralizing antibody levels were maintained until 90 days after two doses and 9-fold higher than the baseline GMT, with p-values of < 0.0001 compared to the adjuvant group. The titers of neutralizing antibodies against each SARS-CoV-2 variant generated after two doses of 20 μg of SCTV01C were slightly lower in the ⩾ 60-year-old group than in subjects aged 18–59 years, but there was no significant difference in the immunogenicity of SCTV01C between dose groups.

Authentic virus-based neutralization assay against Alpha variant: GMT increased 270-fold, 120-fold, and 64-fold from baseline on D42 (phase I study only), D56, and D118 after SCTV01C inoculation. Authentic virus-based neutralization assay against Beta variant: GMT increased 254-fold, 111-fold, and 69-fold from baseline on D42 (phase I study only), D56, and D118 after SCTV01C inoculation. Authentic virus-based neutralization assay against Delta variant: GMT increased 107-fold, 53-fold, and 36-fold from baseline on D42 (phase I study only), D56, and D118 after SCTV01C inoculation. Authentic virus-based neutralization assay

against Omicron (BA.2) variant: GMT increased 19-fold, 12-fold, and 9-fold from baseline on D42 (phase I study only), D56, and D118 after SCTV01C inoculation. See **Table 6** for details.

Table 6 Authentic Virus Neutralizing Antibody Titers of Study SCTV01C-02-1

**SCTV01C**	**Anti-Alpha** neutralizing antibody GMT (95% CI)	**Anti-Beta** neutralizing antibody GMT (95% CI)	**Anti-Delta** neutralizing antibody GMT (95% CI)	**Anti-Omicron (BA.2)** neutralizing antibody GMT (95% CI)
**Baseline**	4 (4, 4)	4 (4, 4)	4 (4, 4)	4 (4, 4)
**D42**	1083 (902, 1300)	1018 (805, 1287)	428 (356, 514)	76 (61, 95)
**D56**	481 (408, 567)	446 (389, 512)	214 (169, 270)	48 (41, 57)
**D118**	258 (215, 310)	279 (248, 314)	144 (89, 233)	39 (33, 46)

Pseudovirus-based neutralization assay

Two prime doses of SCTV01C (0- and 28-day immunization procedures) induced significantly high levels of pseudovirus neutralizing antibodies against SARS-CoV-2 Alpha and Beta variants, with *p*-values of < 0.0001 compared to the adjuvant group. There were no significant differences in the immunogenicity of SCTV01C between age and dose groups.

Pseudovirus neutralizing antibody against Alpha variant: GMT increased 11-fold, 228-fold, and 154-fold from baseline on D28, D42 (phase I study only), and D56 after SCTV01C inoculation. Pseudovirus neutralizing antibody against Beta variant: GMT increased 7-fold, 193-fold, and 136-fold from baseline on D28, D42 (phase I study only), and D56 after SCTV01C inoculation. See **Table 7** for details.

Table 7 Pseudovirus Neutralizing Antibody Titers of Study SCTV01C-02-1

**SCTV01C**	**Anti-Alpha** neutralizing antibody GMT (95% CI)	**Anti-Beta** neutralizing antibody GMT (95% CI)
**Baseline**	15 (15, 16)	16 (15, 16)
**D28**	177 (120,261)	123 (83,182)
**D42**	3528 (2922.4259)	3004 (2456.3673)
**D56**	2393 (1925.2974)	2078 (1623.2662)

T-cell subsets

The results of T-cell subset analysis showed that SCTV01C induced a strong cellular immune effect 7 days after the second dose, with significant differences compared to the adjuvant group (all *p*-values < 0.0001). The mean (95% CI) of IL-4 (characterizing Th2) activated T-cell subsets was 8 (3, 14) prior to the second dose (D28) and 380 (200, 560) 7 days after the second dose (D35). The mean (95% CI) of IFN-γ (characterizing Th1) activated T-cell subsets was 17 (5, 28) prior to the second dose (D28) and 447 (203, 692) 7 days after the second dose (D35).

1.5.2 PHASE I/II CLINICAL TRIAL IN UAE: BOOSTER IMMUNIZATION STUDY (SCTV01C-01-1)

1.5.2.1 Safety results of study SCTV01C-01-1

This is a phase I/II clinical study of 1 booster dose of SCTV01C vaccine in subjects' ≥ 18 years of age who had received 2 prior doses of inactivated COVID-19 vaccines. As of September 14, 2022, all 234 subjects had been vaccinated and completed the Day 90 visit, including 159 subjects in the vaccine group and 75 subjects in the normal saline group. The results showed favorable safety of 1 booster dose of SCTV01C on the basis of 2 doses of inactivated vaccines (comparable in the 20 μg and 40 μg groups). No SAEs occurred in the test group, most of the adverse reactions were Grade 1–2, and seven Grade ≥3 adverse reactions occurred (4 fever, 2 increased blood creatine phosphokinase, and 2 increased blood fibrinogen). The solicited adverse reactions (≥ 3%) were injection site pain (11.9%, 19/159) and fever (6.3%, 10/159); none of the unsolicited adverse reactions exceeded 3%. See **Table 8** for details.

Table 8 Number of Subjects (%) with Adverse Reactions in Clinical Trial SCTV01C -01-1

		SCTV01C		
**Preferred term**	**Normal saline (N = 75) n (%)**	**20 μg (N = 79) n (%)**	**40 μg (N = 80) n (%)**	**Total (N = 159) n (%)**
Overall adverse reactions	20 (26.7)	23 (29.1)	23 (28.8)	46 (28.9)
**Solicited ARs**	12 (16.0)	18 (22.8)	14 (17.5)	32 (20.1)
Solicited local ARs	1 (1.3)	13 (16.5)	9 (11.3)	22 (13.8)
Injection site pain	1 (1.3)	10 (12.7)	9 (11.3)	19 (11.9)
Injection site pruritus	0	2 (2.5)	1 (1.3)	3 (1.9)
Injection site swelling	0	2(2.5)	0	2 (1.3)
Injection site erythema	0	1 (1.3)	0	1 (0.6)
Solicited systemic ARs	11 (14.7)	7 (8.9)	5 (6.3)	12 (7.5)
Fever	6 (8.0)	6 (7.6)	4 (5.0)	10 (6.3)
Headache	4 (5.3)	1 (1.3)	1 (1.3)	2 (1.3)
Myalgia	2 (2.7)	1 (1.3)	0	1 (0.6)
Fatigue	0	1 (1.3)	0	1 (0.6)
Pruritus	1 (1.3)	0	0	0
Unsolicited ARs	9 (12.0)	10 (12.7)	14 (17.5)	24 (15.1)

1.5.2.2 Immunogenicity results of study SCTV01C-01-1

The GMCs of WT SARS-CoV-2-specific IgG significantly increased from baseline to 4886 BAU/mL (13.05-fold) and 5852 BAU/mL (12.19-fold) on Day 28 after booster immunizations with 20 and 40 μg of SCTV01C, respectively. The GMC of total specific IgG against WT SARS-CoV-2 also increased significantly from baseline on Day 90 in the 20 μg SCTV01C group (7.50-fold [2757 BAU/mL]) and 40 μg SCTV01C group (4.79-fold [2450 BAU/mL]). On Day 90, the change from Day 28 was 0.57-fold and 0.41-fold in the 20 μg SCTV01C group and 40 μg SCTV01C group, respectively (both p-values < 0.0001).

In the PRNT50 for neutralizing antibodies against authentic SARS-CoV-2 on Day 28 after booster immunization, the GMTs of neutralizing antibodies against Omicron (BA.1) and Delta

variants increased from baseline to 840 (12.19-fold) and 3830 (12.90-fold), respectively, in the 20 μg SCTV01C group; the GMTs of neutralizing antibodies against Omicron (BA.1) and Delta variants increased from baseline to 901 (11.06-fold) and 3935 (11.47-fold), respectively, in the 40 μg SCTV01C group. Similar increases in GMT were also observed on Day 90 in the 20 μg and 40 μg SCTV01C groups. Specifically, the GMTs of neutralizing antibodies against Omicron (BA.1) and Delta variants increased from baseline to 564 (8.24-fold) and 2964 (10.21-fold), respectively, in the 20 μg SCTV01C group; the GMTs of neutralizing antibodies against Omicron (BA.1) and Delta variants increased from baseline to 518 (6.10-fold) and 2224 (6.10-fold), respectively, in the 40 μg SCTV01C group. On Day 90, the fold change in Omicron (BA.1) relative to Day 28 was 0.68 and 0.56 in the 20 μg and 40 μg SCTV01C groups, respectively, whereas the fold change in Delta relative to Day 28 was 0.78 and 0.58 in the 20 μg and 40 μg SCTV01C groups, respectively (all p-values < 0.0001).

ELISpot assay to detect the percentages of IFN-γ and IL-4 positive cells on Day 14 after booster immunization with SCTV01C demonstrated that compared to booster immunization with placebo (normal saline), SCTV01C effectively induced both Th1 cell responses (6.0-fold increase in the 20 μg SCTV01C group and 4.7-fold increase in the 40 μg SCTV01C group) and Th2 cell responses (10.2-fold increase in the 20 μg SCTV01C group and 2.7-fold increase in the 40 μg SCTV01C group).

1.5.3 PHASE I/II CLINICAL TRIAL IN UAE: BOOSTER IMMUNIZATION STUDY (SCTV01C-01-2)

1.5.3.1 Safety results of study SCTV01C-01-2

This is a phase I/II clinical study of 1 booster dose of SCTV01C vaccine in subjects ≥ 18 years of age who had received mRNA COVID-19 vaccines. As of September 14, 2022, all 234 subjects had been enrolled and completed the Day 90 visit, including 159 subjects in the vaccine group and 75 subjects in the normal saline group. The results showed favorable safety of 1 booster dose of SCTV01C on the basis of 2 doses of mRNA vaccines (comparable in the 20 μg and 40 μg groups), with no SAEs but Grade 1–2 adverse reactions. The solicited adverse reactions (≥ 3%) were injection site pain (10.1%, 16/159) and fever (6.3%, 10/159); none of the unsolicited adverse reactions exceeded 3%. See **Table 9** for details.

Table 9 Number of Subjects (%) with Adverse Reactions in Clinical Trial SCTV01C -01-2

		SCTV01C		
**Preferred term**	**Normal saline (N = 75) n (%)**	**20 μg (N = 79) n (%)**	**40 μg (N = 80) n (%)**	**Total (N = 159) n (%)**
Overall adverse reactions	22 (29.3)	30 (38.0)	23 (28.8)	53 (33.3)
**Solicited ARs**	9 (12.0)	18 (22.8)	12 (15.0)	30 (18.9)

		SCTV01C		
**Preferred term**	**Normal saline (N = 75) n (%)**	**20 μg (N = 79) n (%)**	**40 μg (N = 80) n (%)**	**Total (N = 159) n (%)**
Solicited local ARs	2 (2.7)	10 (12.7)	8 (10.0)	18 (11.3)
Injection site pain	2 (2.7)	9 (11.4)	7 (8.8)	16 (10.1)
Injection site pruritus	0	1 (1.3)	1 (1.3)	2 (1.3)
Injection site swelling	0	0	2 (2.5)	2 (1.3)
Injection site erythema	0	0	1 (1.3)	1 (0.6)
Injection site induration	0	0	1 (1.3)	1 (0.6)
Solicited systemic ARs	7 (9.3)	10 (12.7)	5 (6.3)	15 (9.4)
Fever	5 (6.7)	6 (7.6)	4 (5.0)	10 (6.3)
Headache	1 (1.3)	2 (2.5)	0	2 (1.3)
Cough	0	1 (1.3)	1 (1.3)	2 (1.3)
Fatigue	1 (1.3)	1 (1.3)	0	1 (0.6)
**Unsolicited ARs**	14 (18.7)	15 (19.0)	13 (16.3)	28 (17.6)

1.5.3.2 Immunogenicity results of study SCTV01C-01-2

The GMCs of WT SARS-CoV-2-specific IgG significantly increased 2.8-fold and 3.43-fold from baseline on Day 28 after booster immunizations with 20 and 40 μg of SCTV01C, respectively. On Day 90, the GMC of WT SARS-CoV-2-specific IgG slightly increased from Day 28 and increased 0.75- and 0.60-fold from baseline.

On Day 28 after booster immunization, the GMTs of anti-Omicron (BA.1) neutralizing antibodies were 218 (164, 290), 634 (514, 783), and 1083 (868, 1352) in the normal saline, 20 μg SCTV01C, and 40 μg SCTV01C groups, respectively. The GMTs on Day 90 were 203 (149, 277), 528 (415, 671), and 726 (577, 913) in the normal saline, 20 μg SCTV01C, and 40 μg SCTV01C groups, respectively.

The GMTs of anti-Omicron (BA.1) neutralizing antibodies significantly increased 4.41-fold and 5.11-fold from baseline on Day 28 after booster immunizations with 20 and 40 μg of SCTV01C, respectively, and changed slowly within 90 days after the booster immunizations.

On Day 28 after booster immunization, the GMTs of anti-Delta neutralizing antibodies were 1280 (1045, 1567), 3525 (2902, 4282), and 4112 (3545, 4768) in the normal saline, 20 μg SCTV01C, and 40 μg SCTV01C groups, respectively. The GMTs on Day 90 were 1201 (969, 1487), 2686 (2165, 3333), and 3028 (2557, 3587) in the normal saline, 20 μg SCTV01C, and 40 μg SCTV01C groups, respectively. The GMTs of anti-Delta neutralizing antibodies significantly increased 3.96-fold and 4.14-fold from baseline on Day 28 after booster immunizations with 20 and 40 μg of SCTV01C, respectively, and changed slowly within 90 days after the booster immunizations.

ELISpot assay on Day 14 after SCTV01C booster immunization showed that the study vaccine effectively induced Th1 and Th2 responses (4.7- and 2.1-fold increases, respectively) compared to normal saline. However, activated T cell responses were numerically similar in the 20 μg and 40 μg SCTV01C groups (161 in the 20 μg group and 204 in the 40 μg group).

1.5.4 PHASE III CLINICAL TRIAL IN UAE: BOOSTER IMMUNIZATION STUDY (SCTV01C-E-01-UAE-1)

1.5.4.1 Safety results of study SCTV01C-E-01-UAE-1

SCTV01C-E-01-UAE-1 is a randomized, double-blind, active-controlled phase III clinical trial (NCT05323461) conducted in UAE to evaluate the immunogenicity and safety of SCTV01E and SCTV01C (bivalent recombinant COVID-19 Alpha/Beta variant S-ECD protein trimer vaccine) in people ⩾ 18 years of age who have been previously vaccinated with an inactivated COVID-19 vaccine (Cohort 1) or an mRNA vaccine (Cohort 2). As of September 29, 2022, 1800 subjects had received 1 dose of the study vaccine, including 1350 subjects in Cohort 1 and 450 subjects in Cohort 2.

In Cohort 1, 446 were in the SCTV01E group, 453 in the SCTV01C group, and 451 in the inactivated COVID-19 vaccine (BBIBP-CorV, manufactured by the Beijing Institute of Biological Products) group. The overall incidence of adverse reactions was similar across the SCTV01E, SCTV01C, and BBIBP-CorV groups, and there was no significant difference in the incidence of solicited and unsolicited adverse reactions, most of which were Grade 1–2. A total of 15 cases of Grade ⩾3 adverse reactions occurred in this study, all of which were fever, including 5 subjects (1.1%) in the SCTV01E group, 4 subjects in the SCTV01C group, and 6 subjects (1.3%) in the BBIBP-CorV group. The solicited adverse reactions (⩾ 3%) were injection site pain and fever; none of the unsolicited adverse reactions exceeded 3%. See **Table 10** for details.

In Cohort 2, 147 subjects were in the SCTV01E group, 154 in the SCTV01C group, and 149 in the Pfizer mRNA COVID-19 vaccine BNT162b2 group. Adverse reactions were mostly Grade 1–2. A total of 1 case of Grade ≥3 adverse reactions occurred in this cohort, which was fever, in the SCTV01C group. The solicited adverse reactions (≥ 3%) were injection site pain; none of the unsolicited adverse reactions exceeded 3%. See **Table 10** for details.

Table 10 Number of Subjects (%) with Adverse Reactions in Cohort 1 of Clinical Trial SCTV01C-E-01-UAE-1

	**BBIBP-CorV (N = 451) n (%)**	**SCTV01C (N = 453) n (%)**	**SCTV01E (N = 446) n (%)**
**Overall adverse reactions**	62 (13.7)	67 (14.8)	70 (15.7)
**Solicited ARs**	38 (8.4)	50 (11.0)	55 (12.3)
Solicited local ARs	15 (3.3)	27 (6.0)	31 (7.0)
Injection site pain	12 (2.7)	25 (5.5)	30 (6.7)
Injection site erythema	2 (0.4)	2 (0.4)	2 (0.4)
Injection site swelling	1 (0.2)	2 (0.4)	2 (0.4)
Solicited systemic ARs	26 (5.8)	25 (5.5)	26 (5.8)

	BBIBP-CorV (N = 451) n (%)	SCTV01C (N = 453) n (%)	SCTV01E (N = 446) n (%)
Fever	16 (3.5)	14 (3.1)	10 (2.2)
Headache	9 (2.0)	8 (1.8)	13 (2.9)
Myalgia	2 (0.4)	8 (1.8)	6 (1.3)
Fatigue	2 (0.4)	1 (0.2)	3 (0.7)
Joint pain	1 (0.2)	1 (0.2)	0 (0.0)
**Unsolicited ARs**	31 (6.9)	22 (4.9)	27 (6.1)

Table 11 Number of Subjects (%) with Adverse Reactions in Cohort 2 of Clinical Trial SCTV01C-E-01-UAE-1

	BNT162b2 (N = 149) n (%)	SCTV01C (N = 154) n (%)	SCTV01E (N = 147) n (%)
**Overall adverse reactions**	19 (12.8)	24 (15.6)	14 (9.5)
**Solicited ARs**	15 (10.1)	19 (12.3)	7 (4.8)
Solicited local ARs	8 (5.4)	11 (7.1)	1 (0.7)
Injection site pain	8 (5.4)	11 (7.1)	1 (0.7)
Injection site induration	1 (0.7)	0 (0.0)	0 (0.0)
Injection site swelling	1 (0.7)	0 (0.0)	0 (0.0)
Solicited systemic ARs	7 (4.7)	10 (6.5)	6 (4.1)
Headache	3 (2.0)	4 (2.6)	1 (0.7)
Fever	2 (1.3)	2 (1.3)	3 (2.0)
Myalgia	3 (2.0)	1 (0.6)	2 (1.4)
Fatigue	0 (0.0)	2 (1.3)	1 (0.7)
Chills	0 (0.0)	1 (0.6)	0 (0.0)
**Unsolicited ARs**	9 (6.0)	7 (4.5)	7 (4.8)

1.5.4.2 Immunogenicity results of study SCTV01C-E-01-UAE-1

The immunogenicity data analysis points in Project SCTV01C-E-01-UAE-1 were Day 0 and Day 28 after one booster dose. Its primary immunogenicity endpoint was the geometric mean titer (GMT) of neutralizing antibodies against SARS-CoV-2 mutants (Omicron BA.1/BA.5 and Delta). Seroresponse rate (Day 28) is defined as the proportion of subjects with pre-immunization neutralizing antibody titers below the minimum lower limit of detection and post-immunization neutralizing antibody titers greater than or equal to the minimum lower limit of detection; or those with pre-immunization neutralizing antibody titers above the maximum lower limit of detection and post-immunization neutralizing antibody titers of 4-fold or more significant increase. Per-protocol set for immunogenicity (i-PPS): all randomized subjects who received the study vaccine and had at least 1 evaluable immunogenicity data before and after vaccination.

In Cohort 1, on Day 28 after one booster dose of SCTV01E on the basis of previous complete inactivated COVID-19 vaccination, the neutralizing antibody titers against Omicron

BA.1 and BA.5 and Delta in the SCTV01E group increased 28.06-fold, 21.11-fold, and 15.88-fold from baseline, respectively, and were 9.56 times, 8.61 times, and 7.26 times of the BBIBP-CorV group, respectively (all *p*-values < 0.0001); the seroresponse rates were all 92.6%. The immunogenicity of the SCTV01C group was also significantly higher than that of the BBIBP-CorV group. See **Table 12** for details.

Table 12Authentic Virus Neutralizing Antibody Titers and Seroresponse Rates (%) in UAE Phase III Cohort 1

		Control group			SCTV01C			SCTV01E		
**Test item**	**Time point**	**n**	**GMT (95% CI)**	**Seroresponse rate**	**n**	**GMT (95% CI)**	**Seroresponse rate**	**n**	**GMT (95% CI)**	**Seroresponse rate**
Omicron BA.1	Before immunization	100	92 (69, 122)		98	65 (49,87)		95	69 (51,93)	
	Day 28 after immunization	100	219 (167,286)	28	98	1262 (1056,1509)	87.8	95	1926 (1557,2382)	92.6
Omicron BA.5	Before immunization	100	157 (121,204)		97	139 (106,182)		95	125 (95,165)	
	Day 28 after immunization	100	324 (251,419)	23	97	2203 (1872,2593)	88.7	95	2636 (2227,3120)	92.6
Delta	Before immunization	100	338 (257,444)		98	327 (252,425)		95	300 (231,389)	
	Day 28 after immunization	100	667 (541,823)	18	98	4171 (3545,4906)	86.7	95	4760 (3939,5752)	92.6

In Cohort 2, on Day 28 after one booster dose of SCTV01E on the basis of previous mRNA COVID-19 vaccination, the neutralizing antibody titers against Omicron BA.1 and BA.5 in the SCTV01E group were 5.96 times and 4.94 times of the baseline, respectively, and 1.55 times (*p*-value < 0.0001) and 1.28 times (nominal *p*-value = 0.0069) of the BNT162b2 group, respectively, both with superiority; the seroresponse rates were 76.3% and 63%. See **Table 13** for details.

Table 13 Authentic Virus Neutralizing Antibody Titers and Seroresponse Rates (%) in UAE Phase III Cohort 2

		BNT162b2			SCTV01C			SCTV01E		
**Test item**	**Time point**	**n**	**GMT (95% CI)**	**Seroresponse rate**	**n**	**GMT (95% CI)**	**Seroresponse rate**	**n**	**GMT (95% CI)**	**Seroresponse rate**
Omicron BA.1	Before immunization	143	259 (216,310)		150	331 (279,392)		139	278 (231,336)	
	Day 28 after immun	143	1049 (923,1193)	61.5	150	1189 (1027,1376)	52	139	1659 (1445,1904)	76.3

	ization									
Omicron BA.5	Before immunization	143	388 (328,461)		150	544 (465,637)		139	461 (387,547)	
	Day 28 after immunization	143	1687 (1471,1936)	66.4	150	1736 (1517,1987)	46	138	2281 (1993,2610)	63

1.5.5 DOMESTIC PHASE III CLINICAL TRIAL: BOOSTER IMMUNIZATION STUDY (SCTV01E-MRCT-2)

1.5.5.1 Safety results of study SCTV01E-MRCT-2

The SCTV01E-MRCT-2 clinical trial (NCT05308576) was conducted in China to evaluate the safety and effectiveness of SCTV01E in protecting against COVID-19 in individuals who have already received COVID-19 vaccination for initial or booster immunization. This randomized, double-blind, and placebo-controlled phase III trial involved 9196 participants, with 4595 receiving the placebo and 4601 receiving SCTV01E as of May 10, 2023..

During the clinical trial, 159 out of 4,562 subjects (3.5%) who received a placebo reported solicited local adverse events (AEs). In comparison, 679 out of 4,569 subjects (14.8%) who received SCTV01E reported local AEs. All of the reported solicited local AEs were related to the study vaccine. The most common solicited local AEs were injection site pain (3.0% for the placebo group vs 14.0% for the SCTV01E group), injection site pruritus (0.7% vs 2.6%), injection site swelling (0.1% vs 1.8%), injection site induration (0.1% vs 0.6%), and injection site erythema (0.1% vs 0.6%). Injection site pain was more frequent in the SCTV01E group and was mainly adjuvant-related. Most solicited local AEs were Grade 1 or 2. Only six subjects in the SCTV01E group (0.1%) reported Grade ⩾3 local AEs, including four subjects with injection site pain and two subjects with injection site swelling, all of which had recovered. Most of the local AEs occurred on the day of vaccination or the following day and resolved on their own within 1-2 days without requiring any treatment.In this study, 192 subjects (4.2%) in the placebo group and 291 subjects (6.3%) in the SCTV01E group reported solicited systemic adverse events (AEs). Out of these, 146 (3.2%) and 246 (5.3%) subjects, respectively, reported solicited systemic AEs related to the study vaccine. The solicited systemic AEs reported included fatigue (placebo vs. SCTV01E = 1.4% vs. 2.8%), headache (1.6% vs. 2.5%), fever (1.2% vs. 1.4%), myalgia (1.0% vs. 2.3%), arthralgia (0.7% vs. 0.8%), diarrhea (0.5% vs. 0.6%), chills (0.5% vs. 0.8%), nausea (0.4% vs. 0.8%), and vomiting (0.2% vs. 0.3%). There was no significant difference in the incidence of solicited systemic AEs between the two groups. The majority of the systemic AEs occurred 2–3 days after vaccination and resolved within 1 –

2 days. Most solicited systemic AEs in this study were Grade 1 or 2. However, among Grade ≥3 solicited systemic AEs, fever had the highest incidence, with 21 subjects (0.5%) in the placebo group and 12 subjects (0.3%) in the SCTV01E group reporting it. Unsolicited AEs were reported in 230 subjects (5.0%) in the placebo group and 255 subjects (5.5%) in the SCTV01E group, which were related to the study vaccine in 33 (0.7%) and 47 (1.0%) subjects, respectively.

SAEs occurred in 38 subjects (0.8%) in the placebo group and 40 subjects (0.9%) in the SCTV01E group in this study, none of which were related to the study vaccine. No AESIs occurred in this study. Only 1 subject in the placebo group was withdrawn from the study due to death from a car accident. The overall AE occurrence is presented in the following table.

Table 14 SCTV01E-MRCT-2 TEAE Summary

	Placebo (N = 4595)<br>n (%)	SCTV01E (N = 4601)<br>n (%)
TEAE	477 (10.4)	939 (20.4)
Within 7 days after vaccination	350 (7.6)	817 (17.8)
Within 28 days after vaccination	477 (10.4)	939 (20.4)
TRAE	284 (6.2)	775 (16.8)
Within 7 days after vaccination	281 (6.1)	774 (16.8)
Within 28 days after vaccination	284 (6.2)	775 (16.8)
Grade ≥3 TEAEs	56 (1.2)	56 (1.2)
Grade ≥3 TRAEs	14 (0.3)	17 (0.4)
SAE	38 (0.8)	40 (0.9)
SAEs related to the study vaccine	0	0
AEs leading to study termination	1 (< 0.1)	0
TRAEs leading to study termination	0	0
AESI	0	0
Death	1 (< 0.1)	0

Table 15 Solicited Local AEs in SCTV01E-MRCT-2

PT<br>Severity grade	Placebo (N = 4595)<br>n (%)	SCTV01E (N = 4601)<br>n (%)
At least one solicited local AE	159 (3.5)	679 (14.8)
Injection site pain	139 (3.0)	646 (14.0)
Injection site pruritus	32 (0.7)	120 (2.6)
Injection site swelling	4 (< 0.1)	82 (1.8)
Injection site erythema	3 (< 0.1)	29 (0.6)
Injection site induration	3 (< 0.1)	27 (0.6)

Table 16 Solicited Systemic AEs in SCTV01E-MRCT-2

PT<br>Severity grade	Placebo (N = 4595)<br>n (%)	SCTV01E (N = 4601)<br>n (%)
At least one solicited systemic AE	192 (4.2)	291 (6.3)
Nausea	20 (0.4)	36 (0.8)
Fever	54 (1.2)	65 (1.4)
Diarrhea	24 (0.5)	27 (0.6)
Joint pain	32 (0.7)	38 (0.8)
Chills	21 (0.5)	36 (0.8)
Myalgia	48 (1.0)	107 (2.3)
Vomiting	7 (0.2)	13 (0.3)
Fatigue	64 (1.4)	131 (2.8)
Headache	75 (1.6)	115 (2.5)

1.5.5.2 Protective potency results of study SCTV01E-MRCT-2

This report's primary efficacy data analysis sets are PPE and PPE-14 (protective efficacy). A total of 2,623 subjects (28.4%) were included in the Patient Population Evaluable (PPE) analysis, comprising 1,309 subjects (28.4%) in the placebo group and 1,314 subjects (28.5%) in the SCTV01E group. Additionally, a total of 2,513 subjects (27.2%) were included in the PPE-14 analysis, comprising 1,236 subjects (26.8%) in the placebo group and 1,277 subjects (27.7%) in the SCTV01E group.The specific protective efficacy analysis results are as follows:

Primary endpoint:

In the PPE analysis set, 75 subjects in the placebo group and 23 subjects in the SCTV01E group experienced COVID-19 with any symptoms 7 days after vaccination. The incidence was 257.18 per 1000 in the placebo group and 75.21 per 1000 in the SCTV01E group. The protective efficacy of SCTV01E was 69.36% (95% CI: 50.56%, 81.01%), showing superiority (p-value < 0.0001).

Secondary endpoints:

In the PPE population, the protective efficacy against SARS-CoV-2 infection was 60.75% (43.87%, 72.55%). The protective efficacy against COVID-19 with obvious symptoms was 70.59% (42.22%, 85.03%). The protective efficacy against the number of asymptomatic infection events was 42.90% (1.03%, 67.05%) (p-value = 0.0458). The above data further demonstrate that 7 days after inoculation, SCTV01E can not only prevent COVID-19 of any severity but also play a vital role in preventing infection.

In the PPE-14 population, the protective efficacy of SCTV01E against COVID-19 with any symptoms, COVID-19 with obvious symptoms, SARS-CoV-2 infection, and asymptomatic infection 14 days after inoculation was further improved. The protective efficacy

against SARS-CoV-2 infection was 82.36% (57.90%, 92.61%). Moreover, the vaccine protective efficacy of SCTV01E against COVID-19 with any symptoms was 79.67% (95% CI: 51.03%, 91.56%). The protective efficacy against COVID-19 with obvious symptoms was 73.37% (95% CI: 34.32%, 89.20%). All 4 asymptomatic infections occurred in the placebo group.

No deaths or severe COVID-19 occurred 7 days after inoculation. Only 1 moderate COVID-19 occurred in the placebo group.

Analysis of protective efficacy by subgroup:

The stratified analysis and endpoint analysis of COVID-19 of any severity and SARS-CoV-2 infection 7 and 14 days after SCTV01E inoculation were consistent. SCTV01E was effective in different populations and COVID-19 of different severity, especially for the elderly over 60 years of age and the high-risk population with chronic diseases.

In the PPE population, the protective efficacy of SCTV01E for the elderly over 60 years of age was consistent with that for those aged 18–59 years. The protective efficacy of SCTV01E against COVID-19 with any symptoms was 69.42% (47.31, 82.25) and 69.16% (15.75, 88.71) for those aged 18–59 years and ≥ 60 years, respectively; it was more effective in preventing COVID-19 with any symptoms in the high-risk population with chronic diseases, with protective efficacy of 76.32% (36.77, 91.13) and 66.08% (41.22, 80.42) in people with and without chronic diseases, respectively. These results were obtained 7 days after vaccination.

The COVID-19 events in this study were collected from January to early May 2023, during which Omicron variants BA.5, BF.7, DY series, and XBB were predominant. XBB increased from 0.2% in February to 74.4% at the end of April and 95.2% in early May. When XBB was predominant, the protective efficacy of SCTV01E remained at 51.77%, with a total of 7 XBB cases detected in the sequenced samples (SCTV01E group: placebo group = 1:6). These results showed that SCTV01E not only had good persistence of protective efficacy, but also good broad-spectrum cross-protective efficacy against XBB and other variants 4 months after inoculation.

In conclusion, SCTV01E has been found to exhibit strong protective efficacy against COVID-19 infection, regardless of its severity, 7 days after inoculation. This is particularly promising for high-risk populations, such as individuals over 60 years of age and those with chronic diseases. Furthermore, the protective efficacy of SCTV01E is even more pronounced 14 days after inoculation. Not only does SCTV01E have good persistence of protective efficacy, but it also has broad-spectrum cross-protective efficacy against XBB and other variants, even 4 months after inoculation.

1.6 SAFETY OF SCT-VA02B ADJUVANT

SCTV01E and SCTV01C are prepared using an in-house adjuvant (SCT-VA02B adjuvant) with the same composition as MF59 adjuvant. MF59 adjuvant was approved for use in influenza vaccines in 1997, the second adjuvant approved for use in human vaccines after aluminum adjuvant. MF59 adjuvant has been approved for marketing in more than 30 countries, with more than 150 million vaccinations. Clinical trials have demonstrated that MF59 reduces the amount of antigen, broadens the width of the immune response, and enhances the protective efficacy over the human body. It also shows good safety in sensitive populations such as infants, pregnant women, and the elderly.

The safety of SCTV01E and SCTV01C was evaluated in 4 clinical trials. In one of the trials, SCTV01C-02-1 (China) was designed with an adjuvant control group to assess the safety of the adjuvant. The results showed that the SCT-VA02B adjuvant group had good safety profile. The safety of the adjuvant was also indirectly reflected in the good safety observed in each vaccine dose group in other studies.

1.7 STUDY SIGNIFICANCE

The objective of this study is to assess the safety and immunogenicity of SCTV01E-2 in Chinese populationspopulations' ⩾ 3 years of age previously fully vaccinated with COVID-19 vaccines. The COVID-19 outbreak and pandemic have had a significant impact on the global healthcare system, posing a serious threat to human health and survival. At present, the marketed vaccines are all designed based on the original strain. However, SARS-CoV-2 is a single-stranded RNA virus that is prone to deletion mutations. Newly emerging high-risk mutants like Omicron increase the transmission of COVID-19 and significantly reduce the protective effect of antibodies generated by previous infections or vaccinations.

Therefore, there is an urgent need for vaccines that provides high protection against high-risk variants

1.8 BENEFIT-RISK ASSESSMENT

1.8.1 RISK ASSESSMENT

1.8.1.1 Risks of inoculation with the test vaccine

SCTV01C and SCTV01E are primary products of SCTV01E-2, with good safety demonstrated by the results of preclinical studies and available safety data from the conducted clinical trials. SCTV01C and SCTV01E are safe in subjects who have received COVID-19 vaccines. In addition, based on the fact that SCTV01E-2 is developed based on SCTV01E and has the same pharmacological and toxicological properties as SCTV01E, a protein structure similar to that of SCTV01E, and the same manufacturing process platform and quality control

system as SCTV01E, no significant difference in safety between SCTV01E-2 and SCTV01E is expected. Meanwhile, as with other vaccines, inoculation with this test vaccine has a potential risk of allergic reactions and there is no guarantee that the test vaccine has a protective effect on all vaccinated persons.

1.8.1.2 Vaccine-enhanced disease

The mechanism of antibody-dependent enhancement (ADE)/vaccine-enhanced disease (VED) is unknown, and there are no specific clinical indications and laboratory indicators for clinical diagnosis. Nonetheless, ADE/VED has a specific association with non-neutralizing antibodies. ADE occurs when cells internalize non-neutralizing virus-antibody complexes recognized by Fc receptors, leading to increased viral uptake and exacerbating viral infection. VED can occur via ADE, activation of leukocyte differentiation antigen 4-positive (CD4+) memory T cells, Th2 deviation, or abnormal T cells, thereby increasing the ability of viruses to enter host cells and self-replicate. The results of preclinical studies showed that no ADE/VED occurred after SCTV01C inoculation, but risk control measures need to be established preclinically, and all subjects should be closely monitored and followed up throughout the study.

1.8.1.3 Risks of collection of biological samples

Venipuncture is a common clinical procedure used to collect blood samples. Mild pain and rare dizziness are immediate complications.In addition, venipuncture may cause a hematoma, but the risk is minimal. Skin/soft tissue infections at puncture sites, veins, or blood streams can occur, but are rare in finger and venous blood collection. The risk associated with the nasal/nasopharyngeal/oropharyngeal swab collection process is low. Some people may cough and sneeze briefly after swabbing, and others may experience irritation or slight bleeding in their nasal passages.

1.8.1.4 During the study, a trained, qualified, and experienced medical professional will closely oversee the collection of venous blood samples and nasal/nasopharynx/oropharyngeal swabs. This will be done following the prescribed procedures to reduce the risk of any discomfort or harm to the subject. This includes minimizing the risk of local pain, venipuncture site infections, and nasal mucosal damage.**Exposure during pregnancy**

Currently, there has been no study conducted on the reproductive toxicity of the vaccine. However, based on the experience with marketed recombinant protein vaccines, a more reasonable contraceptive period has been set for this study. This means that individuals who participate in the clinical trial must take effective contraceptive measures between the time they sign the informed consent form and 6 months after they receive the study vaccine.Acceptable and highly effective methods of contraception include:

- Combined hormonal (estrogen- and progestogen-containing) contraception (oral, intravaginal, or transdermal) to prevent ovulation;
- Progesterone-only contraception (oral, injectable, or implantable) to prevent ovulation;
- Intrauterine device or intrauterine hormone-releasing system;
- Bilateral tubal occlusion or ligation;
- Sexual partner without reproductive function;
- Abstinence (prohibition of sexual intercourse).

During this period, the investigator will maintain contact with the subject to confirm the occurrence of pregnancy and related complications.

1.8.2 BENEFIT ASSESSMENT

All subjects will undergo physical examination (including but not limited to routine physical examination, vital signs, and SARS-CoV-2 testing) and will be informed of the test results.

Participation in this study will contribute to a better understanding of COVID-19 to develop better preventive measures. If SCTV01E-2 can successfully prevent COVID-19, it can better protect subjects from SARS-CoV-2 infection, which will also significantly contribute to global public health progress.

1.8.3 OVERALL BENEFIT-RISK ASSESSMENT AND RISK PREVENTION AND CONTROL MEASURES

Control plans and measures for potential risks such as allergic reactions and ADE/VED will be developed prior to the conduct of the clinical trial. The inclusion and exclusion criteria will be strictly adhered to during the study. Throughout the study, all participants will be closely monitored and followed to ensure their safety and best interests. Investigators will be trained to collect safety data and follow up with subjects, while also being informed promptly of any adverse events that occur. Overall, the risks associated with SCTV01E-2 are manageable, and the safety profile of this vaccine supports the initiation of this phase II study for the potential benefit of COVID-19 prevention.

2 STUDY OBJECTIVES AND ENDPOINTS

2.1 STUDY OBJECTIVES

Primary objectives:

- To evaluate the immunogenicity of SCTV01E-2 in subjects aged 18 years and above who have been previously vaccinated with SARS-CoV-2 vaccine;
- To evaluate the immunogenicity of SCTV01E-2 in subjects aged 3–17 years who have been previously vaccinated with SARS-CoV-2 vaccine;

Secondary objective:

- To evaluate the safety of SCTV01E-2 in subjects aged 3 years and above who have been previously vaccinated with SARS-CoV-2 vaccine;

Exploratory objective:

- To evaluate the protective efficacy of SCTV01E-2 against COVID-19 with any symptoms occurring 7 days after vaccination.

2.2 STUDY ENDPOINTS

Primary endpoints:

- Geometric mean titer (GMT) of neutralizing antibodies (authentic virus based neutralization assay) against the current predominant strains of SARS-CoV-2 at 14 days after vaccination (the current predominant strains are based on the predominant epidemic types during the assay/serological detection);
- Seroresponse rate (SRR) of neutralizing antibodies (authentic virus based neutralization assay) against the current predominant strains of SARS-CoV-2 at 14 days after vaccination;

Secondary endpoints:

Immunogenicity

- GMTs and SRRs of neutralizing antibodies against other SARS-CoV-2 variants at 14 days after vaccination (other variant types will be adjusted according to the changes in the epidemic strains);
- GMTs and SRRs of neutralizing antibodies against the current predominant strains and/or other variants of SARS-CoV-2 at 180 days after vaccination;

Safety

- Incidence and severity of solicited adverse events (AEs) on Days 0–7 after vaccination;
- Incidence and severity of unsolicited AEs on Days 0–28 after vaccination;
- Incidence and severity of serious adverse events (SAEs) and adverse events of special interest (AESIs) within 365 days after vaccination.

Exploratory endpoint:

- The number of COVID-19 events with any symptoms occuring for the first time after Day 7 post vaccination (since Day 8).

3 STUDY DESIGN

Many studies have shown that the neutralizing activities against various SARS-CoV-2 variants significantly decrease after the completion of primary immunization, mainly due to a decrease in antibody titers, and hence a booster immunization is needed [12-16]. Most studies adopt a 3 to 6 months duration or more as the interval between the last dose of primary and booster immunization [17-20]. Considering the safety of the subjects and the study of SARS-CoV-2 vaccine for booster immunization that is ongoing, "subjects at 6 months and above after the last dose of primary or booster immunization" is used as one of the inclusion criteria.

3.1 STUDY DESIGN

This is an immunogenicity bridging phase II clinical trial of SCTV01E-2 with its predecessor SCTV01E. We aimed to evaluate the immunogenicity and safety of SCTV01E-2 in subjects of different age groups, who have been vaccinated with approved SARS-CoV-2 vaccine. SCTV01E has completed a phase III protective efficacy study and has been approved for emergency use in China. SCTV01E-2 is an updated modified version of SCTV01E.

A total of at least 600 subjects ≥ 3 years of age previously vaccinated with SARS-CoV-2 vaccine approved for domestic use (conditional marketing or emergency authorization for use) with the recommended dose and immunization schedule are planned to be included in this study. Subjects aged 18 years and above are included in Group A, and subjects aged 3–17 years are included in Group B. Subjects will be tested for baseline IgM before vaccination, and the number of subjects with positive baseline IgM should be supplemented to ensure a minimum of 400 subjects with negative baseline IgM in Group A and 200 subjects with negative baseline IgM in Group B. In addition, subjects may be appropriately enrolled based on the percentage of subjects infected with SARS-CoV-2 within 14 days of vaccination (prior to D14 immunogenicity sampling).

All subjects in Group A will be randomized 1:1 to receive one dose of SCTV01E-2 or SCTV01E after enrollment. Fourteen sentinel subjects aged 18–59 will be observed for 7 days following administration of the investigational vaccine for safety assessment by IDMC, and if the criteria for suspension/termination of the study are not met, non-sentinel subjects in Group A will continue to be enrolled and enrollment in Group B will then be initiated.

The randomization stratification factors for Group A include age (18 - 59 years, ≥ 60 years), history of SARS-CoV-2 infection (yes, no), and interval of previous vaccination/infection (6 - 11 months, ≥ 12 months). The interval between previous vaccination/infection is defined as the interval between the time of the last dose of

vaccination/last SARS-CoV-2 infection (whichever is later) and the time of ICF signing. In Group A, the proportion of subjects aged ≥ 60 years shall be no less than 40%.

Group B is divided into the following age groups: 3–5 years, 6–11 years, 12–17 years, and all subjects are vaccinated with one dose of SCTV01E-2. Fourteen sentinel subjects per age group will be enrolled sequentially in order of decreasing age. Initially, 14 sentinel subjects aged 12–17 years will be enrolled and observed for 7 days after receiving the investigational vaccine. Following IDMC's safety assessment, if the criteria for suspension/termination of the study are not met, non-sentinel subjects aged 12–17 years and 14 sentinel subjects aged 6–11 years will be enrolled simultaneously. Furthermore, sentinel subjects aged 6–11 years will be observed for 7 days after being vaccinated with the investigational vaccine for safety assessment by IDMC, and if the criteria for suspension/termination of the study are not met, non-sentinel subjects aged 6–11 years and 14 sentinel subjects aged 3–5 years will be enrolled simultaneously. Finally, sentinel subjects aged 3–5 years will be observed for 7 days after being vaccinated with the investigational vaccine for safety assessment by IDMC, and non-sentinel subjects aged 3–5 years will be enrolled if the criteria for suspension/termination of the study are not met.

The study design is shown in **Figure 2**.

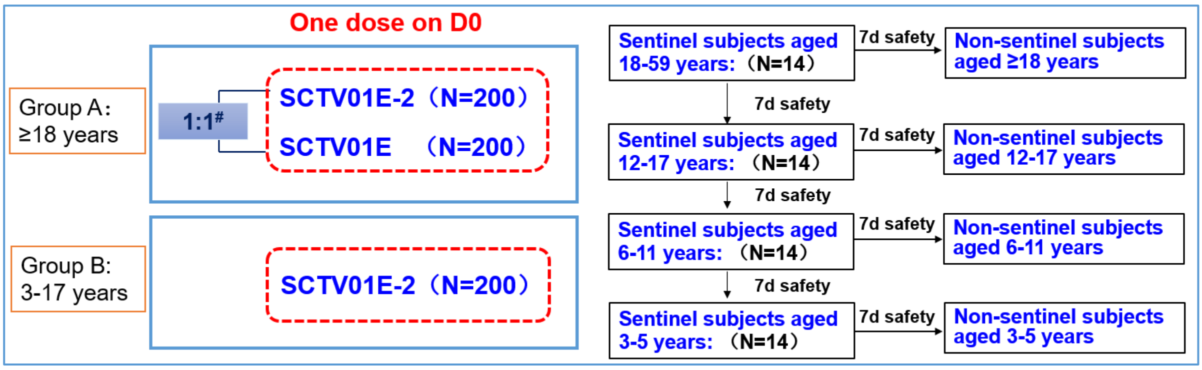

Figure 2 The Schema of Study Design

Screening period:

After the subjects sign ICF, a screening visit will be conducted within 7 days prior to study vaccination to review eligibility.

Randomization:

Subjects eligible for screening in Group A will be randomized prior to vaccination.

Safety follow-up:

Subjects will be observed at the study site for at least 30 minutes after study vaccination (which could be appropriately extended by the investigator according to the subjects'

condition), and the solicited and unsolicited AEs at the vaccination site (local) and non-vaccination site (systemic) will be reported during this period. A combination of active surveillance and spontaneous reporting is used to collect solicited AEs for up to 7 days and unsolicited AEs for up to 28 days after vaccination for all subjects, as well as SAEs and AESIs for up to 365 days after study vaccination. Twenty-eight days after vaccination, subjects will be followed up by regular telephone calls at a frequency of at least once a month, which may be adjusted according to actual conditions.

Enrolling additional subjects in the corresponding age group will continue after the sentinel subject has completed the 7-day post-vaccination safety assessment. Meanwhile, the first 20 subjects in each age group (⩾ 18 years, 12–17 years, 6–11 years, 3–5 years) will be sampled for laboratory tests on Day 3 after vaccination.

Follow-up for immunogenicity:

Blood samples will be collected from subjects prior to study vaccination, on Day 14 and Day 180 after vaccination. These samples will be used to detect total IgG antibodies against SARS-CoV-2 (pre-vaccination only) as well as to determine the neutralizing antibody titers against the current predominant strains and other variants of SARS-CoV-2 (the current predominant strains will be based on those epidemic types that are prevalent during the assay/serological detection, and the other variants will be adjusted according to the changes in the epidemic strains).

Follow-up for protective efficacy:

After vaccination, we will start monitoring the effectiveness of the vaccine by periodically checking in with the subjects for signs/symptoms related to COVID-19. This can be done via phone call, SMS, email, or during on-site visits.. The frequency of these follow-ups may be adjusted according to the trial's progress. However, subjects are encouraged to report any COVID-19-related symptoms they experience at any time during the study. Subjects will undergo SARS-CoV-2 antigen or nucleic acid test if any of the relevant COVID-19 clinical symptoms is/are met.

If a subject receives or requires any other SARS-CoV-2 vaccine during the study, he/she should be withdrawn.

3.2 RATIONALE FOR STUDY DESIGN

3.2.1 SELECTION OF STUDY POPULATION

At present, there are nearly 50 kinds of COVID-19 vaccines approved for marketing or authorized for emergency use worldwide. As a result, and there are many kinds of vaccines administered to the general public. Considering that SARS-CoV-2 is likely evolve into a virus

similar to flu in the future , the development and vaccination model of SARS-CoV-2 vaccines will also become similar to that of influenza vaccines. Therefore, it is more consistent with current and future clinical practice to enroll a mixed population who has previously received a full course of primary or booster immunization with COVID-19 vaccines.

3.2.2 RATIONALE FOR SELECTION OF CONTROL GROUP

According to the *Guidelines for the Development and Evaluation of Prophylactic Vaccines against SARS-CoV-2 Variants* (Trial) issued by the Center for Drug Evaluation[9], immuno-bridging studies should be conducted on modified vaccines with first-generation vaccines to predict the efficacy of the modified vaccines based on the protective efficacy data of the first-generation vaccines. SCTV01E-2 is a modified vaccine of SCVTV01E.An efficacy efficacy clinical study has been conducted with SCTV01E. Therefore, SCTV01E will be used as the control vaccine in this immuno-bridging study.

3.2.3 RATIONALE FOR SELECTION OF DOSES

SCTV01E has demonstrated good immunogenicity and safety in several clinical studies at a clinical dose of 30 μg. Therefore, the same total antigen dose as SCTV01E is selected as the clinical dose of SCTV01E-2, and the proportion of each antigen is adjusted according to the animal immunogenicity study. The dose of each antigen in SCTV01E-2 is 5 μg of TM23 protein, 5 μg of TM41 protein, 10 μg of TM41F protein, and 10 μg of TM41H protein.

3.3 BLINDING AND UNBLINDING PROTOCOL

3.3.1 RANDOMIZATION

In this clinical trial, Group A will follow a randomized, double-blind, controlled design. An independent third-party randomization statistician will use SAS software version 9.4 or above to generate the randomization scheme. Subjects will be randomized to SCTV01E-2 and SCTV01E groups in protocol-specified proportions across study periods with randomization stratification factors including age (18–59 years, ⩾ 60 years), history of SARS-CoV-2 infection (yes, no), and interval of previous vaccination/infection (6–11 months, ⩾ 12 months). Eligible subjects will be randomized by the Interactive Web Response System (IWRS) and receive study vaccines according to random numbers. For subjects who withdraw from the study for any reason after randomization, regardless of whether they are inoculated with the study vaccine or not, their random numbers will be retained.

In the event of discoloration, damage, etc. of the study vaccine, the vaccinator should report to the on-site director and principal investigator blinded and obtain a new vaccine number through the randomization system.

All subjects in Group B will be vaccinated with SCTV01E-2.

3.3.2 BLINDING AND UNBLINDING PROCEDURES

Blinding

In this study, a double-blinded design is adopted for Group A. During the conduct of the trial, a third-party, an independent unblinded statistician should ensure that the blind code of the random code is properly preserved and handed over to the project blinded statistician after the end of the project to be kept with other project-related materials.

Unblinding

The unblinding time will be determined by the sponsor and investigator based on the progress of the study. Relevant documents must be jointly signed by the principal investigator, sponsor, and statisticians before unblinding.

Emergency unblinding

Emergency unblinding is allowed in case of any serious adverse event or emergency occurring during the conduct of the trial, when the investigator believes that knowing the group of subjects is of great importance for their clinical treatment or health. The principal investigator or his/her designee in charge shall directly contact the sponsor to discuss the necessity of unblinding under emergency. Emergency unblinding for individual subjects can be carried out after being confirmed by the sponsor. The investigator or his/her designee shall apply for emergency unblinding in IWRS and record the reasons for unblinding. Then the subject may be withdrawn from the study, and the reason for withdrawal should be recorded in the original data.

For subjects who choose to remain in the study after unblinding, they will continue to be followed up for safety.

3.4 DEFINITION OF END OF STUDY

Definition of end of study for individual subject: The subject has completed all visits as specified in the protocol, or terminated the study prematurely for any reason.

Definition of end of study: The last subject to be enrolled has completed the last visit as specified in the protocol, or terminated the study prematurely for any reason.

4 STUDY POPULATION

4.1 INCLUSION CRITERIA

Subjects are eligible to be included in the study only if the following criteria are met:

1) Individuals aged ≥ 3 years when signing the ICF;
2) Subjects who have been vaccinated with the SARS-CoV-2 vaccine approved for domestic use (conditional marketing or emergency authorization for use) with the recommended dose and immunization schedule, and the interval from the last dose of SARS-CoV-2 vaccine to signing the ICF is ≥ 6 months;
3) The subject and/or his/her legal guardian or delegate can sign the written ICF and voluntarily participate in the trial, and can fully understand the trial procedure, the risk of participating in the trial, and other interventions that can be selected if they do not participate in the trial;
4) Subjects and/or their legal guardians or delegates are able to read, understand, and complete the diary/contact card;
5) Healthy subjects or subjects with stable underlying diseases. Stable underlying disease is defined as a stable condition for at least 3 months prior to enrollment in this study, with no significant changes in treatment plan, and no hospital admission due to disease progression;
6) Males of reproductive potential and females of child-bearing potential who voluntarily agree to take effective contraceptive measures from signing the ICF to 6 months after administration of study vaccine; females of child-bearing potential who have negative pregnancy test results at screening.

4.2 EXCLUSION CRITERIA

Subjects who meet any of the following criteria should be excluded from this study:

1) Fever (for > 14 years old, axillary temperature ≥ 37.3°C; for ≤ 14 years old, axillary temperature ≥ 37.5°C) within 72 hours prior to administration of the study vaccine;
2) A positive result of nucleic acid test or antigen test for SARS-CoV-2 during the screening period;
3) Known history of SARS-CoV-2 infection (including asymptomatic and symptomatic SARS-CoV-2 infection) in the past 6 months before signing the ICF;
4) History of severe allergy, such as severe skin eczema, dyspnea, throat edema, angioneurotic edema, etc., or allergy to the investigational vaccine and its components;
5) History or family history of seizure, epilepsy or psychosis;
6) Subjects at acute phase of illness, such as acute onset of chronic heart failure, acute sore throat, hypertensive encephalopathy, acute pneumonia, acute renal insufficiency, and acute

cholecystitis;

7) Subjects who currently have contraindications for intramuscular injection or intravenous blood sampling, including thrombocytopenia and other blood coagulation disorders;
8) Patients on antituberculosis therapy;
9) Subjects vaccinated with influenza vaccine within 14 days or with other vaccines within 28 days before the study vaccination;
10) Subjects who have received investigational drug treatment of other clinical trial within 1 month before study vaccination;
11) Subjects who have received any immunoglobulin or blood products in the previous 3 months before vaccination, or plan to receive similar products during the study;
12) Those who donated blood or had blood loss (⩾ 450 mL) within 3 months before the vaccination or plan to donate blood during the study period;
13) Pregnant or lactating women;
14) Those who plan to donate ovum or sperm during the study period;
15) Those who cannot follow the trial procedures, or cannot cooperate to complete the study due to planned relocation or long-term outings;
16) Those unsuitable for participating in the clinical trial as determined by the investigator because of other abnormalities that are likely to confuse the study results, or non-conformance with the maximal benefits of the subjects;

4.3 DEFINITION OF SCREENING FAILURES

Subjects who signed the ICF but were not randomized, including those who did not meet inclusion criteria or met criteria but were not randomized, are considered screening failures.Subjects who are randomized but not administered are not considered screening failures, and their status should be set as early withdrawal.

Screening failures must have the following data records completed:

- Screening stage allocation page (including reasons for screening failure, reasons for not receiving treatment, etc.);
- Informed consent;
- Demographics;
- Inclusion/exclusion criteria (only record inclusion/exclusion that has been completed).

4.4 WITHDRAWAL CRITERIA

4.4.1 WITHDRAWAL BY THE SUBJECTS

The subjects have the right to withdraw from the study in advance at any phase of the study, or they are lost to follow-up because they fail to receive follow-up visits according to the trial requirements, although they have not explicitly proposed to withdraw from the study. The investigator should try to contact the subjects who withdraw from the study in advance, record the causes for their early withdrawal in the original data, and inform the study team of the case.

4.4.2 WITHDRAWAL BY INVESTIGATOR

Withdrawal determined by the investigator is a decision by the investigator to withdraw an enrolled subject from the study in case the subject is found to be inappropriate to continue the study during the study process, and the reasons may include:

1) Subjects developed intolerable AE, and the investigator determined that continuing the study would be detrimental to the subject’s health;
2) Major protocol deviation that may affect the safety of the subject;
3) Other reasons that disqualify subjects from continuing this study as determined by the investigator.

4.4.3 HANDLING OF SUBJECT WITHDRAWAL

The investigator is responsible for making possible effort to contact subjects who miss their scheduledfollow-up appointments. The investigator will follow up with subjects who withdraw from the study due to SAEs or AEs until the AEs disappear, ease, stabilize, or have other outcomes. If a subject withdraws from the study, the nasal/nasopharyngeal/oropharyngeal swabs and blood samples collected before the withdrawal date can still be used for study analysis, unless the subject specifically requests otherwise.

The investigator shall record relevant information about withdrawal from the study in the electronic case report form (eCRF), including who made the withdrawal decision, the subject or the investigator; and a detailed record of the specific circumstances:

1) Loss to follow-up;
2) For death, the investigator shall record the cause of death;
3) For voluntary withdrawal, the investigator shall record the withdrawal status and reason;
   - Withdrawal status:
     a) Subjects voluntarily withdraw from all studies, including all study activities such as biological sample collection and safety observation;
     b) Subjects voluntarily withdraw from part of the study, such as only stopping

biological sample collection, and other studies specified in the protocol shall be continued;

- Reason for withdrawal:
  a) Subjects request to withdraw due to reasons related to the study, such as intolerance of AEs and intolerance of biological sample collection;
  b) Subjects request to withdraw due to reasons unrelated to the study, such as long-term outings and relocation.

4) It is determined by the investigator that the subject should withdraw from the study;
5) Other reasons.

If a subject withdraws or terminates the study (including loss to follow-up) after enrollment, they will not be replaced.

4.5 STUDY SUSPENSION/TERMINATION CRITERIA

4.5.1 STUDY SUSPENSION CRITERIA

The study shall be suspended and the investigator, sponsor, and IDMC shall hold a meeting to decide whether any adjustments are necessary for the clinical trial if subjects in each age group (⩾ 18 years, 12–17 years, 6–11 years, 3–5 years) experience one of the following:

- AEs related to the study vaccine with a severity of Grade 3 or above occur in ⩾ 15% of subjects who receive the study vaccine;
- Any AE of Grade 4 or above related to the study vaccine occurs during the study;
- Any SUSAR related to the study vaccine occurs during the study.
- For special AEs, the investigator and the sponsor will decide whether to suspend or terminate according to specific circumstances to ensure the safety of subjects.

4.5.2 STUDY TERMINATION CRITERIA

In one of the following situations, the trial should be suspended or terminated:

- When the sponsor requires a suspension/termination of the trial and gives reasons for it;
- When the Ethics Committee requires a suspension/termination of the trial and gives reasons for it;
- When the regulatory authority requires a suspension/termination of the trial and gives reasons for it.

5 DESCRIPTION OF STUDY PROCEDURES AND VISITS

The study procedures and assessments required are specified in the protocol. If there is a protocol deviation due to an emergency, accident, or error, the investigator or designated personnel must notify the sponsor immediately.

Assessments not specified in the protocol may be performed by the investigator due to clinical needs.

This study includes a screening period, a vaccination period, and a follow-up period (including safety follow-up and immunogenicity follow-up). The follow-ups will be performed by on-site and remote visits (e.g., telephone, SMS, email, etc.).

5.1 V1 (SCREENING PERIOD, D-7 TO D0)

Before vaccination, subjects will sign an informed consent form (ICF) and undergo screening from 7 days prior to vaccination until the day of vaccination. During this screening period, baseline data will be collected to determine whether the subject meets the inclusion and exclusion criteria for the study. The investigator will record in the original document and eCRF the data collected during the screening period, including screening number, date of birth, medical or vaccination history, examination results, screening date, enrollment status, and reasons for non-eligibility with the enrollment (if applicable).

The following items must be completed before enrollment:

- Confirmation and collection of ICFs signed by subjects and/or legal guardians or the persons entrusted;
- Assignment of screening numbers;
- Review of inclusion/exclusion criteria;
- Demographic data: including date of birth (age calculated based on date of birth), sex, ethnicity, height, weight, and BMI (calculated based on height and weight). A valid and working phone number and/or email or other contact information is also required. Changes in communication methods during subsequent visits should be updated (if applicable);
- Record of medical history: including the vaccination history of COVID-19 vaccine, SARS-CoV-2 infection history, other vaccination history and medication treatment history within 28 days before the signing of ICFs, major previous surgical history, allergy history, and other major disease history;
- Vital signs: including blood pressure, respiration rate, pulse, and body temperature;
- Physical examination: including general conditions, head and neck, lymph nodes, skin, chest, abdomen, musculoskeletal system, and other examinations deemed necessary

at the discretion of the investigator;

- SARS-CoV-2 rapid antigen or nucleic acid tests of nasal/nasopharyngeal/oropharyngeal swabs: All subjects should undergo rapid antigen or nucleic acid tests upon screening at the study site. Subjects with positive results will be excluded from this study;
- Pregnancy test: WOCBP only (see Appendix I for definition). Urine pregnancy tests are routinely performed, and blood pregnancy tests can be performed if the investigator considers it necessary;

5.2 V2 (D0, VACCINATION PERIOD)

Eligible subjects will be randomized to receive SCTV01E-2 or SCTV01E. The visit content in the vaccination period is as follows (Please note that there is no need to repeat items such as review of inclusion/exclusion criteria, vital signs, and pregnancy tests if the screening and vaccination are done on the same day):

- Review of inclusion/exclusion criteria;
- Vital signs: including blood pressure, respiration rate, pulse, and body temperature;
- Pregnancy test: WOCBP only (see Appendix I for definition). Urine pregnancy tests are routinely performed, and blood pregnancy tests can be performed if the investigator considers it necessary;
- Detection of anti-SARS-CoV-2 IgM;

Follow-up for immunogenicity

Before vaccination, collect a blood sample to detect total IgG antibodies against SARS-CoV-2 and neutralizing antibodies against current strains and variants.

Randomization and vaccination:

- Randomization: only applicable to subjects in Group A;
- Administering the study vaccines;

Safety follow-up

- Laboratory tests [only for the first 20 subjects in each age group (⩾ 18 years, 12–17 years, 6–11 years, 3–5 years)]: Blood samples will be collected prior to vaccination;
- Observation for at least 30 minutes after vaccination (which can be adjusted appropriately by the investigator according to the specific conditions of the subject);
- Distribution of thermometers and rulers, and instructing subjects to measure and record body temperature and local erythema, rash, redness, and induration;
- VRC distribution: VRCs will be distributed after study vaccination;
- Solicited AEs and unsolicited AEs;

- SAEs and AESIs;
- Record of concomitant medications;

Follow-up for protective efficacy

- Follow-up for protective efficacy: The follow-up will be conducted through phone calls, short messages, emails, or on-site visits, once weekly; the frequency of the follow-up may be adjusted according to the progress of the study.

Subjects will be provided with emergency contact numbers and asked to immediately contact designated medical centers in case of emergency medical needs after vaccination. Subjects will be provided with thermometers and rulers and instructed to record symptoms and signs, as well as the severity of AEs within 28 days after vaccination. The subjects should record the solicited AEs and unsolicited AEs within 7 days after vaccination, as well as the unsolicited AEs and concomitant medication information within 28 days after vaccination on their Diary Cards/Contact Cards.

5.3 V3 (D3 + 1D)

This visit only applies to the first 20 subjects in each age group (⩾ 18 years, 12–17 years, 6–11 years, 3–5 years).

Safety follow-up

- Laboratory tests;
- Solicited AEs and unsolicited AEs: Solicited AEs and unsolicited AEs within 0–3 days after vaccination will be collected;
- SAEs and AESIs;
- Review but no collection of VRCs;
- Record of concomitant medications;

Follow-up for protective efficacy

- Follow-up for protective efficacy: The follow-up will be conducted through phone calls, short messages, emails, or on-site visits, once weekly; the frequency of the follow-up may be adjusted according to the progress of the study.

5.4 V4 (D7+3D)

Safety follow-up

- Solicited AEs and unsolicited AEs: Solicited AEs and unsolicited AEs within 0-7 days after vaccination will be collected;
- SAEs and AESIs;
- Review and collection of VRCs;
- Distribution of contact cards;

- Record of concomitant medications;

Follow-up for protective efficacy

- SARS-CoV-2 rapid antigen or nucleic acid tests of nasal/nasopharyngeal/oropharyngeal swabs;
- Follow-up for protective efficacy: The follow-up will be conducted through phone calls, short messages, emails, or on-site visits, once weekly; the frequency of the follow-up may be adjusted according to the progress of the study.

5.5 V5 (D14+3D)

Follow-up for immunogenicity

- Blood sample collection: used for detection of neutralizing antibodies against the current predominant strains and other variants of SARS-CoV-2;

Safety follow-up

- Unsolicited AEs: Unsolicited AEs within 7–14 days after vaccination will be collected;
- SAEs and AESIs;
- Review but no collection of contact cards;
- Record of concomitant medications;

Follow-up for protective efficacy

- SARS-CoV-2 rapid antigen or nucleic acid tests of nasal/nasopharyngeal/oropharyngeal swabs;
- Follow-up for protective efficacy: The follow-up will be conducted through phone calls, short messages, emails, or on-site visits, once weekly; the frequency of the follow-up may be adjusted according to the progress of the study.

5.6 V6 (D28+7D)

Safety follow-up

- Unsolicited AEs: Unsolicited AEs within 14-28 days after vaccination will be collected;
- SAEs and AESIs;
- Review and collection of contact cards;
- Record of concomitant medications;

Follow-up for protective efficacy

- Follow-up for protective efficacy: The follow-up will be conducted through phone calls, short messages, emails, or on-site visits, once weekly; the frequency of the follow-up may be adjusted according to the progress of the study.

5.7 V7 (D180 ± 20D)

Follow-up for immunogenicity

- Blood sample collection: used for detection of neutralizing antibodies against the current predominant strains and other variants of SARS-CoV-2;

Safety follow-up

- SAEs and AESIs;
- Record of concomitant medications (Twenty-eight days after vaccination, only concomitant medication for SAE and AESI treatment related to the study vaccine will be collected, as well as prohibited medications);

Follow-up for protective efficacy

- Follow-up for protective efficacy: The follow-up will be conducted through phone calls, short messages, emails, or on-site visits, once weekly; the frequency of the follow-up may be adjusted according to the progress of the study.

5.8 V8 (EOS, D365 ± 20D)

This visit can be followed up remotely by telephone and other means, or on-site if necessary.

Safety follow-up

- SAEs and AESIs: Twenty-eight days after vaccination, subjects will be followed up by safety telephone calls at a frequency of at least once a month, which may be adjusted according to actual conditions;
- Record of concomitant medications (Twenty-eight days after vaccination, only concomitant medication for SAE and AESI treatment related to the study vaccine will be collected, as well as prohibited medications);

Follow-up for protective efficacy

- Follow-up for protective efficacy: The follow-up will be conducted through phone calls, short messages, emails, or on-site visits; the frequency of the follow-up may be adjusted according to the progress of the study.

5.9 UNSCHEDULED CONTACT AND FOLLOW-UP

The investigator may conduct unscheduled contact and follow-up (visits not specified in the regular schedule) at the request of the subject or as needed during the study period. All unscheduled contacts and follow-ups will be recorded in the subject's original documents and eCRF.

6 STUDY VACCINES

6.1 BASIC INFORMATION ON STUDY VACCINES

Test vaccine:

Name:	Recombinant S-Trimer Protein Subunit [Beta/Omicron (BA.1/BQ.1. 1/XBB.1) Variant] Vaccine for SARS-CoV-2 (SCTV01E-2)
Ingredients:	• Main active ingredients: TM23 protein, TM41 protein, TM41F protein, TM41H protein;<br>• Adjuvant: SCT-VA02B, which is an oil-in-water emulsion containing squalene;<br>• Excipients: citric acid, sodium citrate, sodium chloride, Polysorbate 80.
Dosage form:	Injection
Appearance:	Milky, white suspension liquid
Strength:	0.5 mL/vial (for 1 person)
Storage conditions:	Store and transport at 2–8°C, protected from light
Route of vaccination:	Intramuscular injection into the lateral deltoid of the upper arm
Shelf life:	24 months
Manufacturer:	Sinocelltech, Ltd.

Control vaccine:

Name:	Recombinant S-Trimer Protein Subunit (Alpha/Beta/Delta/Omicron Variant) Vaccine for SARS-CoV-2 (SCTV01E)
Ingredients:	• Main active ingredients: TM22 protein, TM23 protein, TM28 protein, TM41 protein;<br>• Adjuvant: SCT-VA02B, which is an oil-in-water emulsion containing squalene;<br>• Excipients: citric acid, sodium citrate, sodium chloride, Polysorbate 80.
Dosage form:	Injection
Appearance:	Milky, white suspension liquid
Strength:	0.5 mL/vial (for 1 person)
Storage conditions:	Store and transport at 2–8°C, protected from light
Route of vaccination:	Intramuscular injection into the lateral deltoid of the upper arm
Shelf life:	24 months

Manufacturer:	Sinocelltech, Ltd.

6.2 MANAGEMENT OF STUDY VACCINES

SCTV01E-2 and SCTV01E will be stored and transported at 2－8℃, protected from light. The test vaccine and control vaccine are coded according to relevant regulatory requirements, and vaccine blinding and labeling are performed by the blinding personnel, i.e., printed labels are affixed to corresponding vaccines according to blind codes. The blinding personnel shall not participate in other related procedures of this clinical study or disclose the blind code to any personnel participating in this clinical study.

Study vaccines will be provided by the sponsor free of charge and dispensed to each site as scheduled. The designated personnel of the study site will be responsible for the receipt and storage of the study vaccines, and also for the record of each subject’s administration, drug recycling and maintenance, etc. For Group A, only subjects who are eligible for screening and complete randomization may receive the study vaccine; for Group B, only subjects eligible for screening may receive the study vaccine. All study vaccines must be kept in a safe and environmentally controlled area with manual or automated monitoring according to specified storage conditions and accessible only to investigators and authorized staff. Abandoned, expired, or surplus study vaccines shall be destroyed according to the sponsor's requirements and in accordance with the *Guidelines for the Management of Study Vaccines* or equivalent documents.

6.3 ROUTE OF VACCINATION AND DOSE

Administration site: the lateral deltoid of the upper arm;

Route of vaccination: intramuscular injection;

Vaccination dose: 30 μg/0.5 mL for SCTV01E-2; 30 μg/0.5 mL for SCTV01E.

The injection site is disinfected with 75% alcohol before injection, and the study vaccine is injected intramuscularly after the skin is slightly dry. Prior to vaccination, the study vaccine shall be gently shaken thoroughly to suspend and then withdrawn. If any abnormality of the vaccine is found, such as abnormal color, broken vials, insufficient dosage, unclear label, etc., the vaccine shall not be used, and backup vaccines should be used for administration. All subjects shall be observed at the study site for at least 30 min after the vaccination. Appropriate emergency medical treatment measures shall be prepared at the study site to prevent possible allergic reactions after vaccination.

Note: The deltoid muscle of the non-dominant arm is the preferred injection site. It is

forbidden to vaccinate within 2 cm of a tattoo, scar, or skin defect. Standard vaccination methods should be strictly followed and no vaccine shall be injected into blood vessels. For additional precautions regarding vaccination, please refer to the Investigator's Brochure for SCTV01E-2.

6.4 VACCINATION SCHEDULE

Prior to each vaccination, the information of the subject and the study vaccines must be checked. All subjects in Group A will receive 1 dose of test vaccine (SCTV01E-2) or control vaccine (SCTV01E) on D0; all subjects in Group B will receive 1 dose of test vaccine (SCTV01E-2) on D0.

6.5 CONCOMITANT MEDICATION

Concomitant medications are medications other than the study vaccines taken by a subject during the study, including concomitant medications for all AEs occurring within 0–28 days after vaccination, concomitant medications for vaccine-related SAEs/AESIs occurring 28 days after vaccination, and prohibited medications taken by a subject during the study.

The information on the concomitant medications, including drug name, purpose of administration, dosage and administration, and administration time, must be recorded in eCRF in detail.

6.5.1 PERMITTED CONCOMITANT MEDICATION

The following medications are allowed during the study:

- Medications used to control concomitant diseases that will not interfere with the assessment of study results during the study as judged by the investigator are allowed to continue to be used during the study;
- If the subjects experience adverse events, it is allowed to provide necessary drug therapies;
- If the subject is diagnosed with COVID-19 after vaccination, treatment can be given according to the local standards;
- In case of routine immunization during the study, subjects can be vaccinated according to the instructions for use of the products, but the vaccination time shall be at least 14 days apart from the vaccination for study vaccines. Vaccines for medical emergencies, such as rabies or tetanus, can be timely vaccinated according to the instructions for use of the products.

6.5.2 PROHIBITED CONCOMITANT MEDICATION

The following medications are prohibited during the study:

- Any COVID-19 preventive medication;
- Drugs or vaccines (investigational or not marketed) other than study vaccines;
- Immunoglobulin or other blood products;
- Non-prescription drugs such as antipyretic (e.g., acetaminophen) and anti-inflammatory drugs (e.g., ibuprofen, naproxen, etc.) used within 12 h before the administration of the study vaccine.

6.6 COMPLIANCE OF SUBJECTS

Subjects will receive the study vaccine directly from the study site staff. The study site staff will record in detail the date and time of subject vaccination in the original documents and eCRF.

7 COLLECTION, DISPOSAL, AND TEST OF BIOLOGICAL SAMPLES

7.1 COLLECTION AND DISPOSAL OF BLOOD SAMPLES

7.1.1 COLLECTION OF BLOOD SAMPLES

The items for blood sample collection specified in the protocol include:

Blood sample collection for anti-SARS-CoV-2 IgM: Blood samples will be collected from all subjects for anti-SARS-CoV-2 IgM testing prior to vaccination.

Blood sample collection for immunogenicity: Blood samples will be collected from all subjects prior to vaccination and on Day 14 and Day 180 after vaccination for the detection of total IgG antibodies against SARS-CoV-2 (pre-vaccination only) and for the detection of neutralizing antibody titers against the current predominant strains and other variants of SARS-CoV-2.

Blood sample collection for laboratory tests: Blood samples will be collected from the first 20 subjects in each age group (≥18 years, 12–17 years, 6–11 years, 3–5 years) prior to vaccination and on Day 3 after vaccination for laboratory tests (hematology and blood biochemistry).

The items for blood sample collection and sampling volume of subjects are shown in the table below:

Table 17 Items for Blood Sample Collection and Sampling Volume

**The first 20 subjects in each age group (≥18 years, 12–17 years, 6–11 years, 3–5 years)**			
Visit	Item	Sampling volume	**Total sampling volume**
V2 (D0)	Anti-SARS-CoV-2 IgM, anti-SARS-CoV-2 IgG, and neutralizing antibody	6mL	**12mL**
	Blood routine	3mL	
	Blood biochemistry	3mL	
V3 (D3+1)	Blood routine	3mL	**6mL**
	Blood biochemistry	3mL	
V5 (D14+3)	neutralizing antibody	6mL	**6mL**
V7 (D180±20)	neutralizing antibody	6mL	**6mL**
**Other subjects**			
V2 (D0)	Anti-SARS-CoV-2 IgM, anti-SARS-CoV-2 IgG, and neutralizing antibody	6mL	**6mL**
V5 (D14+3)	neutralizing antibody	6mL	**6mL**
V7 (D180±20)	neutralizing antibody	6mL	**6mL**

Other blood samples in the screening period are collected as needed.

7.1.2 DISPOSAL AND RETENTION OF BLOOD SAMPLES

Biological samples collected in this study will be properly preserved as required. During the study or sample retention period, further coronavirus-related testing may be performed on remaining samples for future research, in addition to study endpoint analysis. The subject may

withdraw consent for other future uses of the sample at any time, and, in this case, the sample will be destroyed at the end of the study.

Serum samples shall be stored at -20℃ or lower, as specified in the laboratory manual.

7.1.3 IMMUNOGENICITY TEST

The ELISA method will be adopted for the detection of total IgG antibodies against SARS-CoV-2; the authentic virus-based neutralization assay will be adopted for the detection of neutralizing antibody levels against the current predominant strains and other variants of SARS-CoV-2 (the current predominant strains are based on the predominant epidemic types during the assay/serological detection, and the other variants are adjusted according to the changes in the epidemic strains). The pseudovirus-based neutralization assay may be added according to actual demand.

7.2 COLLECTION, DISPOSAL, AND TEST OF VIRAL TEST SAMPLES

7.2.1 COLLECTION OF VIRAL TEST SAMPLES

Samples will be collected from all subjects for SARS-CoV-2 antigen or nucleic acid detection prior to vaccination, and the results should be negative before vaccination.

Samples will be collected from subjects who meet the criteria for COVID-19 clinical symptoms (refer to Section 8.3) for SARS-CoV-2 antigen or nucleic acid detection and sequencing.

7.2.2 ANTIGEN DETECTION OR VIRUS-SPECIFIC NUCLEIC ACID DETECTION

Rapid test kits approved by regulatory authorities are used for antigen detection.

SARS-CoV-2 nucleic acid detection is performed using RT-PCR and the kit is subject to regulatory approval. RT-PCR testing should be performed in laboratories that meet regulatory requirements. If the detection result of a suspected case is doubtful, the test should be repeated once.

Sequencing is performed in laboratories that meet regulatory requirements and it is decided whether to sequence the samples based on future needs.

7.3 OTHER LABORATORY TESTS

During the study, biological samples may be collected and tested as needed for urinalysis, pregnancy test, and other tests specified in the protocol, and additional laboratory tests required upon the judgment of the investigator according to the clinical indications of subjects in case of adverse events or suspected COVID-19 cases. The investigator determines whether the abnormal indicators for laboratory tests have clinical significance (refer to Appendix II: Guideline for Adverse Event Grading Criteria in Clinical Trial of Preventive Vaccines--NMPA

Standard). If the abnormality is clinically significant, it shall be recorded as an AE and further assessed by the investigator for severity and correlation. If the abnormality is not clinically significant, it will not be reported as an AE but shall be described in detail in the original record.

8 STUDY ASSESSMENT AND REPORT

8.1 SAFETY ASSESSMENT

Subjects will be observed at the study site for at least 30 minutes after each vaccination (which could be appropriately extended by the investigator according to the subjects' condition), and the solicited and unsolicited AEs at the vaccination site (local) and non-vaccination site (systemic) will be reported during this period. A combination of active surveillance and spontaneous reporting is used to collect solicited AEs for up to 7 days and unsolicited AEs for up to 28 days after study vaccination for all subjects, as well as SAEs and AESIs for up to 365 days after study vaccination. The occurring solicited AEs within 7 days after study vaccination and unsolicited AEs within 28 days after study vaccination are recorded on Diary Cards/Contact Cards and the subjects should return to the study site with Diary Cards/Contact Cards at V3–V6. Twenty-eight days after vaccination, subjects should be followed up by safety telephone calls at a frequency of at least once a month.

Safety information will be entered into the eCRF.

8.1.1 DEFINITION

8.1.1.1 AE

AEs are all adverse medical events that occur after the subject receives the study vaccine, which can be manifested as clinically significant symptoms and signs, diseases, or laboratory test abnormalities, but do not necessarily have a causal relationship with the study vaccine. Stable conditions with previous abnormalities that do not change in severity during the study shall be recorded as medical history and not as AEs.

8.1.1.2 ADVERSE REACTIONS

All harmful and unexpected reactions related to any dose of the study products (vaccines) shall be considered adverse drug reactions. There is at least a reasonable probability for the causality between the study drugs (vaccines) and AEs, that is, a relationship cannot be ruled out.

8.1.1.3 SOLICITED AEs

Solicited Adverse Events (AEs) are pre-specified AEs that the researchers actively monitor and record upon the request of the study participants. The investigator will assess the solicited AEs that occur within 30 minutes and 7 days (from Day 0 to Day 7) after each vaccination. The study subjects will keep a record of the occurrence of solicited AEs, along with their severity and any concomitant medications, if taken, in the diary cards provided by the researchers..

Solicited AEs can be divided into injection-site (local) AEs and non-injection-site (systematic) AEs depending on the site of AEs. Detailed information is shown in **Table 18**.

Table 18 List of Solicited AEs

Solicited local AEs	Solicited systemic AEs
• Injection site pain<br>• Injection site erythema<br>• Injection site swelling<br>• Injection site induration<br>• Injection site pruritus	• Fever<br>• Nausea<br>• Vomiting<br>• Headache<br>• Fatigue<br>• Myalgia<br>• Joint pain<br>• Chills

8.1.1.4 UNSOLICITED AEs

Unsolicited AEs are not pre-specified and are not under active surveillance. Unsolicited AEs are AEs reported by subjects other than solicited AEs pre-specified in the protocol or AEs with the same name as solicited AEs that occur after the collection period specified in the protocol for solicited AEs (Day 0 to Day 7).

The investigator assesses the correlation and severity of unsolicited AEs according to the NMPA guidelines in Appendix II.

8.1.1.5 SAE

SAEs are the following medical events after a subject receives any dose of a study vaccine:

- Results in death;
- Life-threatening events (Note: The definition of "Life-threatening" in SAEs means immediate death risk in subjects when the event occurs, other than that death would occur when the condition becomes severe in the future.);
- Events requiring hospitalization or prolongation of hospitalization (generally, hospitalization means that the subject is hospitalized (usually for at least one night) in a hospital or emergency ward for observation and/or treatment);
- Permanent or severe disability or dysfunction;
- Congenital anomaly or birth defect;
- Other significant medical events.

Medical and scientific judgment must be used to determine whether to expeditiously report other situations. For example, significant medical events that may not be immediately life-threatening or result in death or hospitalization but may require medical measures to prevent one of the other outcomes listed above should also be considered serious.

Hospitalization is not considered a SAE due to elective surgery, routine clinical procedures, routine physical examinations, hospital admissions for observation, or protocol

requirements, other than an AE. But if an unexpected event occurs during such hospitalization, it will be reported as a "serious" or "non-serious" AE according to the common criteria.

Note: Hospitalization or prolongation of hospitalization for non-medical reasons/convenience or purely for clinical trial purposes does not meet the criteria for a medical event and therefore cannot be considered an SAE.

8.1.1.6 AESI

AESIs are AEs of special interest to the study vaccines from a scientific or medical perspective.

AESIs will be monitored and collected throughout the study according to the WHO's *List of Adverse Events of Special Interest* (AESIs) [21]. AESIs for this study will be updated or revised as cumulative safety data are collected.

Table 19 List of AESIs Collected after Vaccination

No.	Body system	AESI
1	Heart	Acute cardiovascular injury
2	Skin	Chilblain-like lesions
3	Skin	Single-organ cutaneous vasculitis
4	Skin	Erythema multiforme
5	Endocrine	Acute pancreatitis
6	Endocrine	Subacute thyroiditis
7	Stomach and intestine	Acute liver injury
8	Blood	Coagulation disorder (thromboembolism)
9	Blood	Thrombocytopenia
10	Blood	Thrombosis and thrombocytopenia syndrome (TTS)
11	Immunization	Vaccine-associated enhanced disease (VAED)
12	Immunization	Multi-system inflammatory syndrome
13	Immunization	Allergic reaction
14	Musculoskeletal system	Acute aseptic arthritis
15	Musculoskeletal system	Rhabdomyolysis
16	Nervous system	Acute disseminated encephalomyelitis (ADEM)
17	Nervous system	Bell's palsy
18	Nervous system	Convulsions generalized
19	Nervous system	Guillain-Barre syndrome
20	Nervous system	Meningoencephalitis
21	Kidney	Acute kidney injury
22	Respiration	Acute respiratory distress syndrome

8.1.1.7 SUSPECTED UNEXPECTED SERIOUS ADVERSE REACTION

A suspected unexpected serious adverse reaction (SUSAR) is an AR that is different in nature, severity, consequence, or frequency from the expected risk described in current information about the study drug (e.g., documents such as the Investigator's Brochure). The Investigator's Brochure is used as major document to provide reference information to judge

whether an adverse reaction is expected or unexpected.

8.1.1.8 COVID-19 DISEASE-RELATED EVENTS AND/OR SEQUELAE NOT AS AES OR SAES

Potential COVID-19 disease (including asymptomatic infection) and its sequelae shall not be recorded as AEs. These data will be recorded only on the corresponding pages for COVID-19 disease in the CRF.

Potential COVID-19 disease and its sequelae will not be reported according to the standard procedure for expedited reporting of SAEs, even if the event may meet the definition of an SAE. These events will be documented on the corresponding pages for COVID-19 disease in the participant's CRF.

Note: An event must be recorded and reported as an SAE (rather than a disease-related event) if any of the following occurs:

An event whose intensity, frequency, or duration exceeded expectations in the opinion of the investigator.

Or

An event that the investigator has a reasonable basis to believe is related to the investigational vaccine.

Potential COVID-19 disease and its sequelae will be reviewed by the investigator or physician. Events that are not considered eligible for COVID-19 disease and its sequelae shall be reported as SAEs.

8.1.2 SEVERITY OF ADVERSE EVENTS

Grading of AEs will be determined according to the adverse event grading criteria described in the *Guideline for Adverse Event Grading Criteria in Clinical Trial of Preventive Vaccines* (Appendix II) promulgated and implemented by the China National Medical Products Administration.

Intensity of AEs not involved in the grading table is evaluated according to the following criteria. See Table **Table 20** for details.

Table 20 General Rules for Severity Grading of Other AEs

Grade 1	Grade 2	Grade 3	Grade 4	Grade 5
**Mild:** Short term (< 48 h) or slight discomfort, having no influence on activities and requiring no treatment	**Moderate:** Mild or moderate activity restricted, possibly requiring a hospital visit, and requiring no or mild treatment	**Severe:** Activities significantly restricted, requiring a hospital visit and treatment, and possibly requiring hospitalization	**Critical:** Possibly threatening life, activities severely restricted, requiring intensive treatment	Death

8.1.3 CORRELATION OF AES WITH THE VACCINE

In this study, solicited local AEs are considered vaccine-related.

The investigator should judge the correlation between the vaccine and AEs (solicited systemic AEs, unsolicited AEs, SAEs, and ASEIs).

The investigator shall assess the correlation between AEs and vaccination in a timely manner, determine the correlation based on the information available at the time of reporting, and then make updates based on subsequent information.

The following factors should be considered in the analysis of the correlation between AEs and the study vaccine:

- Whether there is a reasonable chronological order between an AE and the time when the study vaccine takes effect;
- Whether the clinical or pathological manifestations of an AE are consistent with known knowledge of the pharmacology and toxicology of the study vaccine or its classification;
- Whether an AE can be explained by the original disease, by the subject or by environmental factors, etc.

The investigator will use a simple dichotomy for the determination of drug correlation for AEs (related or unrelated). The recommended approach is to inquire whether there is a reasonable possibility. “Yes” or “No”.

Yes: The AE has a reasonable temporal relationship to the study vaccine and it cannot be explained by the subject’s clinical status, concomitant diseases, or concomitant medications; and/or the AE follows the known pattern of response to the study vaccine; and/or the AE is improved or resolved after discontinuation of the study vaccine, and the AE reappears after re-administration of the study vaccine.

No: There is evidence of a trigger for the AE other than the study vaccine (e.g., original disease state, underlying disease, concomitant disease, or concomitant medication); and/or there is no reasonable temporal relationship of the AE to the study vaccine.

8.1.4 OUTCOMES OF AES

Outcomes of AEs can be described as follows:

- Recovered: The “AE end date” should be noted. “Recovered” means returning to baseline levels.
- Recovered with sequelae: Only when the subject has persistent or lifelong sequelae, such as blindness caused by diabetes mellitus and hemiplegia caused by stroke. The “(S)AE end date” should be noted.
- Improved: The event is not resolved completely, but the subjects are recovering.
- Not improved: The event is ongoing.

- Death: The AE causes the death of the subject directly or as a primary cause.
- Uncertain: The investigator is unable to understand the AE, e.g., loss to follow-up of subjects.

The end date of an AE is the date on which the subject is recovered, or recovered with sequelae, or dies.

If the outcome of an AE is assessed as “improved”, “not improved”, or “uncertain”, the AE end date is not required to be recorded temporarily.

If the outcome of an AE is assessed as “recovered” or “recovered with sequelae”, the AE end date must be recorded.

8.1.5 RECORDING OF AES

8.1.5.1 Collection time limit for AEs

The collection time limit is within 7 days after vaccination for solicited AEs, within 28 days after vaccination for unsolicited AEs, and within 365 days after vaccination for SAEs and AESIs.

Collection begins after informed consent is obtained, however, adverse medical conditions occurring prior to vaccination will be documented in the “Medical History/Current Medical Conditions” section of the eCRF rather than the “AE” section.

If the investigator becomes aware of any SAE (including death) at any time after the subject withdraws from the study, and the investigator considers the event to be related to vaccination, the investigator must promptly report the SAE to the sponsor.

For the solicited and unsolicited symptoms experienced by the subject, the investigator shall confirm whether the subject receives treatment such as hospitalization, outpatient, or self-administered medication for any reason, and record such information.

The training for subjects should emphasize the need for timely reporting of AEs. The investigator shall be highly alert to such events and investigate and handle them in a timely manner.

When an SAE occurs, it is the responsibility of the investigator to review all documents related to the event (e.g., hospital progress notes, laboratory reports, and diagnostic reports) to clarify the nature and relevance of the SAE. If a subject’s death is confirmed during his/her participation in the study or during the follow-up period, the hospital’s final findings on the deceased should be collected, and if an autopsy is performed, a copy of the results should be obtained, including histopathological findings.

8.1.5.2 Methods of identifying AEs

At each visit, the following methods may be employed to identify AEs:

- Information provided by a subject or guardian without any prompt; when a subject develops an acute or progressively worsening AR, the investigator or appropriate contact person should be contacted for further treatment advice and/or measures.
- At each visit, asking open-ended and non-suggestive questions such as “How do you feel? Since the last visit, have you had any medical or other issue?”
- Abnormalities observed by the investigator, other medical professionals and relatives.

The investigator will also provide Diary Cards (electronic and/or paper) for subjects to document the solicited AEs occurring from 0 to 7 days after vaccination and unsolicited AEs occurring from 0 to 28 days after vaccination.

8.1.5.3 Recording and follow-up of AEs

The investigator is responsible for recording all AEs and SAEs and reporting them to the sponsor (and/or the CRO designated by the sponsor) within 24 hour of becoming informed of the SAEs. Solicited AEs from Day 0 to Day 7 after vaccination, unsolicited AEs from Day 0 to Day 28 after vaccination, and SAEs and AESIs from Day 0/vaccination to end of study shall be collected. At each on-site visit or remote follow-up, subjects will undergo questioning which includes COVID-19 symptom monitoring to ensure subject safety. Subjects will also be asked if they have been hospitalized, had an accident, taken a new drug, had a change in concomitant medication regimen (including prescription and over-the-counter drugs), or received vaccinations other than the non-study vaccine. Physical examination results or other AE information relevant to subjects’ safety will be recorded. The investigator shall continue to follow up on AEs and SAEs at follow-up visits after completing AE and SAE reporting. AEs and SAEs occurring during the study shall be treated accordingly and followed up until disappearance/recovery, stabilization, other outcomes [no further follow-up is deemed necessary by the investigator for reasonable reasons (e.g., failure to recover, or improvement); additional information is not likely to be available (e.g., the subject refuses to provide additional information, or there is evidence that the subject is still lost to follow-up after the best efforts have been made)] or subject loss to follow-up.

In order to improve the quality and accuracy of AE information collection, the investigator shall follow the guidelines below:

- Use recognized medical terms whenever possible when recording AEs on the eCRF;
- Record the results of diagnosis (i.e., disease or syndrome) rather than associated signs, symptoms, and laboratory test results (e.g., record congestive heart failure rather than dyspnea, rales, and cyanosis);
- Record and report SAEs leading to death;
- For patients hospitalized for a surgery or diagnostic procedure, the disease leading to

the surgery or diagnostic procedure shall be recorded as an SAE rather than the process itself. The process shall be documented in the section on measures taken for disease treatment section for disease treatment in the case narrative;

- Pregnancy occurring in a subject during the study is not considered as an AE per se but should be recorded in a separate pregnancy record form, with the pregnancy report sent to the sponsor (and/or CRO designated by the sponsor). If the outcome of the pregnancy meets the criteria for an SAE (including spontaneous abortion, stillbirth, or any congenital malformation, etc.), the investigator shall report it according to the SAE reporting process.

8.1.6 SAFETY MONITORING

The investigator and/or designated on-site personnel are responsible for monitoring the safety of all subjects and notifying the sponsor in case of unexpected problems.

8.1.7 SAE/SUSAR/PREGNANCY EVENT REPORTING

8.1.7.1 Requirements for expedited reporting by the investigator to the sponsor

The investigator should promptly report SAEs to the sponsor (and/or CRO designated by the sponsor) to meet legal obligations and ethical responsibilities regarding participant safety and safety of the study vaccine in clinical studies.

The following is a list of events (not necessarily related to the study vaccine) that must be reported by the investigator to the sponsor by completing the report form provided by the sponsor within 24 h of awareness (or immediately if the subject dies):

- SAEs;
- Pregnancy.

For these events, the investigator must report new important follow-up information to the sponsor immediately (i.e., no later than 24 h after becoming aware of the information). Important new information includes the following:

- New signs or symptoms, or changes in diagnosis;
- Important new diagnostic test results;
- New information that may lead to a change in the correlation evaluation;
- Change in outcomes of events, including recovered events;
- Other important descriptive information of the event in the clinical course.

All SAEs shall also be recorded in the eCRF. Information provided in SAE report form must be consistent with data recorded in the eCRF regarding the event.

8.1.7.2 Regulatory reporting requirements for SAEs

The sponsor shall promptly report SUSARs to all investigators participating in the clinical trial, study facilities, and Ethics Committee; the sponsor shall report SUSARs to the drug regulatory authorities, health authorities, and other applicable regulatory authorities.

The development safety update reports (DSUR) of the drug provided by the sponsor shall

include an assessment of the risks and benefits of the clinical trial. Relevant information should be notified to all investigators participating in the clinical trial, study facilities, and Ethics Committee.

After receiving the relevant safety information provided by the sponsor, the investigator should sign and read it in time, consider the treatment of the subject and whether it should be adjusted accordingly, communicate with the subject as soon as possible when necessary, and report any SUSAR provided by the sponsor to the Ethics Committee. The investigator shall archive relevant safety information and communications.

8.1.7.3 Pregnancy reporting

If a female subject of childbearing age or the female partner of a male subject becomes pregnant during the study (collection period for pregnancy is the same as for SAEs), the investigator should report it to the sponsor (and/or CRO designated by the sponsor) on an expedited basis by completing the *Pregnancy Report Form* provided by the sponsor in the same timeframe as for SAE reporting.

Pregnancy itself is not considered an AE. Artificial abortion and drug abortion themselves are not recorded as AEs. If spontaneous abortion, birth defect or congenital malformation of neonates, malformation and abnormalities of stillbirth, and serious complications of the mother and the neonate occur during pregnancy period, they should be recorded and reported as SAEs.

During the study, in case of pregnancy of female subjects of childbearing age or female partners of male subjects, the investigator should be immediately informed. The investigator shall give advice to the subjects and discuss the risks of continuing the pregnancy and the possible effects on the fetus. For male subjects, their female partners should be monitored. Follow-up of pregnancy should be continued until at least 12 months after the pregnancy outcome or the birth of the newborn.

Note: Female subjects of childbearing age or female partners of male subjects have the right to know the actual post-blinding grouping information after pregnancy.

8.2 IMMUNOGENICITY ASSESSMENT INDICATORS

Primary assessment indicators:

- Geometric mean titers (GMTs) of neutralizing antibodies (authentic virus based neutralization assay) against the current predominant strains of SARS-CoV-2 at 14 days after vaccination;
- Seroresponse rates (SRRs) of neutralizing antibodies (authentic virus based neutralization assay) against the current predominant strains of SARS-CoV-2 at 14 days after vaccination;

Secondary assessment indicators:

- GMTs and SRRs of neutralizing antibodies against other SARS-CoV-2 variants at 14 days after vaccination (other variant types will be adjusted according to the changes in the epidemic strains);
- GMTs and SRRs of neutralizing antibodies against the current predominant strains and/or other variants of SARS-CoV-2 at 180 days after vaccination;

All analyses will be performed at the sponsor's designated laboratories. Analysis and testing procedures are detailed in the laboratory manual.

8.3 DIAGNOSIS AND TREATMENT OF COVID-19

8.3.1 DEFINITION OF ASYMPTOMATIC INFECTION WITH COVID-19 AND SARS-CoV-2

In this study, SARS-CoV-2 infected subjects are defined as subjects who are positive for SARS-CoV-2 in virological testing (i.e., nucleic acid amplification test or antigen test), with or without COVID-19-related symptoms. SARS-CoV-2 infection includes COVID-19 infection and asymptomatic SARS-CoV-2 infection.

The definition of COVID-19 is based on the following criteria:

COVID-19 with any symptoms is defined as a subject with a positive virological test result (i.e., nucleic acid amplification test or antigen test) who meets the following COVID-19 **clinical symptom criteria**: development of ≥ 1 of the following symptoms: fever, chills, sore throat, generalized weakness/fatigue, muscle pain, headache, anorexia/nausea/vomiting, diarrhea, anosmia or ageusia, cough or expectoration of sputum, breath shortness, dyspnea, clinical or radiographic evidence of pneumonia.

Asymptomatic SARS-CoV-2 infected case is defined as a person who does not develop corresponding symptoms after SARS-CoV-2 infection. The definition is based on the following criteria: subjects with a positive SARS-CoV-2 virological test result (i.e., nucleic acid amplification test or antigen test) who do not have any COVID-19-related clinical symptoms throughout the course of the infection.

8.3.2 SEVERITY GRADING CRITERIA FOR COVID-19 CONFIRMED CASES

The investigator shall closely monitor and treat confirmed patients in accordance with treatment guidelines developed by the National Health Commission.

The investigator may classify cases according to the severity of COVID-19 disease as described in the *Diagnosis and Treatment Protocol for Novel Coronavirus Infections* (Trial Version 10). See **Table 21** for details.

Table 21 Severity of COVID-19Disease

Severity	Definition
Mild	Upper respiratory tract infection as the main manifestation, such as dry throat, sore throat, cough, fever, etc.
Moderate	Persistent hyperpyrexia for > 3 days or (and) cough, tachypnea, etc., but respiratory rate (RR) < 30 breaths/min, and oxygen saturation > 93% on room air at rest. Radiological findings of characteristic SARS-CoV-2 pneumonia.
Severe	Adults with any of the following that cannot be explained by reasons other than SARS-CoV-2 infection:<br>1. Tachypnea, RR ≥ 30 breaths/min;<br>2. At rest, oxygen saturation ≤ 93% on room air;<br>3. Arterial partial pressure of oxygen (PaO_2)/ fraction of inspired oxygen (FiO_2) ≤ 300 mmHg (1 mmHg = 0.133 kPa); in high altitude areas with an altitude of over 1000 meters, PaO_2/FiO_2 should be corrected according to the following formula: $PaO_2/FiO_2 \times$ [760/atmospheric pressure (mmHg)].<br>4. Progressive worsening of clinical symptoms, and significant lesion progression by > 50% within 24–48 h as shown by pulmonary imaging.
Critical	Patients meeting any of the following:<br>1. Respiratory failure and requiring mechanical ventilation;<br>2. Shock;<br>3. With other organ failure that requires ICU monitoring and treatment.

8.3.3 DISCOVERY OF SUSPECTED CASES OF COVID-19

At the first visit, the investigator will provide subjects with detailed instructions on the symptoms and signs of COVID-19 and will instruct them to seek medical attention and notify the investigator if any symptoms are observed. After vaccination, subjects will receive on-site visits and regular remote visits. Subjects may also voluntarily report suspected symptoms of COVID-19. Once COVID-19-related symptoms and signs are identified, COVID-19 diagnostic procedures will be performed (see **Figure 3**).

It should be noted that some symptoms of COVID-19 overlap with actively collected systematic AEs (e.g., myalgia, headache, fever, and chills) that are expected after vaccination. The investigator shall decide, based on his/her clinical judgment, whether a nasal/nasopharyngeal/oropharyngeal swab should be collected when these solicited AEs occur within the first 7 days after vaccination.

8.3.4 COVID-19 DIAGNOSTIC PROCEDURES

Subjects who meet any of the COVID-19 clinical symptoms shall report and undergo nasal/nasopharyngeal/oropharyngeal swab collection (preferably within 72 hours). Two nasal/nasopharyngeal/oropharyngeal swabs will be collected, one for antigen/nucleic acid testing and the other reserved for sequencing. It is decided whether to sequence the backup sample based on future needs.

A subject with a positive test result will be diagnosed with COVID-19 and will be followed up. A subject with a negative test result will have a second sample collected for antigen/nucleic acid testing at least 24 hours apart (but not more than 72 hours). If the subject's

second test result is still negative, the sampling will no longer be repeated. Specific COVID-19 diagnostic procedures are shown in **Figure 3**.

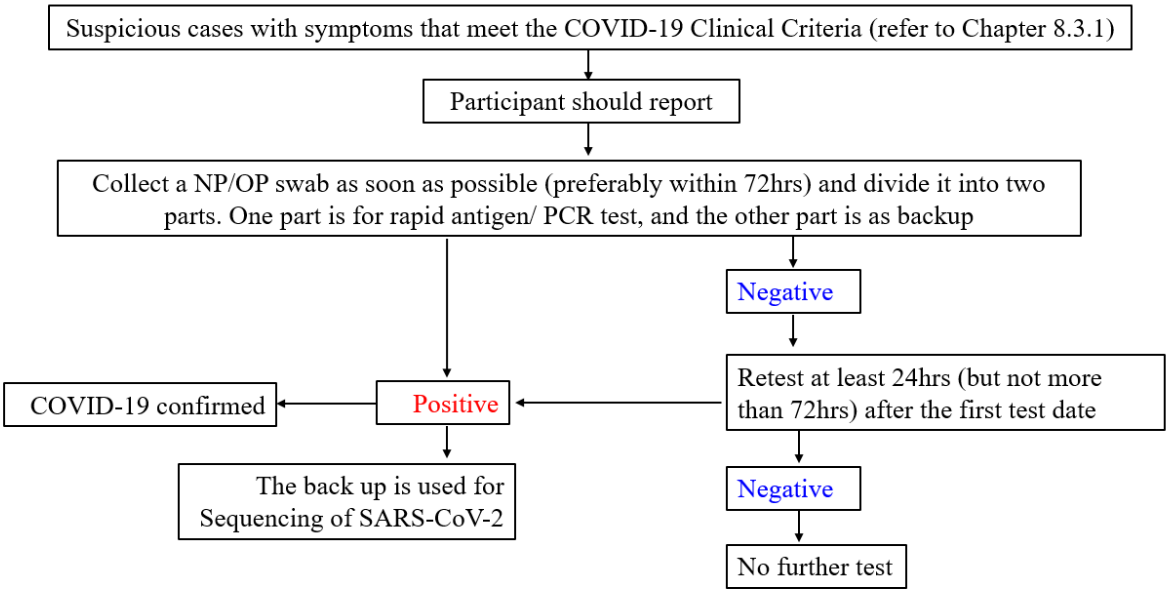

Figure 3 The Diagnostic Procedures of COVID-19

8.3.5 FOLLOW-UP FOR COVID-19 CONFIRMED PATIENTS

COVID-19 confirmed patients are managed and treated in accordance with local policies and regulations. A subject will be followed up by the investigator every 3 to 7 days after he/she has been diagnosed with COVID-19 until symptoms are in sustained resolution, which is defined as the disappearance of all COVID-19-related symptoms, or the recovery of certain specific symptoms (e.g., cough, asthenia, and anosmia or ageusia) that may take longer to recover to a mild level and remain so for more than 48 hours.

The subject's symptoms, time of symptom onset, time of nucleic acid/antigen test sample collection and time of reporting, severity of COVID-19, and outcome are recorded.

8.3.6 VACCINE-ASSOCIATED ENHANCED DISEASES (VAED)/VACCINE-ASSOCIATED ENHANCED RESPIRATORY DISEASES (VAERD)

VAED occurs in vaccine recipients. It refers to the aggravation of clinical symptoms of pathogen infection after the recipients vaccinated and infected with the pathogen that the vaccine is supposed to prevent after vaccination.

VAED may manifest as severe diseases or as abnormal clinical manifestations of known diseases. Patients with VAED may have more severe clinical manifestations; or they may have disease characteristics that make them clearly distinguishable from unvaccinated individuals when infected with the same pathogen;

VAED may involve one or more organs or systems;

In the context of a known incidence of disease associated with SARS-CoV-2 infection, VAED may manifest as an increased incidence of the diseases among vaccine recipients.

VARED is a kind of VAED which primarily involves the lower respiratory tract. Its pathogenesis is more specific than that of lower respiratory tract and some systemic processes. It is commonly used to describe vaccine-associated diseases of respiratory viruses.

In this clinical study, if a subject is diagnosed with COVID-19 or SARS-CoV-2 infection after vaccination with SCTV01E/SCTV01E-2, he/she will be hospitalized or treated in isolation according to the local anti-epidemic prevention and control requirements, and if there are severe cases, critically ill cases, or deaths of COVID-19 among the subjects, special investigations are required for these cases, and the occurrence of VAED/VAERD will be analyzed based on the investigation results.

9 DATA MANAGEMENT

9.1 COMPLETION AND TRANSFER OF SOURCE DATA AND ECRFS

An Electronic Data Capture (EDC) system is used for data collection in this study. Data management plan (DMP): It is drafted by the data manager (DM) as a guidance document for the entire data management procedure. All data management procedures shall be performed according to the time, contents and methods specified in DMP.

An Electronic Data Capture (EDC) system is used for data collection in this study. The data of this study is managed by the sponsor's Data Management Department to ensure the authenticity, completeness, privacy and traceability of clinical trial data.

eCRF: Design data collection forms according to the protocol requirements, define the study process, names of data forms and data items to be collected, and form corresponding eCRF completion instructions, which will be reviewed by the sponsor for use by the study site in completing eCRFs.

The data in eCRF are derived from original medical records and are completed by the investigator or the investigator's designee to ensure that the information is complete and accurate. In case of any error requiring correction, the modification shall be performed in accordance with the filling guide of eCRF, and the EDC system will automatically record the name of the data modifier and the date of modification.

After the data in the EDC system is confirmed to be unquestionable through source data verification (SDV), DM review, query, etc., the investigator is required to perform an electronic signature confirmation before data lock.

9.2 SUGGESTION AND DESIGN OF DATABASE

The design of the eCRF meets the requirements of FDA 21 CFR Part 11, and meets the regulations of the ICH GCP and GCP (NMPA, 2020) for data collection. The data manager performs interface testing, which includes, but is not limited to, page design, visit period settings, order of entry forms at visits, and the order of each data point, etc. The new uniform resource locater (URL) also requires testing of URL configurations, such as the accuracy of browsing permissions for different users. Databases should be established with reference to the standards from the Clinical Data Interchange Standards Consortium (CDISC) wherever possible.

9.3 DATA ENTRY

The investigator should collect subjects' data according to the requirements of GCP and the study protocol. Moreover, the investigator is required to complete the eCRFs in an accurate, timely, complete, and normative manner according to the filling guide. The eCRFs are not regarded as source records.

Data will be entered into EDC database by the investigator or the personnel designated by the investigator. Data entry is carried out in strict compliance with the principle of “what you see is what you record”. After data entry is complete, any changes made to the eCRFs will be automatically recorded in the system.

9.4 DATA VERIFICATION AND QUERY HANDLING

Data verification includes edit checks and manual verification.

The DM will set up the edit check program in EDC system according to the finalized data verification plan.

After the data are entered into EDC system, if there is any illogical data, the edit check will initiate and trigger queries. These queries need to be reviewed and answered by the investigator or by the personnel authorized by the investigator. If the updated data makes the edit check queries no longer valid, the data queries will be automatically closed by the system immediately; if the study site confirms the accuracy of data and gives a reply, DM should review the reply. If justified, the data queries will be closed; if the data issues are not resolved, DM may continue to communicate with the site through the addition of data queries until final resolution.

DM or medical personnel will perform manual data verification during the study period based on subjects’ data listings/reports generated by programming. Artificial queries can be added to EDC system when there is any data requiring clarification/verification/confirmation by the investigator. During source data verification (SDV), clinical research associates (CRAs) may also add manual queries to EDC system if there is any discrepancy between EDC data and source data.

9.5 MEDICAL CODING

Coding includes, but is not limited to, previous medical history, concomitant medications, and AEs.

Previous medical history and AEs will be coded according to the International Medical Dictionary for Regulatory Activities (MedDRA), and concomitant medications will be coded using the World Health Organization Dictionary of Drugs.. All dictionaries used are in the version confirmed by the sponsor.

During the coding process, DM will ask the investigator to verify and confirm any data that cannot be coded due to inappropriate, inaccurate, and ambiguous provision of medical terms.

A medical coding report should be sent by DM for review.

9.6 EXTERNAL DATA CONSISTENCY VERIFICATION

DM will perform external data consistency verification according to the external data handling processes and consistency verification processes defined in the data management plan (DMP). The problematic data will be followed up and resolved in the manner of mail/queries.

9.7 DATABASE LOCKING AND EXPORTING

After all subjects have completed the trial, medical records have been fully entered into the system and all data queries have been resolved, external data consistency has been verified as correct, SAE consistency has been verified as correct, the coding report has been approved by the sponsor, all issues have been resolved during database quality control (QC), and all issues have been resolved after the data review meeting (if any), the database will be locked at the joint discretion of the principal investigator, sponsor, statistician, clinical project manager, and DM. After all data are locked, DM will export data from the system and submit them to the statisticians for statistical analysis. The locked data cannot be edited again. Problems found after the data lock can be corrected in the statistical analysis program after confirmation. If the principal investigator, sponsor, statistical analysis personnel and data manager jointly confirm that there is definite evidence to support the need for unlocking, DM will unlock the data after the Database Unlocking Confirmation Form has been signed by the investigator and the sponsor, etc., and then data updates can be made. All updates must be documented. Database locking process should be performed again after updates are complete.

9.8 RETENTION OF STUDY RECORDS

The basic documents of this clinical trial shall be kept for at least 5 years after the test vaccine is approved for marketing by local regulatory authorities. If not used for approval for marketing, the document shall be kept for at least 5 years after the end of the clinical trial.

The study data will be destroyed upon written notification from the sponsor after the preservation period has expired.

The preservation of all documents related to the trial shall be strictly confidential within the scope specified by local laws.

10 STATISTICAL ANALYSIS

10.1 STATISTICAL HYPOTHESES

The following two co-primary endpoints will be considered in the comparison of SCTV01E-2 versus SCTV01E in the part of Group A and the bridging of SCTV01E-2 in Group B (3–17 years) with SCTV01E-2 in Group A (⩾ 18 years):

- Geometric mean titer (GMT) of neutralizing antibodies of authentic virus after 14 days of vaccination;
- Seroresponse rate (SRR) of neutralizing antibodies of authentic virus after 14 days of vaccination. SRR is defined as the proportion of subjects with a shift in antibody titer from below the lower limit of quantification (LLOQ) on the day of vaccination (pre-vaccination on Day 0) to ⩾ LLOQ post-vaccination or from > LLOQ pre-vaccination to ⩾ 4 times the baseline value post-vaccination.

For GMT endpoints:

- The geometric mean ratio of neutralizing antibody titers for SCTV01E-2 and SCTV01E in Group A will be recorded as GMR_A;
- The geometric mean ratio of neutralizing antibody titers for SCTV01E-2 in Group B (3–17 years) and SCTV01E-2 in Group A (⩾ 18 years) will be recorded as GMR_B;

For SRR endpoints:

- The difference in SRRs between SCTV01E-2 and SCTV01E in Group A will be recorded as Δ_A;
- The difference in SRRs between SCTV01E-2 in Group B (3-17 years) and SCTV01E-2 in Group A (⩾ 18 years) will be recorded as $\triangle_B$.

For Group A, the following superiority hypotheses will be made for GMR_A versus $\triangle_A$:

- For GMR_A, the statistical hypothesis test for superiority will be conducted as follows:

$$\mathbf{H0: GMR_A \leqslant 1, H1: GMR_A > 1 \quad (1)}$$

- For △A, the statistical hypothesis test for superiority will be conducted as follows:

$$\mathbf{H0: \triangle_A \leqslant 0\%, H1: \triangle_A > 0\% \quad (2)}$$

For Group B, the following non-inferiority hypotheses will be made for GMR_B versus $\triangle_B$:

- For GMR_B, the statistical hypothesis test for superiority will be conducted as

follows:

$$\mathbf{H0: GMR_B \leqslant 0.67, H1: GMR_B > 0.67 \quad (3)}$$

- For $\triangle_B$, the statistical hypothesis test for superiority will be conducted as follows:

$$\mathbf{H0: \triangle_B \leqslant -5\%, H1: \triangle_B > -5\% \quad (4)}$$

Statistical tests for (3) and (4) will be performed only if both tests (1) and (2) are statistically significant at a one-sided significance level of 0.025.

In addition, as secondary endpoints, the ratio of neutralizing antibodies against the current predominant strains of SARS-CoV-2 after 14 days of vaccination with SCTV01E-2 in Group A ($\geqslant$ 18 years) to anti-BA.5 neutralizing antibodies after 14 days of vaccination with SCTV01E in the immune subgroup in the SCTV01E Phase III study (SCTV01E-MRCT-2) will be recorded as GMR_C, and the difference in SRRs will be recorded as $\triangle_C$.

The following non-inferiority statistical hypotheses will be made for GMR_C and $\triangle_C$:

- For GMR_C, the statistical hypothesis test will be conducted as follows:

$$\mathbf{H0: GMR_C \leqslant 0.67, H1: GMR_C > 0.67 \quad (5)}$$

- For $\triangle$C, the statistical hypothesis test will be conducted as follows:

$$\mathbf{H0: \triangle C \leqslant -10\%, H1: \triangle C > -10\% \quad (6)}$$

10.2 SAMPLE SIZE

For the hypothesis testing (1), based on the following hypothesis, approximately 288 subjects are included in the test group and the control group, and the trial has a power of 90% to reject H0 and achieve the superiority:

- Difference in $\log_{10}$-transformed common standards of variants in two groups: 0.45
- GMR_A in two groups: 1.5
- Significance level: one-sided 0.025
- Dropout rate: 5%
- Between-group rate: 1:1

For the hypothesis testing (2), based on the following hypothesis, approximately 388 subjects are included in the test group and the control group, and the trial has a power of 90% to reject H0 and achieve the superiority:

- The seroresponse rate for SCTV01E-2 is 80%
- The seroresponse rate for SCTV01E is 65%
- Significance level: one-sided 0.025
- Dropout rate: 5%
- Between-group rate: 1:1

For the hypothesis testing (3), based on the following hypothesis, approximately 300 subjects are included in the test group and control group, and the trial has a power of 90% to reject H0 and achieve the non-inferiority:

- Difference in log_{10}-transformed common standards of variants in two groups: 0.45
- GMR_B in two groups: 1
- Non-inferiority margin: 0.67
- Significance level: one-sided 0.025
- Dropout rate: 5%
- Between-group rate: 1:1

For the hypothesis testing (4), based on the following hypothesis, approximately 392 subjects are included in the test group and control group, and the trial has a power of 80% to reject H0 and achieve the non-inferiority:

- The seroresponse rate for SCTV01E-2 is 80% in Group A (≥ 18 years)
- The seroresponse rate for SCTV01E-2 is 86% in Group B (3–17 years)
- Non-inferiority margin: -5%
- Significance level: one-sided 0.025
- Dropout rate: 5%
- Between-group rate: 1:1

For the hypothesis testing (5), based on the following hypothesis, approximately 368 subjects (at least 184 subjects aged ≥ 18 years in Group A are treated with SCTV01E-2) are included in the test group and the control group, and the trial has a power of 90% to reject H0 and achieve the non-inferiority:

- Difference in log_{10}-transformed common standards of variants in two groups: 0.5
- GMR_C in two groups: 1
- Non-inferiority margin: 0.67
- Significance level: one-sided 0.025
- Dropout rate: 5%
- Between-group rate: 1:1

For the hypothesis testing (6), based on the following hypothesis, approximately 386 subjects (at least 193 subjects aged ≥ 18 years in Group A are treated with SCTV01E-2) are included in the test group and the control group, and the trial has a power of 80% to reject H0 and achieve the non-inferiority:

- The seroresponse rate for SCTV01E-2 is 80% in Group A (≥ 18 years)
- The seroresponse rate of BA.5 in phase III immune subgroup is 78%

- Non-inferiority margin: -10%
- Significance level: one-sided 0.025
- Dropout rate: 5%

Based on the above 6 hypothesis tests, a total of 600 subjects are planned to be enrolled in this trial, of which 400 subjects will be enrolled in Group A and randomized to SCTV01E group or SCTV01E-2 group at a ratio of 1:1; 200 subjects will be enrolled in Group B. The sample size may be adjusted based on the proportion of subjects who are IgM positive at baseline, infection within 14 days of vaccination, and information from outside the trial, to ensure an adequate evaluable population.

10.3 DEFINITIONS OF ANALYSIS POPULATIONS

Full analysis set (FAS): All subjects who received the study vaccine. In the analysis, subjects will be grouped based on the group to which they are randomized.

Per-protocol set (PPS): Subjects included in the FAS who do not have any major protocol violations affecting the critical data of the trial. Major protocol deviations affecting critical data of the trial will be identified and archived prior to trial database lock.

Safety set (SS): All subjects who received the study vaccine. In the analysis, subjects will be grouped according to the vaccine actually vaccinated.

Immunogenicity full analysis set (I-FAS): Subjects included in the FAS who have valid immunogenicity test data before and after immunization.

Immunogenicity per-protocol set (I-PPS): Subjects included in the PPS who have valid immunogenicity test data before and after immunization and are IgM negative at baseline.

SCTV01E phase III (SCTV01E-MRCT-2) immunogenicity per-protocol set (I-PPS): Subjects included in the I-PPS who have valid immunogenicity test data before and after immunization.

The post-infection immunogenicity data from subjects with definite evidence of infection prior to the targeted visit for analysis will be excluded from the analysis.

10.4 STATISTICAL ANALYSIS METHODS

10.4.1 GENERAL RULES

In general, data will be subjected to descriptive statistical analysis and statistical tests according to pre-specified statistical methods. Details of the specific analyses will be described in the SAP.

The number of subjects, mean, standard deviation, median, minimum and maximum are calculated in the description of measurement data. The number and percentage of subjects will be summarized in the categorical data.

For model-based estimates, the estimates, estimated standard errors (if applicable), 95% CIs of the estimates will be provided according to the pre-specified methods in the SAP.

Statistical analyses will be performed using the SAS 9.4 or above.

10.4.2 SUBJECT DISPOSITION AND COMPLETION OF STUDY

The completion status of subjects, including the proportion of subjects who early terminate the trial and the reasons for early termination, will be summarized based on the FAS.

Major protocol violations occurring during the study will be summarized based on the FAS.

10.4.3 DEMOGRAPHIC DATA AND BASELINE CHARACTERISTICS

Demographic data (sex, age, etc.) and other baseline characteristics (physical examination, vital signs, medical history, and previous medications, previous prime immunization or booster vaccination with COVID-19 vaccines, type of previous COVID-19 vaccine, time since last COVID-19 vaccination, etc.) will be summarized and analyzed based on the FAS and I-PPS.

10.4.4 IMMUNOGENICITY ANALYSIS

Immunogenicity data will be analyzed based on the I-PPS.

For neutralizing antibodies at each post-vaccination visit, GMTs and corresponding 95% CIs will be calculated for both groups. Based on the $\log_{10}$-transformed antibody titer, the ANCOVA model is used to calculate the LS (least squares) GMT as well as GMR for between-group comparisons and corresponding 95% CI. Covariates include randomization stratification factors and the $\log_{10}$-transformed baseline level.

For neutralizing antibodies at each post-vaccination visit, SRRs at Day 14 and corresponding 95% CIs will be calculated for both groups (Clopper-Pearson exact method). At the same time, the difference in SRRs between the two groups and 95% CIs (stratified Miettinen-Nurminen method) will be calculated.

10.4.5 SAFETY ANALYSIS

Safety data will be analyzed based on the SS.

Adverse Events

For solicited AEs (solicited local AEs and solicited systemic AEs) within 7 days after immunization, unsolicited AEs within 28 days after immunization, and SAEs and AESIs within 365 days after immunization, the number of subjects, percentage, and severity are summarized according to pre-specified terms.

AEs are coded using MedDRA. For AEs that occur after immunization or increase in severity compared to pre-immunization, classified statistics will be performed according to

SOC and PT, and the number and percentage of subjects will be summarized. If required, 95% CIs of incidences of SAEs and AESIs may be calculated (Clopper-Pearson exact method).

10.5 MISSING DATA

Statistical analysis will be performed based on the actual observed data in the primary analysis of immunogenicity data. Missing data will not be imputed.

AEs with unclear dates will be statistically analyzed as concomitant AEs unless there is definite information indicating that they are not concomitant AEs.

AEs with missing vaccine-related relationships will be statistically analyzed as ‘vaccine-related’ AEs.

10.6 SUBGROUP ANALYSIS

The following subgroup analyses will be performed for the primary endpoint of immunogenicity data and the analysis of safety data. Any additional subgroup analyses will be described in the SAP.

- Sex (male/female)
- Age (3–5 years, 6–11 years, 12–17 years, 18–59 years, ⩾ 60 years)
- History of SARS-CoV-2 infection (yes, no)
- Interval of previous vaccination/infection (6–11 months, ⩾ 12 months)

10.7 SENSITIVITY ANALYSIS

For the primary endpoint of immunogenicity, multiple imputation will be performed for missing data, and explanatory variables for the imputation regression process will take into account group, baseline antibody level, and randomization stratification factors. Results obtained after multiple imputations will be combined based on the Rubin Rules.

10.8 DATA ANALYSIS

When all subjects in Group A have completed the visits on Day 14 and Day 28 after vaccination, a summary of the analyses related to Group A may be performed by an independent unblinded team to support the application. Members from the independent unblinded team will not participate in the subsequent execution of the project. When all subjects in Group B have completed the visits on Day 14 and Day 28, an analysis and summary of the data from Group B may be performed by an independent unblinded team. Subsequent

data analyses will be performed by an independent unblinded team as required by the application.

The final analysis will be performed after all subjects have completed their last visit and after database lock has been completed.

11 CLINICAL TRIAL MANAGEMENT

11.1 STATEMENT

This study will be conducted in accordance with ICH GCP, GCP (NMPA, 2020), Declaration of Helsinki, SOPs of the sponsor and/or its agent (e.g., CRO), and all applicable regulations and laws.

11.2 ETHICS

Before initiation of the study, relevant documents such as the clinical study protocol, ICF, Investigator’s Brochure (IB), etc. need to be submitted to the appropriate Ethics Committee (ERC) for review and approval. The study should not be conducted in any form until written consent/approval has been obtained by the sponsor from the appropriate Ethics Committee. Any amendments to relevant documents such as clinical trial protocol, ICF, etc. must be implemented after obtaining ERC approval.

The investigator and participating researchers shall be familiar with the clinical trial protocol and prepare measures in advance, e.g., solutions and required reports in case of SUSAR.

During the course of the clinical study, any SAE or suspected and unexpected severe adverse reaction (SUSAR) that is related to the safety of the clinical study and may affect the safety of subjects and the conduct of the study will be reported by the investigator to the Ethics Committee (ERC) in accordance with regulatory requirements.

11.3 INFORMED CONSENT

Subjects and/or their legal guardians or delegates must provide informed consent before the subjects receive treatment in this trial to protect their legitimate rights and interests. The principal investigator or investigator of a clinical study is responsible for providing the subjects and/or their legal guardians or delegates with a complete and comprehensive introduction to the purpose, methods, reasonable expected benefits, potential toxic and side effects, and risks of the study. Meanwhile, subjects should be informed that participation in this clinical trial is voluntary, and subjects and/or their legal guardians or delegates should be informed that they have the right to withdraw from the trial at any time, without loss of benefits to which they would otherwise be entitled. Before any operational procedures related to clinical trials, an ICF signed by the subject and/or his/her legal guardian or delegate must be obtained. The ICF is prepared in duplicate, with one copy kept by the subject and the other copy kept in the study archive.

Before obtaining the informed consent form, the investigator and/or his/her designee should provide the subjects with sufficient time and opportunities to consult the details of this

study and to decide whether to participate in this study. The process of the informed consent should be documented in the records on the day of the screening visit.

The investigators should be responsible for the informed consent process. If any information related to the subject’s willingness to continue to participate in this study is obtained during the study period, the written ICF must be updated and then provided to the subject to confirm the subject’s and/or his/her legal guardian’s willingness to continue participation. The amended ICF can be provided for subjects after it is approved by the Ethics Committee.

After signing the ICF, the subjects must also agree that the sponsor, drug approval regulatory authorities, auditors and/or sponsor-authorized clinical trial monitors check the available source data related to the clinical study. Moreover, the reviewer must follow the confidential statement.

The investigator should use the latest version of the ICF approved by ERC and other information provided to the subjects. If any information related to the subject’s willingness to continue to participate in this study is obtained during the study period, the written ICF must be updated and then provided to the subject to confirm the subject’s and/or his/her legal guardian’s willingness to continue participation. The amended ICF can be provided for subjects after it is approved by the Ethics Committee. Subjects can unconditionally withdraw from the trial at any time during the study, and they will not be punished for withdrawing from the trial.

11.4 AMENDMENT OF CLINICAL TRIAL PROTOCOL

During the study, the protocol can be modified based on the communication and approval by the sponsor and the investigator, which can only be implemented after being approved by ERC. Any changes to the protocol, whether major or minor, are required to be made in writing. Substantive protocol revisions that may affect the safety of subjects, the scope of the study, or the scientific quality of this study require approval from all study site ERCs. To protect the safety of all subjects in the study, above requirements shall not hinder the investigator or sponsor from taking any emergency measures. If the investigator believes that immediate changes to the protocol are necessary for safety reasons, he/she must promptly notify the sponsor's designated facility and notify the study site ERC in accordance with the policies formulated by the ERC approving the study, as well as local regulations and policies. Changes that only affect study management do not require substantive protocol revisions or ERC approval, but these changes must be notified to the ERC. In these cases, the sponsor will send an official letter to the ERC detailing these changes.

According to the *Technical Guidelines for Protocol Changes during Drug Clinical Trials*,

some changes need to be notified to regulatory parties.

11.5 PROTOCOL DEVIATION

The investigator should follow a protocol approved by the sponsor, regulatory authorities (if necessary), and ERC.

During the trial, the investigator should not deviate from the protocol unless emergency measures are taken to eliminate direct harm to the subjects. In case of other unexpected situations occurring that require deviation from the prescribed procedures of the protocol, the investigator should consult with medical monitors (and ERC, if necessary) to determine appropriate measures to be taken.

The study site should record all protocol deviations in the original data of the subjects, including but not limited to the occurrence time of protocol deviations, time to uncover, description of events, and measures. If there is a major protocol deviation, the study site should promptly notify the medical monitor, Clinical Research Associate (CRA), or ERC.

11.6 MONITORING

The sponsor and/or their agent (such as CRO) shall conduct clinical monitoring of this study. CRA should conduct monitoring in accordance with the corresponding SOP. CRA should maintain regular communication with the investigator and the sponsor.

Before clinical trial: The CRA should confirm that the investigator has sufficient qualifications and resources to complete the trial, the clinical trial facility has appropriate conditions to complete the trial, including personnel allocation and training, laboratory equipment that is complete and running well, and various examination conditions related to the trial. Meanwhile, the CRA should discuss with the investigator on the specific items required as raw materials, determine the nature and storage location of all raw materials, to ensure that the sponsor or the investigator knows the source of the source data used to complete the eCRF table.

During the clinical trial: The CRA will regularly visit the clinical site (or online access) to review protocol compliance, data integrity, accuracy, and consistency, as well as compliance with ICH GCP and relevant regulations. Based on the risk assessment, remote centralized monitoring can be considered as a substitute or supplement to on-site monitoring. If necessary, the CRA will also provide clarification and additional training to help solve the problems discovered during the monitoring visit on site.

During the study, the investigator should allow the CRA to get direct access to all relevant documents and ensure that the investigator and relevant researchers regularly meet with the CRA to discuss the findings and any related issues during the visit.

11.7 QUALITY ASSURANCE AND AUDIT

During the study, the sponsor or its representative will conduct quality assurance audits on the study site, study database, and related study documents. Meanwhile, relevant regulatory authorities can also decide to inspect study sites, study databases, and related study documents at their own discretion. The purpose is to determine whether the recording, analysis, and reporting of these activities and data comply with the study protocol, GCP, ICH guidelines, and any relevant regulatory requirements. During the audit/inspection, the investigator should support the audit/inspection work and allow the auditor/inspector to directly access source data/documents, including all medical records, documents and correspondences related to the study, as well as informed consent documents for the clinical study.

11.8 INTELLECTUAL PROPERTY

All information obtained from the sponsor is the sponsor’s intellectual property, therefore, the investigator and all other relevant personnel must strictly keep it confidential and not disclose it to any third party without the prior consent of the sponsor.

11.9 PRIVACY OF SUBJECTS

The researchers must safeguard the subject’s privacies. In all documents submitted to the sponsor, the identity of the subject can only be determined by the subject code and initials, while the name or hospitalization number of the subject cannot be indicated. The investigator must strictly keep confidential the names, addresses, and other private information of the subjects, and cannot submit them to the sponsor.

11.10 INDEPENDENT MONITORING COMMITTEE

In this study, an IDMC will be organized by the sponsor to regularly evaluate the progress of the clinical trial, including safety data and immunogenicity endpoint data. Based on the data results, recommendations can be given regarding whether the sponsor can continue, modify, or terminate the ongoing clinical trial.

IDMC members will include vaccine clinical research specialists, biostatisticians and epidemiologists, etc. IDMC should have a prior understanding of the Clinical Trial Protocol, develop and sign the IDMC charter for this study. The primary task of IDMC is to review the safety data reported by subjects after vaccination to protect their safety and benefits. Additionally, IDMC also monitors the entire conduct of clinical trials, including protocol compliance, recruitment status, and subject drop-out rate to ensure the effectiveness and credibility of the trial.

For more relevant information, please refer to the IDMC charter and carry out relevant work in accordance with the requirements of the IDMC charter.

12 FINANCE AND INSURANCE

The sponsor will provide safety insurance that meets regulatory and legal requirements. The sponsor has purchased liability insurance for this clinical trial, and the liability insurance policy complies with laws and requirements. The liability insurance policy will be submitted to ERC/IRB or regulatory authorities as required.

13 PUBLICATION AND DATA SHARING POLICY

Before writing the manuscript, the author should be identified. Unless approved by Sinocelltech, Ltd., personal writing or publication is not allowed before the final report of this study is completed. Sinocelltech, Ltd. has the final decision regarding the manuscript and publication.

14 APPENDIX

14.1 APPENDIX I: WOMEN OF CHILD-BEARING POTENTIAL (WOCBP)

WOCBP are defined as women who are in the period from menarche to menopause or permanent sterilization. Additional assessments are required for women whose fertility status is unknown and whose menstrual cycle cannot be clarified prior to administration of the study vaccine.

Women who meet following criteria are not WOCBP:

1) Without menarche.
2) With one of the following conditions prior to menopause:
    - Receipt of hysterectomy;
    - Receipt of bilateral salpingectomy;
    - Receipt of bilateral oophorectomy.

For permanent infertility that is not caused by the above reasons (e.g., Müllerian agenesis, androgen insensitivity), the use of contraception is determined by the investigator.

Note: Subjects' medical records, medical examinations and medical history inquiries will be reviewed to determine whether they belong to the above types, and the corresponding information will be recorded in the study medical records.

3) Postmenopausal women:

Menopausal status is defined as the absence of menstruation for 12 consecutive months without other medical explanation. In addition,

- Women who are receiving hormone replacement therapy (HRT) with suspected menopausal status who wish to continue HRT during the trial will need to use a highly effective non-estrogenic method of contraception. Otherwise, HRT must be discontinued for confirmation of menopausal status prior to enrollment in the study.

14.2 APPENDIX II: GUIDELINE FOR ADVERSE EVENT GRADING CRITERIA IN CLINICAL TRIAL OF PREVENTIVE VACCINES--NMPA STANDARD

Table 22 Grading of Injection Site (Local) Adverse Events

Symptoms/Signs	Grade 1	Grade 2	Grade 3	Grade 4
**Pain, tenderness** (used alternatively; tenderness is used for subjects unable to express pain autonomously)				
Pain	Having no or slight influence on limb activities	Having influence on limb activities	Having influence on daily life	Loss of basic self-care, or hospitalization
Tenderness	Resisting, withdrawing after contact or touch	Crying after contact or touch, but can be soothed	Continuous crying and cannot to be soothed	Requiring emergency treatment or hospitalization
**Induration*, swelling** (used alternatively)** #				
> 14 years	In a diameter of 2.5 to < 5 cm or an area of 6.25 to < 25 cm^2, and not having no or slight influence on daily activities	In a diameter of 5 to < 10 cm or an area of 25 to < 100 cm^2, or affecting daily activities	In a diameter of ≥10 cm or an area of ≥100 cm^2, or ulceration or secondary infection or phlebitis or aseptic abscess or wound drainage or severely affecting daily activities	Abscess, exfoliative dermatitis, dermis or deep tissue necrosis
≤ 14 years	In a diameter of < 2.5 cm	In a diameter of ≥ 2.5 cm and an area of < 50% of the vaccinated limb (anatomically, the limb where the administration site is located, e.g. upper arm or thigh)	In an area of ≥ 50% of the vaccinated limb, or ulceration or secondary infection or phlebitis or wound drainage	Abscess, exfoliative dermatitis, dermis or deep tissue necrosis
**Skin rash*, flushing** (used alternatively)** #				
> 14 years	In a diameter of 2.5 to < 5 cm or an area of 6.25 to < 25 cm^2, and not having no or slight influence on daily activities	In a diameter of 5 to < 10 cm or an area of 25 to < 100 cm^2, or affecting daily activities	In a diameter of ≥10 cm or an area of ≥100 cm2, or ulceration or secondary infection or phlebitis or aseptic abscess or wound drainage or severely affecting daily activities	Abscess, exfoliative dermatitis, dermis or deep tissue necrosis
≤ 14 years	In a diameter of < 2.5 cm	In a diameter of ≥ 2.5 cm and an area of < 50% of the vaccinated limb (anatomically, the limb where the administration site is located, e.g. upper arm or thigh)	In an area of ≥ 50% of the vaccinated limb, or ulceration or secondary infection or phlebitis or wound drainage	Abscess, exfoliative dermatitis, dermis or deep tissue necrosis

**Others**				
Pruritus	Injection site pruritus which is relieved spontaneously or within 48 h after treatment	Injection site pruritus which is not relieved within 48 h after treatment	Having influence on daily life	NA
Cellulitis	NA	Requiring non-injectable treatment (e.g., oral antibiotics, antifungals, antivirals)	Requiring IV treatment (e.g., IV antibiotics, antifungals, antivirals)	Sepsis, or tissue necrosis, etc.

Note:

* In addition to direct measurement of diameter for grading evaluation, progression changes of the measurements should also be recorded.

** The maximum measured diameter or area should be used.

For evaluation and grading of induration, swelling, skin rash and flushing, indicators with higher grade are used, based on the functional grades and practical measurement results.

Table 23 Grading of Vital Signs

Signs	Grade 1	Grade 2	Grade 3	Grade 4
**Fever*** [axillary temperature (°C)]				
> 14 years	37.3 to < 38.0	38.0 to < 38.5	38.5 to < 39.5	≥ 39.5, persisting for more than 3 days
≤ 14 years	37.5 to < 38.0	38.0 to < 39.5	≥ 39.5	≥ 39.5, persisting for more than 5 days
**PR interval prolongation or atrioventricular block on ECG** (used alternatively)				
> 16 years	PR interval of 0.21 to < 0.25 s	PR interval of ≥ 0.25 s or second-degree atrioventricular block Type I	Second-degree atrioventricular block type II or ventricular interval of ≥ 3 s	Complete atrioventricular block
≤ 16 years	First-degree atrioventricular block (PR interval > normal value for others of same age and same type)	Second-degree atrioventricular block Type I	Second-degree atrioventricular block type II or ventricular interval of ≥ 3 s	Complete atrioventricular block
**Signs**	**Grade 1**	**Grade 2**	**Grade 3**	**Grade 4**
**Heart rate**				
Tachycardia (beats/min)	101–115	116-130	> 130	Arrhythmias requiring emergency treatment or hospitalization
Bradycardia (beats/min)	50-54	45-49	< 45	Arrhythmias requiring emergency treatment or hospitalization
**Blood pressure**				
Hypertension (mmHg)				
≥18 years	Systolic blood pressure: 140 to < 160, or diastolic blood pressure: 90 to < 100	Systolic blood pressure: ≥ 160 to < 180, or diastolic blood pressure: ≥ 100 to < 110	Systolic blood pressure: ≥ 180, or diastolic blood pressure: ≥ 110	Development of previously undiagnosed life-threatening complications (e.g., malignant hypertension), or hospitalization
< 18 years	Systolic blood pressure: > 120 to < 152, or diastolic blood pressure: > 80 to < 95	Systolic blood pressure: > 152 to < 178, or diastolic blood pressure: > 80 to < 95 95 to < 109	Systolic blood pressure: ≥ 178, or diastolic blood pressure: ≥ 109	Development of previously undiagnosed life-threatening complications (e.g., malignant hypertension), or hospitalization
Hypotension (systolic blood pressure) (mmHg)	85 to < 89	80 to < 85	< 80	Shock or hospitalization
Respiratory rate (breaths/minute)	17-20	21-25	> 25	Requiring endotracheal intubation

Notes:

* Axillary temperature is generally used in China, which is converted to oral temperature and anal temperature if necessary. Generally, oral temperature = axillary temperature + 0.2°C; anal temperature = axillary temperature + (0.3–0.5°C). When persistent high fever occurs, corresponding causes should be confirmed as soon as possible.

Table 24 Grading of Non-Injection-Site (Systematic) Adverse Events

Organ system symptoms/signs	Grade 1	Grade 2	Grade 3	Grade 4
**Gastrointestinal system**				
Diarrhea	Slight or transient, 3–4 times/day, with abnormal fecal characteristics or slight diarrhea persisting for less than 1 week	Moderate or persistent, 5–7 times/day, with abnormal fecal characteristics or diarrhea persisting for more than 1 week	> 7 times/day, with abnormal fecal characteristics, or hemorrhagic diarrhea, orthostatic hypotension, electrolyte imbalance, requiring intravenous fluid of > 2 L	Hypotensive shock, requiring Hospitalization
Constipation*	Requiring a stool softener and diet modification	Requiring laxatives	Intractable constipation, requiring manual dredge or use of enemas	Toxic megacolon or intestinal obstruction
Dysphagia	Mild discomfort during swallowing	Restricted diet	Severely restricted diet and conversation; unable to eat solid food	Unable to eat liquid food; requiring intravenous nutrition
Anorexia	Decreased appetite without decreased food intake	Decreased appetite and decreased food intake without significant body weight loss	Decreased appetite with significant body weight loss	Requiring interventional measures (e.g., gastric tube feeding and parenteral nutrition)
Vomiting	1–2 times/24 h and not affecting activities	3–5 times/24 h or limited activities	> 6 times within 24 h or requiring intravenous fluid infusion	Requiring hospitalization or nutrition obtained from other routes due to hypotensive shock
Nausea	Transient (< 24 h) or intermittent, while food intake basically normal	Persistent nausea leading to decreased food intake (24–48 h)	Persistent nausea leading to little food intake (> 48 h) or requiring intravenous fluid infusion	Life-threatening (e.g., hypotensive shock)
**Organ system symptoms/signs**	**Grade 1**	**Grade 2**	**Grade 3**	**Grade 4**
**Musculoskeletal and connective tissue**				
Muscle pain (non-injection site)	Not affecting daily activities	Slightly affecting daily activities	Severe muscle pain, severely affecting daily activities	Emergency treatment or hospitalization
Arthritis	Mild pain with inflammation, erythema or joint swelling, not interfering with functioning	Moderate pain with inflammation, erythema or joint swelling, interfering with functioning but not affecting daily activities	Severe pain with inflammation, erythema or joint swelling; affecting daily activities	Permanent and/or disabling joint injury
Joint pain	Mild pain, not interfering with functioning	Moderate pain; requiring analgesics and/or pain interfering with functioning but not affecting daily activities	Severe pain; requiring analgesics and/or pain affecting daily activities	Disabling pain

**Nervous system**				
Headache	Not affecting daily activities, requiring no treatment	Transient, slightly affecting daily activities, possibly requiring treatment or intervention	Severely affecting daily activities, requiring treatment or intervention	Refractory, requiring emergency treatment or hospitalization
Syncope	Near syncope without loss of consciousness (e.g., pre-syncope)	Loss of consciousness, not requiring treatment	Loss of consciousness, requiring treatment or hospitalization	NA
Seizure (newly occurred)				
⩾18 years	NA	NA	1 to 3 seizures	Prolonged and repetitive seizures (e.g., status epilepticus) or having difficulty in control (e.g., intractable epilepsy)
< 18 years	Duration of seizure of < 5 min, and post-seizure status of < 24 h	Duration of seizure of ⩾ 5 to < 20 min, and post-seizure status of < 24 h	Duration of seizure of ⩾ 20 min, or post-seizure status of > 24 h	Prolonged and repetitive seizures (e.g., status epilepticus) or having difficulty in control (e.g., intractable epilepsy)
**Respiratory system**				
Cough	Transient, requiring no treatment	Persistent cough which is responsive to treatment	Paroxysmal cough which cannot be controlled by treatment	Emergency treatment or hospitalization
Acute bronchospasm	Transient; requiring no treatment; FEV_1% of 70%–80%	Requiring treatment; returning to normal with bronchodilator therapy; FEV_1% of 50%–70%	Failure to return to normal with bronchodilator therapy; FEV_1% of 25%–50%, or persistent intercostal depression	Cyanosis; FEV_1% < 25%; or requiring intubation
Dyspnea	Dyspnea during exercise	Dyspnea during normal activities	Dyspnea at rest	Dyspnea, requiring oxygen inhalation, hospitalization, or assisted respiration
**Organ system symptoms/signs**	**Grade 1**	**Grade 2**	**Grade 3**	**Grade 4**
**Skin and subcutaneous tissues**				
Non-injection site pruritus (without skin injury)	Mild pruritus, not affecting or slightly affecting daily activities	Pruritus affecting daily activities	Pruritus resulting in inability to carry out daily activities	NA
Mucocutaneous abnormalities	Erythema/pruritus/color changed	Diffuse skin rash/maculo-papular rash/dryness/desquamation	Herpes/exudation/desquamation/ulceration	Exfoliative dermatitis involving mucosa, or polymorphic erythema, or suspected Stevens-Johnsons syndrome
**Mental system**				

Insomnia*	Mild difficulty in falling asleep, not affecting or slightly affecting daily activities	Moderate difficulty in falling asleep, affecting daily activities	Severe difficulty in falling asleep, severely affecting daily activities, requiring treatment or hospitalization	NA
Irritation or inhibition	Mild irritation or mild inhibition	Irritability or somnolence	Inability to soothe or poor response	NA
Mental disorders Mental disorders (including anxiety, depression, mania, and confusion) which shall be reported in detail	Mild symptoms not requiring a hospital visit, or behaviors not affecting or slightly affecting daily activities	Symptoms requiring a hospital visit, or behaviors affecting daily activities	Requiring hospitalization or incapacity to support daily activities	Tendency to harm self or others or acute psychosis or loss of basic self-care
Immune system				
Acute allergic reactions**	Localized urticaria (blisters), requiring no treatment	Localized urticaria requiring treatment, or mild angioedema requiring no treatment	Extensive urticaria or angioedema requiring treatment, or mild bronchospasm	Anaphylactic shock or life-threatening bronchospasm or laryngeal edema
**Other**				
Fatigue, asthenia	Not affecting daily activities	Affecting normal daily activities	Severely affecting daily activities, unable to work	Emergency treatment or hospitalization
Non-vaccination site pain#<br>(clarify the sites of pain when reporting)	Mild pain, not affecting or slightly affecting daily activities	Pain affecting daily activities	Pain resulting in inability to carry out daily activities	Pain leading to disability with loss of basic self-care

Notes:

FEV_1% refers to forced expiratory volume in the first second/forced vital capacity (FVC).

* For constipation and insomnia, attention shall be paid to any change occurred prior to and after vaccination.

** refers to Type I hypersensitivity reactions. # refers to non-vaccination site pain other than muscle pain, joint pain and headache.

Table 25 Grading of Blood Biochemistry Indicators

Test indicator	Grade 1	Grade 2	Grade 3	Grade 4
Liver function (increased ALT and AST)	1.25 to < 2.5 × ULN	2.5 to < 5.0 × ULN	5.0 to < 10 × ULN	≥ 10 × ULN
Increased total bilirubin (mg/dL; μmol/L)				
> 28 days of age	1.1 to < 1.6 × ULN	1.6 to < 2.6 × ULN	2.6 to 5.0 × ULN	≥ 5.0 × ULN
7 to ≤ 28 days of age (breastfeeding)	5 to < 10<br>85.5 to < 171	10 to < 20<br>171 to < 342	20 to < 25<br>342 to < 427.5	≥ 25<br>≥ 427.5
7 to ≤ 28 days of age (not breastfeeding)	1.1 to < 1.6 × ULN	1.6 to < 2.6 × ULN	2.6 to 5.0 × ULN	≥ 5.0 × ULN
72 h to < 7 days of age	11 to < 16<br>188.1 to < 273.6	16 to < 18<br>273.6 to < 307.8	18 to < 24<br>307.8 to < 410.4	≥ 24<br>≥ 410.4
48 to < 72 h	8.5 to < 13<br>145.5 to < 222.3	13 to < 15<br>222.3 to < 256.5	15 to < 22<br>256.5 to < 376.2	≥ 22<br>≥ 376.2
24 to < 48 h	5 to < 8<br>85.5 to < 136.8	8 to < 12<br>136.8 to < 205.2	12 to < 19<br>205.2 to < 324.9	≥ 19<br>≥ 324.9
< 24 h	4 to < 7<br>68.4 to < 119.7	7 to < 10<br>119.7 to < 171	10 to < 17<br>171 to < 290.7	≥ 17<br>≥ 290.7
Pancreatin (amylase, lipase)	1.1 to < 1.5 × ULN	1.5 to < 3.0 × ULN	3.0 to < 5.0 × ULN	≥ 5.0 × ULN
Creatine phosphokinase (CPK)	1.25 to < 1.5 × ULN	1.5 to < 3.0 × ULN	3.0 to < 10 × ULN	≥ 10 × ULN
Hypernatremia (Na, mmol/L)	146 to < 150	150 to < 154	154 to < 160	≥ 160
Hyponatraemia (Na, mmol/L)	130 to < 135	125 to < 130	121 to < 125	≤ 120
Hyperkalemia (K, mmol/L)	5.6 to < 6.0	6.0 to < 6.5	6.5 to < 7.0	≥ 7.0
Hypokalaemia (K, mmol/L)	3.0 to < 3.4	2.5 to < 3.0	2.0 to < 2.5	< 2.0
Hypercalcemia (Ca, mmol/L)				
≥ 7 days of age	2.65 to < 2.88	2.88 to < 3.13	3.13 to < 3.38	≥ 3.38
< 7 days of age	2.88 to < 3.10	3.10 to < 3.23	3.23 to < 3.38	≥ 3.38
Hypocalcemia (Ca, mmol/L)				
≥ 7 days of age	1.95 to < 2.10	1.75 to < 1.95	1.53 to < 1.75	< 1.53
< 7 days of age	1.63 to < 1.88	1.50 to < 1.63	1.38 to < 1.50	< 1.38
Hyperglycemia (Glu, mmol/L)				
Fasting	6.11 to < 6.95	6.95 to < 13.89	13.89 to < 27.75	≥ 27.75
Non-fasting	6.44 to < 8.89	8.89 to < 13.89	13.89 to < 27.75	≥ 27.75
Hypoglycemia (Glu, mmol/L)				
≥ 1 month of age	3.05 to < 3.55	2.22 to < 3.05	1.67 to < 2.22	< 1.67
< 1 month of age	2.78 to < 3.00	2.22 to < 2.78	1.67 to < 2.22	< 1.67

Note: ULN (upper limits of normal) refers to the upper limit of the range of normal values.

Table 26 Grading of Hematology Indicators

Test indicator/grading	Grade 1	Grade 2	Grade 3	Grade 4
Increased white blood cells (WBC, 10^9/L)	11 to < 13	13 to < 15	15 to < 30	≥ 30
Decreased white blood cells (WBC, 10^9/L)				
> 7 days of age	2.000-2.499	1.500-1.999	1.000-1.499	< 1.000
≤ 7 days of age	5.500-6.999	4.000-5.499	2.500-3.999	< 2.500
Decreased lymphocyte count (LY, 10^9/L)	0.75-1.00	0.5-0.749	0.25-0.49	< 0.25
Decreased neutrophil count (ANC, 10^9/L)				
> 7 days of age	0.800-1.000	0.600-0.799	0.400-0.599	< 0.400
2–7 days of age	1.250-1.500	1.000-1.249	0.750-0.999	< 0.750
≤ 1 days of age	4.000-5.000	3.000-3.999	1.500-2.999	< 1.500
Eosinophils (Eos, 10^9/L)	0.65-1.5	1.51-5.0	> 5.0	Hypereosinophilic syndrome
Thrombocytopenia (PLT, 10^9/L)				
> 12 years	125-140	100-124	25-99	< 25
> 3 months of age to ≤ 12 years	NA	50-75	25-49	< 25
Low hemoglobin (g/dL)				
males ≥ 13 years	10.0-10.9	9.0 to < 10.0	7.0 to < 9.0	< 7.0
females ≥ 13 years	9.5-10.4	8.5 to < 9.5	6.5 to < 8.5	< 6.5
57 days of age to < 13 years (males and females)	9.5-10.4	8.5 to < 9.5	6.5 to < 8.5	< 6.5
36–56 days of age (males and females)	8.5-9.6	7.0 to < 8.5	6.0 to < 7.0	< 6.0
22–35 days of age (males and females)	9.5-11.0	8.0 to < 9.5	6.7 to < 8.0	< 6.7
8–21 days of age (males and females)	11.0-13.0	9.0 to < 11.0	8.0 to < 9.0	< 8.0
≤ 7 days of age (males and females)	13.0-14.0	10.0 to < 13.0	9.0 to < 10.0	< 9.0

Table 27 Grading of Urinalysis Indicators

Test indicator	Grade 1	Grade 2	Grade 3	Grade 4
Urine protein (PRO) (Urine dipstick test)	1+	2+	3+ or above	NA
Urine glucose (urine dipstick test)	Minimal to 1+ or ≤ 250 mg	2+ or > 250 to ≤ 500 mg	> 2+ or > 500 mg	NA
Red blood cell (microscopic examination) [Red blood cell count under each high power field (rbc/hpf) (except menstrual phase in females)]	6 to < 10	≥ 10	Visible hematuria with or without blood clot; or urine red blood cell cast; or requiring treatment	Emergency treatment or hospitalization

Intensity of AEs not involved in the grading table are evaluated according to the following criteria.

Table 28 General Rules for Severity Grading of Other Adverse Events

Grade 1	Grade 2	Grade 3	Grade 4	Grade 5
Mild: Short-term (< 48 h) or slight discomfort, not affecting activities, requiring no treatment	Moderate: Mild or moderate activity restricted; possibly requiring a visit, and requiring no or mild treatment	Severe: Activities significantly restricted, requiring a hospital visit and treatment, and possibly requiring hospitalization	Critical: Possibly threatening life, activities severely restricted, requiring intensive treatment	Death

15 REFERENCES

1. *World Health Organization*. Available from: https://covid19.who.int/.
2. *COVID-19 vaccine tracker and landscape [EB/OL]. [2023-3-10]. https://www.who.int/publications/m/item/draft-landscape-of-covid-19-candidate-vaccines*
3. *WHO-COVID19 Vaccine Tracker [EB/OL]. [2022-10-21]. https://covid19.trackvaccines.org/agency/who/.*
4. *Centers for Disease Control and Prevention. COVID-19 Data Tracker: Variant Proportions. 2023.4.8. https://covid.cdc.gov/covid-data-tracker/#variant-proportions*.
5. Davis-Gardner, M.E., et al., *Neutralization against BA.2.75.2, BQ.1.1, and XBB from mRNA Bivalent Booster.* N Engl J Med, 2023. **388**(2): p. 183-185.
6. *National Health Commission of the People's Republic of China. Diagnosis and treatment plan for COVID-19 (trial version 8).* Chin J Clin Infect Dis., 2020. **13**(5).
7. *General Office of National Health Commission of People's Republic of China. Diagnosis and Treatment Protocol for Novel Coronavirus Pneumonia (Trial Version 10). 2023.01.05.*
8. *Sinocelltech Ltd. COVID-19 Alpha/Beta/Delta/Omicron Variants S-Trimer Vaccine (SCTV01E) Investigator's Brochure.*
9. *Center for Drug Evaluation. Technical Guidance for Research & Development and Evaluation of Prophylactic Vaccines Against SARS-CoV-2 Variants (Interim). 2021.09.*
10. *https://www.ema.europa.eu/en/documents/scientific-guideline/reflection-paper-regulatory-requirements-vaccines-intended-provide-protection-against-variant_en.pdf.*
11. *National Medical Product Administration. General Principles of Technical Review for Preclinical Safety Assessment of Prophylactic Biologics.*
12. Karim, S.S.A., *Vaccines and SARS-CoV-2 variants: the urgent need for a correlate of protection.* Lancet, 2021. **397**(10281): p. 1263-1264.
13. Wang, P., et al., *Antibody resistance of SARS-CoV-2 variants B.1.351 and B.1.1.7.* Nature, 2021. **593**(7857): p. 130-135.
14. Abu-Raddad, L.J., et al., *Effectiveness of the BNT162b2 Covid-19 Vaccine against the B.1.1.7 and B.1.351 Variants.* N Engl J Med, 2021. **385**(2): p. 187-189.
15. Shinde, V., et al., *Efficacy of NVX-CoV2373 Covid-19 Vaccine against the B.1.351 Variant.* N Engl J Med, 2021. **384**(20): p. 1899-1909.
16. Madhi, S.A., et al., *Efficacy of the ChAdOx1 nCoV-19 Covid-19 Vaccine against the B.1.351 Variant.* N Engl J Med, 2021. **384**(20): p. 1885-1898.
17. *A study to evaluate the immunogenicity and safety of mRNA-1273.211 vaccine for COVID-19 variants. ClinicalTrials.gov (NCT04927065).*
18. *The Phase I Clinical trial of booster vaccination of adenovirus type-5 vectored COVID-19 vaccine. ClinicalTrials.gov (NCT04568811).*
19. *Immunogenicity and safety of a third dose, and immune persistence of CoronaVac vaccine in healthy adults aged 18-59 years: interim results from a double-blind, randomized, placebo-controlled phase 2 clinical trial. ClinicalTrials.gov (NCT04979949).*
20. Borobia, A.M., et al., *Immunogenicity and reactogenicity of BNT162b2 booster in ChAdOx1-S-primed participants (CombiVacS): a multicentre, open-label, randomised, controlled, phase 2 trial.* Lancet, 2021. **398**(10295): p. 121-130.
21. *Protocol template to be used as template for observational study protocols for sentinel surveillance of adverse events of special interest (AESIs) after vaccination with COVID-19 vaccines. Geneva: World Health Organization; 2021. Licence: CC BY-NC-SA 3.0 IGO.*
