# Supplementary material for: Immunogenicity of Tetravalent Protein Vaccine SCTV01E-2 against SARS-CoV-2 EG.5 Subvaraint: A Phase 2 Trial
Source: Vaccines (Basel). 2024 Feb 8;12(2):175. doi: 10.3390/vaccines12020175 (PMC10893468; doi:10.3390/vaccines12020175)
Supplement: Supplementary file 1 [file vaccines-12-00175-s001.zip › vaccines-2795204-Supplementary Materials S1.pdf]

# Supplementary Materials

Supplementary to: Tang J, Zhu C, Xu Q, et al. Immunogenicity of Tetravalent Protein Vaccine, SCTV01E-2 against SARS-CoV-2 EG.5 Subvaraint: a Phase 2 Trial.

This appendix has been provided by the authors to give readers additional information about the work.

Supplementary Materials ........................................................................................1
**Table S1.** Demographic characteristics of participants in the Immunogenicity Per-Protocol Set ........................................................................................3
**Table S2.** Geometric Mean Titer (GMT) of Neutralizing Antibodies Against Omicron EG.5 and XBB.1 variants in the Full Analysis Set ........................................................5
**Table S3.** Seroconversion Rate (SRR) of Neutralizing Antibodies Against Omicron EG.5 and XBB.1 variants in the Full Analysis Set ........................................................6
**Table S4.** Solicited local adverse reactions of the study vaccines................................7
**Table S5.** Solicited systemic adverse reactions of the study vaccines ..........................8
**Figure S1.** Subgroup analysis of geometric mean titer (GMT) and the least square geometric mean ratio (LS GMR) of live virus nAb against Omicron EG.5. ...............10
**Figure S2.** Subgroup analysis of seroresponse rate (SRR) of Live Virus nAb against Omicron EG.5. ........................................................................................11
**Figure S3.** Subgroup analysis of geometric mean titer (GMT) and the least square geometric mean ratio (LS GMR) of live virus nAb against Omicron XBB.1. ............12
Abbreviations: GMT, geometric mean titer; LS GMR, least square geometric mean ratio. ........................................................................................12
**Figure S4.** Subgroup analysis of seroresponse rate (SRR) of live virus nAb against Omicron XBB.1. ........................................................................................13

**Table S1.** Demographic characteristics of participants in the Immunogenicity Per-Protocol Set

| | SCTV01E (N = 196) n (%) | SCTV01E-2 (N = 203) n (%) | Total (N = 399) n (%) |
|---|---|---|---|
| **Age (Year)** | | | |
| N | 196 | 203 | 399 |
| Mean (SD) | 55.9 (12.5) | 55.0 (11.7) | 55.4 (12.1) |
| Median (Min, Max) | 57.5 (20, 81) | 58.0 (19, 83) | 58.0 (19, 83) |
| **Age subgroups-randomization, n (%)** | | | |
| 18-59 years | 120 (61.2) | 121 (59.6) | 241 (60.4) |
| ≥60 years | 76 (38.8) | 82 (40.4) | 158 (39.6) |
| **Sex, n (%)** | | | |
| Male | 91 (46.4) | 93 (45.8) | 184 (46.1) |
| Female | 105 (53.6) | 110 (54.2) | 215 (53.9) |
| **Nation, n (%)** | | | |
| Han | 195 (99.5) | 203 (100.0) | 398 (99.7) |
| Others | 1 (0.5) | 0 | 1 (0.3) |
| **BMI (kg/m2) ‡** | | | |
| N | 196 | 203 | 399 |
| Mean (SD) | 26.0 (3.5) | 26.5 (3.5) | 26.3 (3.5) |
| Median (Min, Max) | 25.8 (18.9, 38.6) | 26.6 (16.4, 39.8) | 26.1 (16.4, 39.8) |
| **History of SARS-CoV-2 infection, n (%)** | | | |
| Yes | 53 (27.0) | 52 (25.6) | 105 (26.3) |
| No | 143 (73.0) | 151 (74.4) | 294 (73.7) |
| **Previous vaccination/infection interval, n (%)** | | | |
| 6-11 months | 62 (31.6) | 63 (31.0) | 125 (31.3) |
| ≥12 months | 134 (68.4) | 140 (69.0) | 274 (68.7) |
| **IgM at baseline** | | | |
| Positive | 0 | 0 | 0 |

| | SCTV01E (N = 196) n (%) | SCTV01E-2 (N = 203) n (%) | Total (N = 399) n (%) |
|---|---|---|---|
| Negative | 196 (100.0) | 203 (100.0) | 399 (100.0) |
| **Booster dose of COVID-19 vaccine, n (%)** | | | |
| Yes | 143 (73.0) | 145 (71.4) | 288 (72.2) |
| No | 53 (27.0) | 58 (28.6) | 111 (27.8) |
| **Type of last received COVID-19 vaccine-randomization, n (%)** | | | |
| Inactive vaccine | 91 (46.4) | 97 (47.8) | 188 (47.1) |
| Adenovirus vector vaccine | 38 (19.4) | 33 (16.3) | 71 (17.8) |
| Recombinant protein vaccine | 67 (34.2) | 73 (36.0) | 140 (35.1) |
| Other vaccines | 0 | 0 | 0 |
| **Pre-existing comorbidities, n (%)** | | | |
| Yes | 74 (37.8) | 90 (44.3) | 164 (41.1) |
| No | 122 (62.2) | 113 (55.7) | 235 (58.9) |

‡ BMI, the body mass index. The body-mass index is the weight in kilograms divided by the square of the height in meters.

**Table S2.** Geometric Mean Titer (GMT) of Neutralizing Antibodies Against Omicron EG.5 and XBB.1 variants in the Full Analysis Set

| | SCTV01E (N = 215) | SCTV01E-2 (N = 214) |
|---|---|---|
| **EG.5** | | |
| Baseline (Day 0) | | |
| n | 209 | 209 |
| GMT (95% CI) | 94 (78, 112) | 103 (90, 119) |
| Day 14 | | |
| n | 207 | 209 |
| GMT (95% CI) | 514 (459, 574) | 922 (825, 1032) |
| GMFI (95% CI) | 5.5 (4.6, 6.4) | 8.9 (7.6, 10.4) |
| LS GMR (95% CI)† | | 1.76 (1.52, 2.04) |
| P value† | | <0.001 |
| **XBB.1** | | |
| Baseline (Day0) | | |
| n | 213 | 214 |
| GMT (95% CI) | 270 (233, 312) | 324 (288, 364) |
| Day 14 | | |
| n | 211 | 214 |
| GMT (95% CI) | 1422 (1264, 1600) | 1879 (1682, 2100) |
| GMFI (95% CI) | 5.3 (4.5, 6.2) | 5.8 (5.0, 6.7) |
| LS GMR (95% CI)† | | 1.28 (1.09, 1.50) |
| P value† | | 0.003 |

†The immunogenicity data were analyzed based on covariance (ANCOVA) model, covariates included intervention groups, stratification factors and baseline values (in log-transformed scale).

Abbreviations: N, number of participants in the population; n, number of participants in the specific category; GMT, geometric mean titer; GMFI: geometric mean fold increase from baseline; LS GMR, least square geometric mean ratio.

**Table S3.** Seroconversion Rate (SRR) of Neutralizing Antibodies Against Omicron EG.5 and XBB.1 variants in the Full Analysis Set

| | **SCTV01E (N = 215)** | **SCTV01E-2 (N = 214)** |
|---|---|---|
| **EG.5** | | |
| Day 14 | | |
| SRR, n/N | 59.4 (123/207) | 78.5 (164/209) |
| SRR (95% CI)† | 52.4, 66.2 | 72.3, 83.8 |
| Difference of SRRs between two groups (95% CI)‡ | | 19.07 (10.24, 27.68) |
| P value‡ | | <0.001 |
| **XBB.1** | | |
| Day 14 | | |
| SRR, n/N | 60.7 (128/211) | 68.7 (147/214) |
| SRR (95% CI)† | 53.7, 67.3 | 62.0, 74.8 |
| Difference of SRRs between two groups (95% CI)‡ | | 8.10 (-1.00, 17.08) |
| P value‡ | | 0.081 |

†Confidential interval of Clopper-Pearson.

‡estimated from the stratified Miettinen-Nurminen method, stratification based on the actual stratification factors.

Abbreviations: N, number of participants in the population; n, number of participants in the specific category; SRR, seroresponse rate.

**Table S4.** Solicited local adverse reactions of the study vaccines

| Preferred Term<br>Intensity | SCTV01E<br>(N = 215)<br>n (%) | SCTV01E-2<br>(N = 214)<br>n (%) |
|---|---|---|
| **Number of Participants with at Least One Study Vaccine-Related Solicited Local Adverse Event** | 60 (27.9) | 59 (27.6) |
| Grade 1 | 51 (23.7) | 49 (22.9) |
| Grade 2 | 9 (4.2) | 10 (4.7) |
| Grade 3 and above | 0 | 0 |
| **Pain** | 52 (24.2) | 52 (24.3) |
| Grade 1 | 47 (21.9) | 48 (22.4) |
| Grade 2 | 5 (2.3) | 4 (1.9) |
| Grade 3 and above | 0 | 0 |
| **Pruritus** | 9 (4.2) | 5 (2.3) |
| Grade 1 | 9 (4.2) | 5 (2.3) |
| Grade 2 | 0 | 0 |
| Grade 3 and above | 0 | 0 |
| **Swelling** | 5 (2.3) | 8 (3.7) |
| Grade 1 | 3 (1.4) | 3 (1.4) |
| Grade 2 | 2 (0.9) | 5 (2.3) |
| Grade 3 and above | 0 | 0 |
| **Erythema** | 1 (0.5) | 4 (1.9) |
| Grade 1 | 0 | 2 (0.9) |
| Grade 2 | 1 (0.5) | 2 (0.9) |
| Grade 3 and above | 0 | 0 |
| **Induration** | 1 (0.5) | 1 (0.5) |
| Grade 1 | 0 | 1 (0.5) |
| Grade 2 | 1 (0.5) | 0 |
| Grade 3 and above | 0 | 0 |

MedDRA Version 26.0

Abbreviations: N, number of participants in the population; n, number of participants in the specific category; %, percentage of participants with N as the denominator.

**Table S5.** Solicited systemic adverse reactions of the study vaccines

| **Preferred Term**<br>**Intensity** | **SCTV01E (N = 215) n (%)** | **SCTV01E-2 (N = 214) n (%)** |
|---|---|---|
| **Number of Participants with at Least One Study Vaccine-Related Solicited Systemic Adverse Event** | 27 (12.6) | 44 (20.6) |
| Grade 1 | 16 (7.4) | 28 (13.1) |
| Grade 2 | 10 (4.7) | 10 (4.7) |
| Grade 3 and above | 1 (0.5) | 6 (2.8) |
| **Pyrexia** | 17 (7.9) | 25 (11.7) |
| Grade 1 | 12 (5.6) | 17 (7.9) |
| Grade 2 | 4 (1.9) | 3 (1.4) |
| Grade 3 and above | 1 (0.5) | 5 (2.3) |
| **Fatigue** | 8 (3.7) | 18 (8.4) |
| Grade 1 | 4 (1.9) | 13 (6.1) |
| Grade 2 | 4 (1.9) | 5 (2.3) |
| Grade 3 and above | 0 | 0 |
| **Headache** | 9 (4.2) | 16 (7.5) |
| Grade 1 | 5 (2.3) | 10 (4.7) |
| Grade 2 | 4 (1.9) | 6 (2.8) |
| Grade 3 and above | 0 | 0 |
| **Myalgia** | 4 (1.9) | 9 (4.2) |
| Grade 1 | 2 (0.9) | 6 (2.8) |
| Grade 2 | 2 (0.9) | 3 (1.4) |
| Grade 3 and above | 0 | 0 |
| **Nausea** | 4 (1.9) | 6 (2.8) |
| Grade 1 | 3 (1.4) | 5 (2.3) |
| Grade 2 | 1 (0.5) | 1 (0.5) |
| Grade 3 and above | 0 | 0 |
| **Arthritis** | 3 (1.4) | 4 (1.9) |
| Grade 1 | 1 (0.5) | 1 (0.5) |
| Grade 2 | 2 (0.9) | 2 (0.9) |
| Grade 3 and above | 0 | 1 (0.5) |
| **Vomit** | 0 | 1 (0.5) |
| Grade 1 | 0 | 0 |
| Grade 2 | 0 | 1 (0.5) |
| Grade 3 and above | 0 | 0 |
| **Chill** | 1 (0.5) | 0 |
| Grade 1 | 0 | 0 |
| Grade 2 | 1 (0.5) | 0 |
| Grade 3 and above | 0 | 0 |

MedDRA Version 26.0

Abbreviations: N, number of participants in the population; n, number of participants

in the specific category; %, percentage of participants with N as the denominator.

**Figure S1.** Subgroup analysis of geometric mean titer (GMT) and the least square geometric mean ratio (LS GMR) of live virus nAb against Omicron EG.5.

| | V01E GMT | V01E-2 GMT | LS GMR (95% CI) |
|---|---|---|---|
| **D14 Subgroup:** | | | |
| **Sex:** | | | |
| Male | 446 | 787 | 1.6 (1.3,2.1) |
| Female | 576 | 1061 | 1.8 (1.5,2.2) |
| **Age:** | | | |
| 18-59 years | 549 | 850 | 1.5 (1.3,1.9) |
| >=60 years | 456 | 1046 | 2.1 (1.6,2.7) |
| **COVID-19 infection history:** | | | |
| Yes | 559 | 983 | 1.6 (1.1,2.2) |
| No | 493 | 904 | 1.8 (1.5,2.1) |
| **Previous vaccination/infection interval:** | | | |
| 6-11 Months | 540 | 921 | 1.6 (1.2,2.2) |
| >=12 Months | 497 | 925 | 1.8 (1.5,2.2) |
| **Completed the SARS-CoV-2 vaccine booster immunization:** | | | |
| Yes | 524 | 945 | 1.8 (1.5,2.1) |
| No | 476 | 874 | 1.7 (1.3,2.3) |
| **Type of last received vaccine:** | | | |
| Inactive vaccine | 546 | 1089 | 2.0 (1.6,2.5) |
| Adenovirus vector vaccine | 459 | 671 | 1.3 (0.9,1.9) |
| Recombinant protein vaccine | 496 | 857 | 1.7 (1.3,2.1) |
| **Pre-existing Comorbidities:** | | | |
| Yes | 428 | 937 | 2.1 (1.7,2.6) |
| No | 568 | 914 | 1.6 (1.3,1.9) |

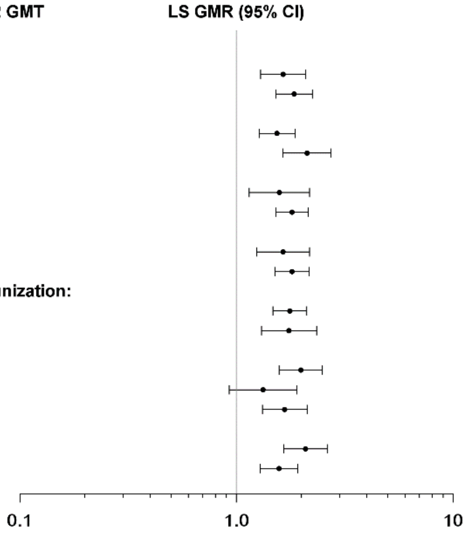


The subgroup analysis was conducted among the population in the immunogenicity per-protocol set, who had a valid immunogenicity test result prior to the administration of study vaccines and after the administration of study vaccines and had a negative result of anti-spike receptor binding domain (RBD) IgM test at baseline but without major protocol deviations.

Abbreviations: GMT, geometric mean titer; LS GMR, least square geometric mean ratio.

**Figure S2.** Subgroup analysis of seroresponse rate (SRR) of Live Virus nAb against Omicron EG.5.

| | V01E SRR | V01E-2 SRR | SRR Rate Difference (95% CI) |
|---|---|---|---|
| **D14 Subgroup:** | | | |
| **Sex:** | | | |
| Male | 62.2 (56/90) | 73.9 (68/92) | 11.7(-1.9,25.0) |
| Female | 61.0 (61/100) | 83.2 (89/107) | 22.2(10.1,33.9) |
| **Age:** | | | |
| 18-59 years | 61.7 (71/115) | 79.8 (95/119) | 18.1(6.5,29.4) |
| >=60 years | 61.3 (46/75) | 77.5 (62/80) | 16.2(1.7,30.3) |
| **COVID-19 infection history:** | | | |
| Yes | 62.7 (32/51) | 74.5 (38/51) | 11.8(-6.4,29.3) |
| No | 61.2 (85/139) | 80.4 (119/148) | 19.3(8.8,29.5) |
| **Previous vaccination/infection interval:** | | | |
| 6-11 Months | 59.3 (35/59) | 77.4 (48/62) | 18.1(1.5,34.0) |
| >=12 Months | 62.6 (82/131) | 79.6 (109/137) | 17.0(6.2,27.5) |
| **Completed the SARS-CoV-2 vaccine booster immunization:** | | | |
| Yes | 63.8 (88/138) | 81.7 (116/142) | 17.9(7.6,28.1) |
| No | 55.8 (29/52) | 71.9 (41/57) | 16.2(-1.9,33.5) |
| **Type of last received vaccine:** | | | |
| Inactive vaccine | 65.5 (57/87) | 90.5 (86/95) | 25.0(13.4,36.7) |
| Adenovirus vector vaccine | 63.2 (24/38) | 56.3 (18/32) | -6.9(-29.4,15.9) |
| Recombinant protein vaccine | 55.4 (36/65) | 73.6 (53/72) | 18.2(2.2,33.6) |
| **Pre-existing Comorbidities:** | | | |
| Yes | 63.9 (46/72) | 77.9 (67/86) | 14.0(-0.2,28.1) |
| No | 60.2 (71/118) | 79.6 (90/113) | 19.5(7.7,30.8) |

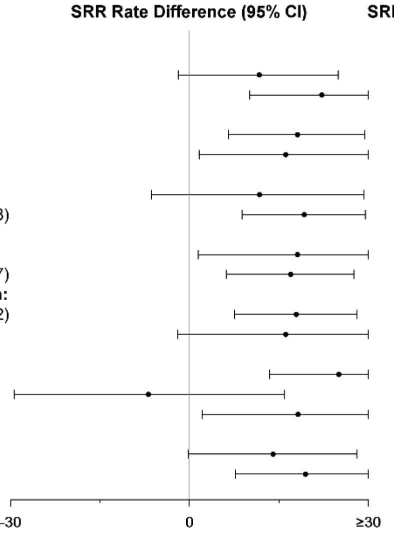


The subgroup analysis was conducted among the population in the immunogenicity per-protocol set, who had a valid immunogenicity test result prior to the administration of study vaccines and after the administration of study vaccines and had a negative result of anti-spike receptor binding domain (RBD) IgM test at baseline but without major protocol deviations. Seroresponse was defined as a change from below the low limit of quantitation (LLOQ) to equal to or above LLOQ, or more than a 4-fold rise if the baseline is equal to or above LLOQ in nAb from baseline.

Abbreviations: SRR, seroresponse rate.

**Figure S3.** Subgroup analysis of geometric mean titer (GMT) and the least square geometric mean ratio (LS GMR) of live virus nAb against Omicron XBB.1.

| | V01E GMT | V01E-2 GMT | LS GMR (95% CI) |
|---|---|---|---|
| **D14 Subgroup:** | | | |
| **Sex:** | | | |
| Male | 1352 | 1712 | 1.2 (0.9,1.5) |
| Female | 1513 | 2048 | 1.3 (1.1,1.7) |
| **Age:** | | | |
| 18-59 years | 1440 | 1754 | 1.2 (1.0,1.5) |
| >=60 years | 1429 | 2101 | 1.4 (1.0,1.8) |
| **COVID-19 infection history:** | | | |
| Yes | 1601 | 1734 | 1.0 (0.7,1.5) |
| No | 1379 | 1943 | 1.4 (1.1,1.6) |
| **Previous vaccination/infection interval:** | | | |
| 6-11 Months | 1595 | 1765 | 1.1 (0.8,1.5) |
| >=12 Months | 1367 | 1944 | 1.4 (1.1,1.7) |
| **Completed the SARS-CoV-2 vaccine booster immunization:** | | | |
| Yes | 1547 | 1875 | 1.2 (1.0,1.4) |
| No | 1170 | 1918 | 1.6 (1.2,2.1) |
| **Type of last received vaccine:** | | | |
| Inactive vaccine | 1670 | 2184 | 1.3 (1.0,1.6) |
| Adenovirus vector vaccine | 1298 | 1463 | 1.1 (0.8,1.6) |
| Recombinant protein vaccine | 1237 | 1743 | 1.3 (1.0,1.7) |
| **Pre-existing Comorbidities:** | | | |
| Yes | 1350 | 1963 | 1.4 (1.1,1.8) |
| No | 1491 | 1828 | 1.2 (1.0,1.5) |

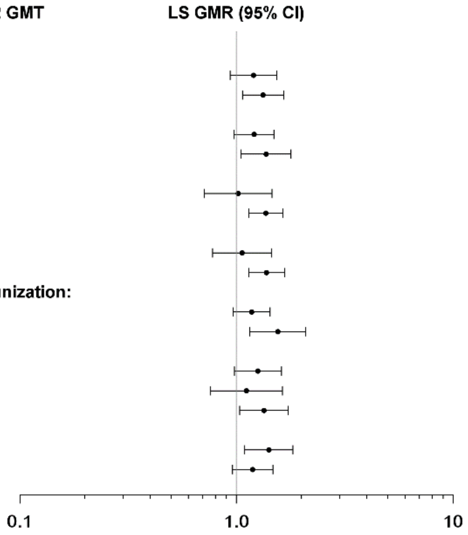


The subgroup analysis was conducted among the population in the immunogenicity per-protocol set, who had a valid immunogenicity test result prior to the administration of study vaccines and after the administration of study vaccines and had a negative result of anti-spike receptor binding domain (RBD) IgM test at baseline but without major protocol deviations.

Abbreviations: GMT, geometric mean titer; LS GMR, least square geometric mean ratio.

**Figure S4.** Subgroup analysis of seroresponse rate (SRR) of live virus nAb against Omicron XBB.1.

| D14 Subgroup: | V01E SRR | V01E-2 SRR | SRR Rate Difference (95% CI) |
|---|---|---|---|
| **Sex:** | | | |
| Male | 67.0 (61/91) | 65.6 (61/93) | -1.4(-15.0,12.2) |
| Female | 58.3 (60/103) | 70.9 (78/110) | 12.7(-0.2,25.2) |
| **Age:** | | | |
| 18-59 years | 62.7 (74/118) | 68.6 (83/121) | 5.9(-6.2,17.8) |
| >=60 years | 61.8 (47/76) | 68.3 (56/82) | 6.5(-8.4,21.2) |
| **COVID-19 infection history:** | | | |
| Yes | 61.5 (32/52) | 71.2 (37/52) | 9.6(-8.6,27.3) |
| No | 62.7 (89/142) | 67.5 (102/151) | 4.9(-6.0,15.7) |
| **Previous vaccination/infection interval:** | | | |
| 6-11 Months | 59.0 (36/61) | 68.3 (43/63) | 9.2(-7.7,25.8) |
| >=12 Months | 63.9 (85/133) | 68.6 (96/140) | 4.7(-6.6,15.8) |
| **Completed the SARS-CoV-2 vaccine booster immunization:** | | | |
| Yes | 62.0 (88/142) | 66.2 (96/145) | 4.2(-6.9,15.3) |
| No | 63.5 (33/52) | 74.1 (43/58) | 10.7(-6.7,27.8) |
| **Type of last received vaccine:** | | | |
| Inactive vaccine | 68.9 (62/90) | 73.2 (71/97) | 4.3(-8.7,17.3) |
| Adenovirus vector vaccine | 50.0 (19/38) | 54.5 (18/33) | 4.5(-18.5,27.1) |
| Recombinant protein vaccine | 60.6 (40/66) | 68.5 (50/73) | 7.9(-8.0,23.6) |
| **Pre-existing Comorbidities:** | | | |
| Yes | 54.1 (40/74) | 72.2 (65/90) | 18.2(3.4,32.5) |
| No | 67.5 (81/120) | 65.5 (74/113) | -2.0(-14.1,10.1) |

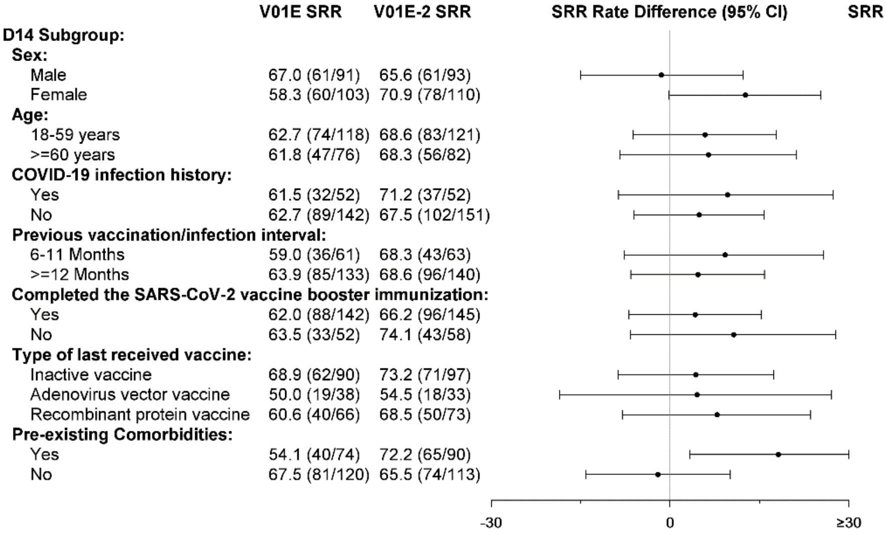


The subgroup analysis was conducted among the population in the immunogenicity per-protocol set, who had a valid immunogenicity test result prior to the administration of study vaccines and after the administration of study vaccines and had a negative result of anti-spike receptor binding domain (RBD) IgM test at baseline but without major protocol deviations. Seroresponse was defined as a change from below the low limit of quantitation (LLOQ) to equal to or above LLOQ, or more than a 4-fold rise if the baseline is equal to or above LLOQ in nAb from baseline.

Abbreviations: SRR, seroresponse rate.
